# Outcomes Among Mechanically Ventilated Patients With Severe Pneumonia and Acute Hypoxemic Respiratory Failure From SARS-CoV-2 and Other Etiologies

**DOI:** 10.1001/jamanetworkopen.2022.50401

**Published:** 2023-01-10

**Authors:** Eric P. Nolley, Sarina K. Sahetya, Chad H. Hochberg, Shakir Hossen, David N. Hager, Roy G. Brower, Elizabeth A. Stuart, William Checkley

**Affiliations:** 1 Division of Pulmonary and Critical Care, School of Medicine, Johns Hopkins University, Baltimore, Maryland; 2 Bloomberg School of Public Health, Department of Mental Health, Johns Hopkins University, Baltimore, Maryland

## Abstract

**Importance:**

Early observations suggested that COVID-19 pneumonia had a higher mortality rate than other causes of pneumonia.

**Objective:**

To compare outcomes between mechanically ventilated patients with pneumonia due to COVID-19 (March 2020 to June 2021) and other etiologies (July 2016 to December 2019).

**Design, Setting, and Participants:**

This retrospective cohort study was conducted at the Johns Hopkins Healthcare System among adult patients (aged ≥18 years) with pneumonia who required mechanical ventilation in the first 2 weeks of hospitalization. Clinical, laboratory, and mechanical ventilation data were extracted from admission to hospital discharge or death.

**Exposures:**

Pneumonia due to COVID-19.

**Main Outcomes and Measures:**

The primary outcome was 90-day in-hospital mortality. Secondary outcomes were time to liberation from mechanical ventilation, hospital length of stay, static respiratory system compliance, and ventilatory ratio. Unadjusted and multivariable-adjusted logistic regression, proportional hazards regression, and doubly robust regression were used in propensity score–matched sets to compare clinical outcomes.

**Results:**

Overall, 719 patients (mean [SD] age, 61.8 [15.3] years; 442 [61.5%] were male; 460 [64.0%] belonged to a minoritized racial group and 253 [35.2%] were White) with severe COVID-19 pneumonia and 1127 patients (mean [SD] age, 60.9 [15.8] years; 586 [52.0%] were male; 459 [40.7%] belonged to a minoritized racial group and 655 [58.1%] were White) with severe non–COVID-19 pneumonia. In unadjusted analyses, patients with COVID-19 pneumonia had higher 90-day mortality (odds ratio, 1.21, 95% CI 1.04-1.41), longer time on mechanical ventilation (subdistribution hazard ratio 0.72, 95% CI 0.63-0.81), and lower compliance (32.0 vs 28.4 mL/kg PBW/cm H_2_O; *P* < .001) when compared with those with non–COVID-19 pneumonia. In propensity score–matched analyses, patients with COVID-19 pneumonia were equally likely to die within 90 days as those with non–COVID-19 pneumonia (odds ratio, 1.04; 95% CI, 0.81 to 1.35; *P* = .85), had similar respiratory system compliance (mean difference, 1.82 mL/cm H_2_O; 95% CI, −1.53 to 5.17 mL/cm H_2_O; *P* = .28) and ventilatory ratio (mean difference, −0.05; 95% CI, −0.22 to 0.11; *P* = .52), but had lower rates of liberation from mechanical ventilation (subdistribution hazard ratio, 0.81; 95% CI, 0.65 to 1.00) when compared with those with non–COVID-19 pneumonia. Patients with COVID-19 pneumonia had somewhat lower rates of being discharged from the hospital alive at 90 days (subdistribution hazard ratio, 0.83; 95% CI, 0.68 to 1.01) than those with non–COVID-19 pneumonia; however, this was not statistically significant.

**Conclusions and Relevance:**

In this study, mechanically ventilated patients with severe COVID-19 pneumonia had similar mortality rates as patients with other causes of severe pneumonia but longer times to liberation from mechanical ventilation. Mechanical ventilation use in COVID-19 pneumonia should follow the same evidence-based guidelines as for any pneumonia.

## Key Points

**Question:**

Does COVID-19 pneumonia have a higher mortality rate than other causes of pneumonia?

**Findings:**

In this cohort study of 1846 patients with pneumonia, COVID-19 pneumonia had similar mortality rates and physiologic phenotypes as other causes of pneumonia.

**Meaning:**

These findings suggest that mechanical ventilation use in COVID-19 pneumonia should follow the same evidence-based guidelines as any pneumonia.

## Teaser

This cohort study compares outcomes between mechanically ventilated patients with pneumonia due to COVID-19 and other etiologies.

## Introduction

Acute hypoxemic respiratory failure (AHRF) is a serious complication of SARS-CoV-2 infection. Early in the pandemic, it was suggested that respiratory failure due to COVID-19 may exhibit a different physiologic phenotype and higher mortality compared with non–COVID-19 AHRF.^1,2^ This suggestion and a few small studies comparing respiratory failure due to COVID-19 and non–COVID-19 pneumonia led some clinicians to propose using nonstandard mechanical ventilation strategies.

Proponents of COVID-19 AHRF as a unique respiratory physiology phenotype suggest that strict adherence to low tidal volume ventilation may not be necessary and may even be harmful.^3^ However, if COVID-19 pneumonia leads to physiology typical of classic acute respiratory distress syndrome (ARDS), then evidence-based ARDS treatment strategies, such as low tidal volume ventilation and prone positioning, are the only interventions proven to reduce mortality. Existing studies comparing COVID-19 with non–COVID-19 pneumonia are limited by small sample size or relatively simple comparison methods leading to high risk of confounding.^4,5,6^

To better inform this debate, we performed a retrospective study of patients with severe pneumonia and AHRF due to COVID-19 and non–COVID-19 pneumonia within the Johns Hopkins Healthcare System (JHHS) to directly compare key characteristics, mortality, time to liberation from mechanical ventilation, and respiratory physiology. We also sought to understand the natural course of severe pneumonia more fully and AHRF from COVID-19 compared with that from other etiologies.

## Methods

### Study Design, Patient Population, and Data Elements

This multicenter, retrospective, observational study included adults (aged ≥18 years) with severe pneumonia and AHRF who were mechanically ventilated within 14 days of hospital admission due to COVID-19 or other etiologies. This study was approved by the institutional review board of the School of Medicine at Johns Hopkins University. Because the analysis involved retrospective data using the Johns Hopkins University Precision Medicine Analytic Platform, it was exempt from requiring informed consent. The report follows the Strengthening the Reporting of Observational Studies in Epidemiology (STROBE) reporting guidelines for observational studies.

We used data for all patients who met the aforementioned criteria and were admitted to 1 or more of 5 hospitals (2 academic, 3 community) in the JHHS, which operates a combined 2513 acute care beds and 354 intensive care unit (ICU) beds in Maryland and Washington, DC.

The patients in the COVID-19 cohort were admitted between March 1, 2020, and June 30, 2021, and tested positive for SARS-CoV-2 by polymerase chain reaction. The non–COVID-19 cohort included patients admitted between July 1, 2016, and December 31, 2019, with a recorded diagnosis of pneumonia on admission per *International Statistical Classification of Diseases and Related Health Problems, Tenth Revision *(*ICD-10*) (eAppendix 1 in Supplement 1). For both groups, we excluded patients with ventilator-associated pneumonia or who had a tracheostomy present on admission.

Data were extracted from the Johns Hopkins Precision Medicine Analytic Platform which houses comprehensive electronic health record (EHR) data, including all demographic clinical data and billing codes for all 5 JHHS hospitals. Extracted elements included baseline demographics (eg, age, sex, self-reported race [minoritized racial group or White] and ethnicity [Hispanic or not Hispanic], height, and weight), comorbidities (eg, cardiovascular disease, diabetes, chronic kidney disease, cancer, asthma, and chronic obstructive pulmonary disease [COPD] per *ICD-10* coding) (eAppendix 2 in Supplement 1), vital signs, laboratory data, and respiratory flowsheet parameters. Race and ethnicity were collected as part of patient registration at JHHS. The investigators decided a priori to collapse race into White and minoritized racial groups prior to analysis. Minoritized racial groups were American Indian or Alaska Native, Asian, Black, Native Hawaiian or other Pacific Islander, and other (nonspecified). There were no Native Hawaiian or other Pacific Islander individuals in our database. Specific respiratory parameters of interest included: oxygen delivery devices (eg, low-flow noninvasive ventilation, high-flow nasal cannula), fraction of inspired oxygen (Fio_2_), and mechanical ventilation parameters including mode, set tidal volume (Vt), plateau pressure, positive end-expiratory pressure (PEEP), and set and total respiratory rate. All ventilatory variables were collected longitudinally over the course of hospitalization. The Sequential Organ Failure Assessment (SOFA) score was calculated using the worst value of each component on the first day of mechanical ventilation.^7^ The CURB-65 score (confusion of new onset, blood urea nitrogen, respiratory rate, blood pressure, and age 65 years or older) was calculated using the worst value of each component on the first hospital day.^8^

We defined the start of mechanical ventilation by the first documentation of a ventilator mode. We used the lowest, highest, and mean values of vital signs and oxygenation parameters (ie, partial pressure of oxygen, arterial [Pao_2_], and Fio_2_) on the first day of mechanical ventilation (day 0) and daily thereafter until day 7. To reduce missingness in vital signs and laboratory values on day 0 of mechanical ventilation, baseline values were abstracted in a window that spanned between 48 hours prior to and 24 hours after the start of mechanical ventilation. For daily data, the first available mechanical ventilation parameters were used. Driving pressure was calculated as the difference between plateau pressure and set PEEP. Static respiratory compliance was calculated from other abstracted data elements (Vt / driving pressure). Ventilatory ratio was calculated using minute ventilation; partial pressure of carbon dioxide, arterial [Paco_2_]; estimated body weight; and mechanical power, as described elsewhere.^9,10^ Pao_2_/F_i_o_2_ (P/F) ratio was determined for each day of mechanical ventilation using the first P/F ratio recorded on that day. If Pao_2_ was not collected on a given ventilator day, then P/F was imputed from the ratio of oxygen saturation as measured by pulse oximetry (Spo_2_) and Fio_2_ and the lowest imputed P/F was selected.^11^ To ensure accuracy of mechanical ventilation start and stop times, ventilator parameters, hospital discharge date, and patient status at 90 days, a 5% random sample was assessed by manual chart review (eAppendix 3 in Supplement 1).

### Outcomes

The primary outcome was in-hospital mortality within 90 days. Secondary outcomes were time to liberation from mechanical ventilation and time to discharge among those alive, and mechanical ventilation parameters in the first 7 days. Patients who discontinued mechanical ventilation but died during their hospitalization were counted as deaths and did not contribute to analyses of time on mechanical ventilation. Both time on mechanical ventilation and time to hospital discharge among those alive were administratively censored at 90 days. Physiological outcomes were static respiratory system compliance, and ventilatory ratio in the first week of mechanical ventilation. Other outcomes of interest were daily measurements of Vt, PEEP, plateau pressure, and driving pressure during the first 7 days of mechanical ventilation.

### Statistical Analysis

Our objectives were to compare differences in primary and secondary outcomes between patients with COVID-19 and non–COVID-19 pneumonia. We compared baseline characteristics between the 2 groups using χ^2^ statistics for dichotomous or categorical data or *t* tests for continuous data. We plotted patient trajectories from initiation of mechanical ventilation to time to death and time to discharge alive within 90 days. We compared cumulative incidence subdistribution functions of time to death and time to discharge alive stratified by P/F (≤150 and >150 mm Hg).

We used 2 approaches to control for confounding: (1) traditional multivariable regression models and (2) a propensity score approach to achieve exchangeability of groups with respect to observed characteristics for comparison of primary and secondary outcomes. We used propensity score full matching with propensity scores obtained using a multivariable logistic regression model of COVID-19 pneumonia as a function of age, sex, race and ethnicity, body mass index (BMI), Charlson comorbidity score, SOFA score, and P/F ratio on admission.^12,13^ Factors for the propensity score model were chosen a priori based on expert consensus by the authors and prior literature on risk factors for COVID-19 mortality. We examined covariate balance between the groups by comparing standardized mean differences before and after full matching. We also report differences in variables between groups that were not included in our a priori selection.

We then performed a series of analyses using single variable (ie, separate models with each variable) and multivariable regressions with the original and propensity score–adjusted data using doubly robust estimation to evaluate our primary and secondary outcomes.^12,13,14,15^ Weights generated by full matching were used in the propensity score–adjusted analyses. For the primary outcome of hospital mortality in the first 90 days, we used logistic regression.^16^ For time to discontinuation of mechanical ventilation and time to hospital discharge among those alive, we used proportional hazards regressions.^17^ Since death is a competing event for patients who were successfully extubated and disconnected from the mechanical ventilator or discharged from the hospital among those alive, we censored time to discontinuation of mechanical ventilation at 90 days for those who died before that period. We used sandwich clustered estimators to obtain robust standard errors to account for the full-matching weights. Doubly robust estimation was achieved by adjusting the propensity-score regression models using the same factors selected for generating the weights.^13^ For mechanical ventilation parameters, we used linear regressions.

Given potential concern for secular changes in practice, we evaluated mechanical ventilation parameters including tidal volume, PEEP and plateau pressure by calendar year using boxplots and linear regression for each ventilator parameter as a function of calendar year. We also conducted sensitivity analyses of the multivariable and propensity-adjusted models in which we limited our analyses to the subset of non-COVID-19 pneumonia patients by calendar year.

All hypothesis tests were conducted as 2-sided tests and we used a *P* value of .05 to determine statistical significance. Data processing and statistical analyses were conducted in R version 4.0.5 alias Shake and Throw (R Project for Statistical Computing). We provide statistical code used for this analysis in eAppendix 4 in Supplement 1.

## Results

### Patient Characteristics and Unadjusted Clinical Outcomes

Baseline demographics characteristics are summarized in Table 1. We identified 719 mechanically ventilated adults with COVID-19 pneumonia (mean [SD] age, 61.8 [15.3] years, 442 [61.5%] male patients; 460 patients [64.0%] belonging to a minoritized racial group and 253 [35.2%] White patients) and 1127 mechanically ventilated adults with non–COVID-19 pneumonia (mean [SD] age, 60.9 [15.8] years; 586 [52.0%] male patients; 459 patients [40.7%] belonging to a minoritized racial group and 655 [58.1%] White patients). There were no secular trends in set Vt (mean difference, 0.03 mL/kg PBW, 95% CI, −0.02 to 0.08; *P* = .21), PEEP (mean difference, 0.01 cm H_2_O; 95% CI, −0.04 to 0.06 cm H_2_O; *P* = .65), or plateau pressure (mean difference, 0.13 cm H_2_O; 95% CI, −0.01 to 0.26 cm H_2_O; *P* = .06) by calendar year between 2016 and 2019 to indicate potential changes in mechanical ventilation practices in the time before 2020 (eAppendix 5 in Supplement 1). Before matching, there were notable differences between the groups (Table 1). Specifically, patients with COVID-19 were more likely to be male, belong to a minoritized racial group, and have diabetes, a higher mean BMI, a lower SOFA score, and a lower mean P/F ratio. Patients with COVID-19 were intubated a mean 4.1 (95% CI, 3.8 to 4.4) days after admission compared with 3.4 (95% CI, 3.1 to 3.6) days for those with non–COVID-19 pneumonia (*P* < .001).

**Table 1.** Cohort Demographic Characteristics Before and After Full Propensity Score Matching

Variable	Before matching			After full matching		
	Patients, No. (%)		*P* value	Patients, %		*P* value
	With COVID-19	With non–COVID-19		With COVID-19	With non–COVID-19	
Demographics						
Age, mean (SD), y	61.8 (15.3)	60.9 (15.8)	.22	61.9 (15.7)	62.4 (15.7)	.76
Sex						
Male	442 (61.5)	586 (52.0)	<.001	61.7	69.0	.02
Female	277 (38.5)	541 (48.0)		38.3	31.0	
Body mass index, mean (SD)^a^	32.0 (9.1)	29.7 (11.6)	<.001	32.0 (15.6)	33.1 (15.6)	.61
Race						
Minoritized group	460 (64.0)	459 (40.7)	<.001	65.0	63.3	.57
White	253 (35.2)	655 (58.1)		35.0	36.7	
Hispanic ethnicity	127 (17.7)	28 (2.5)	<.001	18.1	20.5	.48
Comorbidities						
Diabetes	164 (22.8)	165 (14.6)	<.001	23.0	15.6	.02
Cardiovascular disease	63 (8.8)	141 (12.5)	.02	8.6	10.3	.36
COPD	89 (12.4)	328 (29.1)	<.001	12.3	23.6	<.001
Chronic kidney disease	227 (31.6)	369 (32.7)	.64	31.6	24.5	.03
Immunosuppression	43 (6.0)	47 (4.2)	.09	6.2	1.6	<.001
Smoker						
Current	41 (5.7)	253 (22.4)	<.001	5.7	26.2	<.001
Former	230 (32.0)	341 (30.3)	.46	32.7	26.1	.04
Charlson Comorbidity Index, mean (SD)	2.7 (2.7)	3.8 (3.1)	<.001	2.7 (2.6)	2.9 (2.6)	.55
Clinical parameters on admission, mean (SD)						
Lowest white blood cell count, cells/mm^3^	8.6 (5.3)	11.4 (11.2)	<.001	8.4 (6.9)	12.0 (7.2)	<.001
Temperature, °C						
Highest	100.3 (1.8)	100.0 (1.7)	0.002	100.3 (1.8)	99.9 (1.8)	.04
Lowest	96.9 (5.1)	96.3 (5.5)	.01	97 (4.0)	96.6 (4.2)	.15
Modes of respiratory support prior to intubation						
Flow oxygen						
Low	474 (65.9)	628 (55.7)	<.001	67.1	56.4	.02
High	391 (54.4)	157 (13.9)	<.001	55.8	14.2	<.001
NIPPV	116 (16.1)	421 (37.4)	<.001	16.4	34.3	<.001
Clinical parameters on first day of mechanical ventilation, mean (SD)						
SOFA score	15.4 (2.4)	14.5 (2.4)	<.001	15.4 (2.3)	15.2 (2.3)	.29
Pao_2_/Fio_2_	180.0 (104.7)	245.7 (142.5)	<.001	180.1 (97.3)	188.5 (98.4)	.25
Clinical outcomes						
Hospital mortality	295 (41.0)	385 (34.2)	.003	40.2	38.0	.67

Abbreviations: COPD, chronic obstructive pulmonary disease; Fio_2_, fraction of inspired oxygen; NIPPV, noninvasive positive pressure ventilation; Pao_2_, partial pressure of oxygen; SOFA, Sequential Organ Failure Assessment.

^a^ Body mass index is calculated as weight in kilograms divided by height in meters squared.

**Figure 1.**
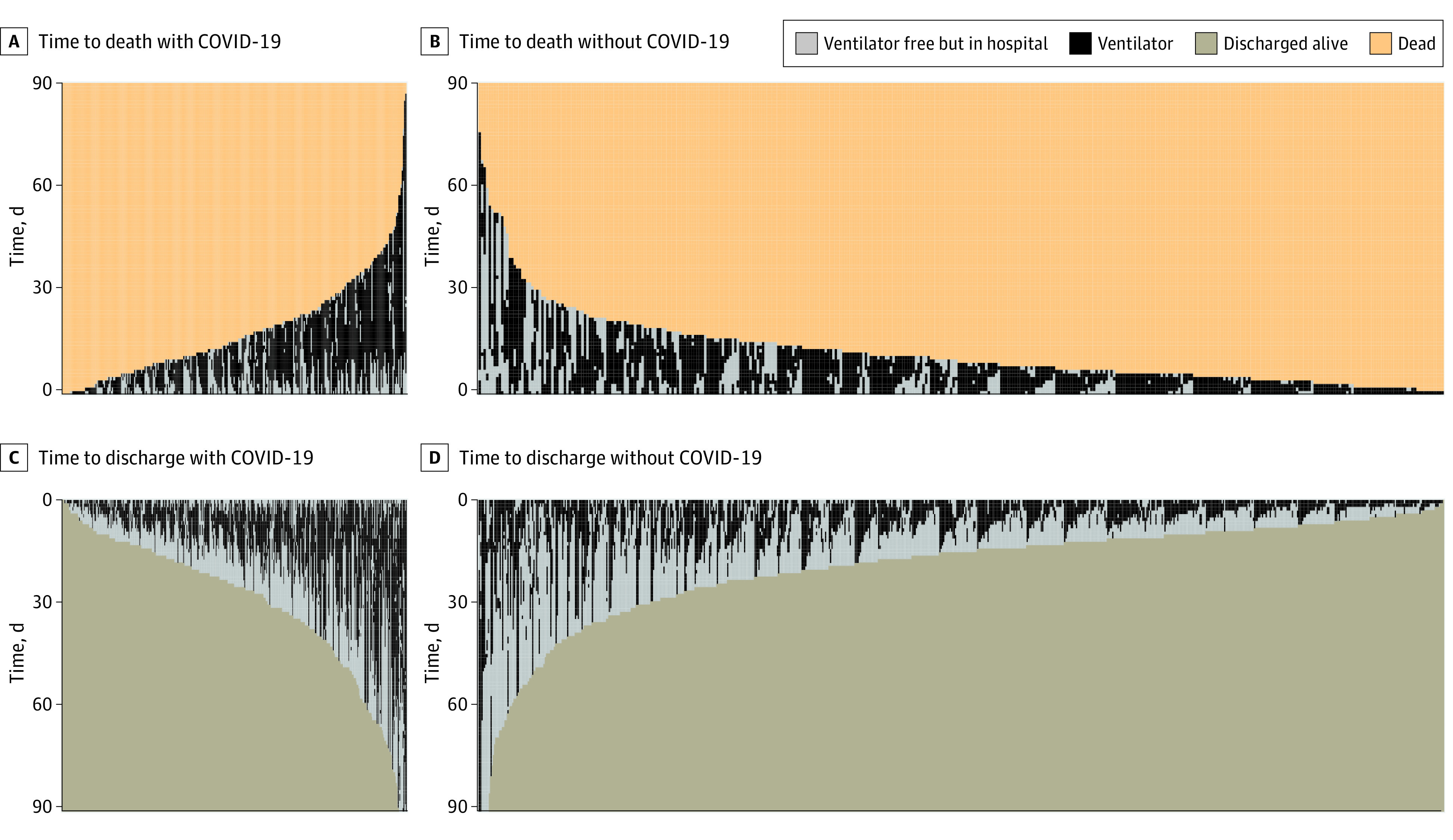
Time to Death and to Time to Discharge Alive for Mechanically Ventilated Patients With COVID-19 and Non–COVID-19 Pneumonia Before Propensity Score Adjustment This visual representation is based on unadjusted data. Each line represents an individual patient. A, Patients with COVID-19 had longer time to death compared with patients with non–COVID-19 pneumonia at 10 vs 7 days, respectively. B, Patients with COVID-19 had longer time to discharge alive compared with patients with non–COVID-19 pneumonia at 10 vs 5 days, respectively.

**Figure 2.**
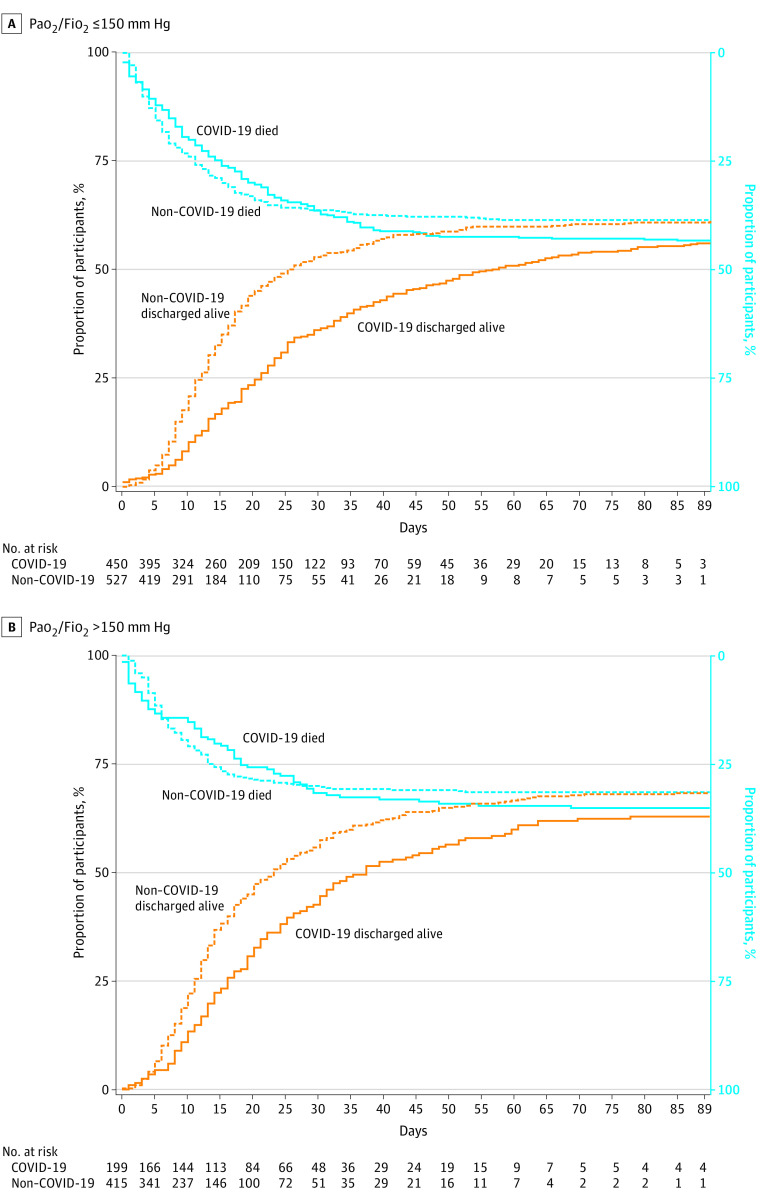
Cumulative Subdistribution Curves for Hospital Mortality and Discharge Home Alive at 90 Days Stratified by the Ratio of Partial Pressure Arterial Oxygen and Fraction of Inspired Oxygen (Pao_2_/Fio_2_) on the First Day of Mechanical Ventilation Before Propensity Score Adjustment This visual representation is based on unadjusted data. A, Hospital mortality at 90 days was not different for patients with COVID-19 compared with patients with non–COVID-19 (43% vs 39%; *P* = .57). Fewer patients with COVID-19 than non-COVID-19 were discharged alive at 90 days, but the difference was not statistically significant (56% vs 61%; *P* = .09). B, Hospital mortality at 90 days was not different for patients with COVID-19 compared with patients with non–COVID-19 (35% vs 32%; *P* = .70). Fewer patients with COVID-19 than non–COVID-19 were discharged alive at 90 days (63% vs 68%, *P* = .02).

Before matching, patients with COVID-19 pneumonia were more likely to die in the hospital, had fewer ventilator-free days, and longer hospital stays than those with non–COVID-19 pneumonia (Table 1). Patients with COVID-19 pneumonia who were discharged alive also had a median (IQR) of 10 (5-20) days of mechanical ventilation compared with 5 (3-10) days for those with non–COVID-19 pneumonia (*P* < .001). We plotted the subdistribution cumulative incidence functions of times to death and times to discharge alive stratified by COVID-19 pneumonia status in Figure 1. Patients with COVID-19 pneumonia had a longer median (IQR) time to discharge than patients with non–COVID-19 pneumonia (25 [14-39] days vs 14 [9-23] days) (Figure 1). When stratified by P/F ratio (Figure 2), the mean time to death was similar between the groups for either P/F ratio of 150 mm Hg or less (subdistribution hazard ratio [HR], 0.98; 95% CI, 0.78-1.22; *P* = .84) and P/F ratio greater than 150 mm Hg (subdistribution HR, 1.05; 95% CI, 0.84-1.30; *P* = .68), whereas the mean time to discharge alive remained longer for patients with COVID-19 pneumonia with either a P/F ratio of 150 mm Hg or less (subdistribution HR, 0.79; 95% CI, 0.63-0.99; *P* = .04) or greater than 150 mm Hg (subdistribution HR, 0.83; 95% CI, 0.72-0.96; *P* = .01).

### Results of Propensity Score Adjustment and Single Variable, Multivariable, and Propensity-Matched Analyses

We display covariate balance before and after full matching in eAppendix 6 in Supplement 1. Overall, full matching improved balance in observed characteristics. Some imbalances remained, however, for factors not selected a priori for propensity score weighting (Table 1). Patients with COVID-19 were more likely to have diabetes, chronic kidney disease, and immunosuppression and less likely to have COPD or be a current or former smoker than those with non–COVID-19 pneumonia. They were also more likely to have a lower white blood cell count at admission, more likely to be treated with high-flow nasal cannula, and less likely to be treated with noninvasive mechanical ventilation.

In single variable analysis, patients with COVID-19 pneumonia were 21% more likely to die than those with non–COVID-19 pneumonia (odds ratio, 1.21; 95% CI, 1.04-1.41) (Table 2). They were also less likely to be liberated from the ventilator or be discharged alive in the first 90 days (Table 2). In multivariable-adjusted and propensity score–adjusted analyses, however, patients with COVID-19 pneumonia were equally likely to die as those with non–COVID-19 pneumonia (odds ratio, 1.04; 95% CI, 0.81-1.35; *P* = .85) but had a lower rate of liberation from mechanical ventilation (subdistribution HR, 0.81; 95% CI, 0.65-1.00) (Table 2). Patients with COVID-19 pneumonia also had a somewhat lower rate of being discharged from the hospital alive within 90 days when compared with those with non–COVID-19 pneumonia (subdistribution HR, 0.83; 95% CI, 0.68-1.01) (Table 2); however, this was not statistically significant at the .05 level. In sensitivity analyses, we did not observe any secular trends by year in the odds ratio of hospital mortality or in the subdistribution HRs of time on mechanical ventilation or time to hospital discharge among those alive when matched sets for propensity score adjustment were limited to patients with non–COVID-19 for any single calendar year between 2016 and 2019 (eAppendix 7 in Supplement 1).

**Table 2.** Single Variable and Multivariable Models for Time to Death Within 90 Days, Time to Liberation From Mechanical Ventilation Within 90 Days, and Time to Hospital Discharge Alive Within 90 Days Before and After Propensity Score Matching^a^

Factors	OR (95% CI)			HR (95% CI)					
				Mechanical ventilation			Hospital discharge		
	Before matching		After full matching and doubly robust estimation	Before matching		After full matching and doubly robust estimation	Before matching		After full matching and doubly robust estimation
	Single variable (separate models for each factor)	Multiple variable		Single variable (separate models for each factor)	Multiple variable		Single variable (separate models for each factor)	Multiple variable	
COVID-19	1.21 (1.04-1.41)	1.15 (0.96-1.38)	1.04 (0.81-1.35)	0.72 (0.63-0.81)	0.72 (0.63-0.83)	0.81 (0.65-1.00)	0.71 (0.63-0.80)	0.74 (0.64-0.84)	0.83 (0.68-1.01)
Age, y, 1-IQR increment	1.64 (1.47-1.82)	1.60 (1.42-1.80)	NA	0.71 (0.66-0.77)	0.75 (0.70-0.81)	NA	0.72 (0.67-0.77)	0.76 (0.70-0.82)	NA
Male sex	1.17 (1.01-1.37)	1.13 (0.96-1.33)	NA	0.85 (0.76-0.96)	0.91 (0.81-1.02)	NA	0.88 (0.78-0.98)	0.93 (0.83-1.05)	NA
Body mass index, 1-IQR increment^b^	0.84 (0.77-0.92)	0.81 (0.73-0.90)	NA	1.08 (1.02-1.13)	1.09 (1.03-1.14)	NA	1.06 (1.01-1.12)	1.08 (1.02-1.13)	NA
Minoritized race	0.91 (0.79-1.06)	0.96 (0.81-1.13)	NA	0.99 (0.88-1.11)	1.01 (0.89-1.14)	NA	0.98 (0.87-1.10)	0.99 (0.88-1.12)	NA
Hispanic ethnicity	0.93 (0.71-1.22)	1.02 (0.75-1.37)	NA	0.89 (0.72-1.09)	0.91 (0.72-1.13)	NA	0.87 (0.71-1.07)	0.91 (0.72-1.13)	NA
SOFA score	0.99 (0.96-1.03)	1.00 (0.97-1.04)	NA	1.00 (0.98-1.03)	1.01 (0.99-1.04)	NA	1.00 (0.98-1.03)	1.01 (0.98-1.03)	NA
Charlson Comorbidity Index	1.08 (1.05-1.10)	1.07 (1.05-1.10)	NA	0.93 (0.92-0.95)	0.93 (0.91-0.95)	NA	0.94 (0.93-0.96)	0.94 (0.92-0.96)	NA
Pao_2_/Fio_2_, mm Hg, 1-IQR increment	0.68 (0.61-0.76)	0.64 (0.56-0.72)	NA	1.20 (1.14-1.26)	1.21 (1.14-1.27)	NA	1.22 (1.16-1.28)	1.22 (1.16-1.29)	NA


Abbreviations: FiO_2_, fraction of inspired oxygen; HR, hazard ratio; NA, not applicable; OR, odds ratio; Pao_2_, partial pressure of oxygen; SOFA, Sequential Organ Failure Assessment.

^a^ Propensity-score models used robust standard error estimates and adjust for all factors listed in the table for doubly robust estimation.

^b^ Body mass index is calculated as weight in kilograms divided by height in meters squared.

### Differences in Respiratory Physiology

**Figure 3.**
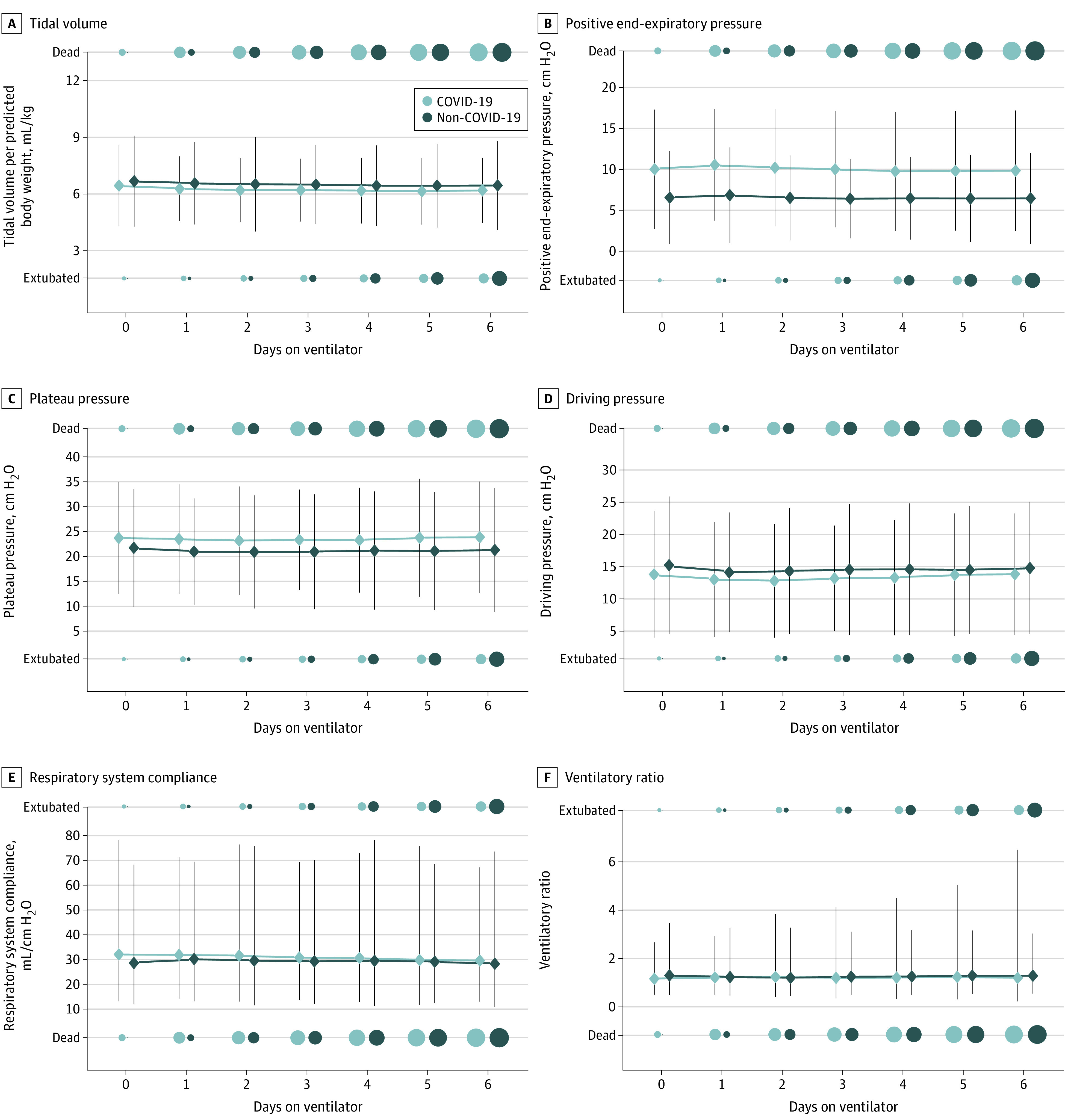
Mean Tidal Volume, Positive End-Expiratory Pressure, Plateau Pressure, Driving Pressure, Respiratory System Compliance, and Ventilatory Ratio for Patients With COVID-19 and Non–COVID-19 Pneumonia During the First Week of Mechanical Ventilation We plotted the mean (diamond) and 2 SDs (vertical line) of tidal volume per kilogram of predicted body weight (A), positive end-expiratory pressure (B), plateau pressure (C), driving pressure (D), respiratory system compliance (E), and ventilatory ratio (F) for patients with COVID-19 pneumonia and non–COVID-19 pneumonia for the first 7 days of mechanical ventilation. The number of patients who died or were extubated are represented using circles, with larger circles indicating a larger number of patients. Compared with patients with non–COVID-19, patients with COVID-19 received slightly lower tidal volumes and higher PEEP. Patients with COVID-19 also had slightly higher plateau and driving pressures, although overall these differences were small. Mean respiratory system compliance on the first day of mechanical ventilation was 32.0 mL/kg PBW/cm H_2_O for patients with severe COVID-19 and 28.4 mL/kg PBW/cm H_2_O for those with severe non–COVID-19 pneumonia (*P* < .001). Differences in mean respiratory system compliance were smaller on subsequent days. On the first day of mechanical ventilation, the mean ventilatory ratio was 1.2 for patients with severe COVID-19 pneumonia and 1.3 for those with severe pneumonia from other etiologies (*P* = .02). Differences in mean ventilatory ratio were even smaller on subsequent days.

Patients with COVID-19 were managed with slightly lower Vt (Figure 3A) and higher PEEP (Figure 3B) than those with non–COVID-19 during the first 7 days of mechanical ventilation. For example, on day 0, mean (SD) PEEP was 10.1 (3.7) cm H_2_O for patients with COVID-19 pneumonia and 6.6 (2.9) cm H_2_O for those with non–COVID-19 pneumonia (*P* < .001). Patients with COVID-19 had higher plateau pressure (Figure 3C), lower driving pressure (Figure 3D), lower respiratory system compliance (Figure 3E), and lower ventilatory ratio (Figure 3F). These differences, however, were small and unlikely to be of clinical significance. Mean (SD) respiratory system compliance on the first day of mechanical ventilation was 32.0 (1.2) mL/kg PBW/cm H_2_O for patients with COVID-19 pneumonia and 28.4 (1.2) mL/kg PBW/cm H_2_O for those with non–COVID-19 pneumonia (*P* < .001). Differences in mean respiratory system compliance were even smaller on subsequent days. After propensity score–matched analyses, we found that patients with COVID-19 had a similar respiratory system compliance compared with those with non–COVID-19 over the first 7 days of mechanical ventilation (mean difference, 1.82 mL/cm H_2_O; 95% CI, −1.53 to 5.17 mL/cm H_2_O; *P* = .28). Patients with COVID-19 pneumonia also had a slightly lower ventilatory ratio than patients with non–COVID-19 pneumonia (Figure 3F). For example, on day 0, mean (SD) ventilatory ratio was 1.2 (1.5) for patients with COVID-19 pneumonia and 1.3 (1.7) for those with severe from other etiologies (*P* = .02). Differences in mean ventilatory ratio were even smaller on subsequent days. After propensity score–matched analyses, patients with COVID-19 pneumonia had a similar mean ventilatory ratio when compared with those with non–COVID-19 pneumonia over the first 7 days of mechanical ventilation (mean difference, −0.05; 95% CI, −0.22 to 0.11; *P* = .52).

## Discussion

AHRF due to COVID-19 pneumonia has been associated with high mortality and putatively different physiologic phenotypes than other pneumonias. In this large retrospective cohort study, we compared hospital mortality, duration of mechanical ventilation, hospital length of stay, and respiratory physiology for mechanically ventilated patients with COVID-19 vs patients with non–COVID-19 pneumonia. In adjusted analyses, we found that patients with COVID-19 pneumonia had similar mortality, but longer ventilator courses. We did not identify clinically important differences in respiratory physiology. Our findings have important implications for management of patients with AHRF due to COVID-19.

Our findings add to the growing evidence that mortality for mechanically ventilated patients with COVID-19 is similar to that of patients with other pneumonias. Early in the pandemic, studies suggesting higher mortality in this population were limited by lack of appropriate control groups or failure to control for key differences between patients with and without COVID-19, leaving those comparisons at risk of bias. In contrast, studies using more rigorous analyses demonstrate that mortality for mechanically ventilated patients with COVID-19 is like that of patients with other severe pneumonias. Notably, the 40% mortally at 90 days in our cohort is like the reported mortality of 36% to 53% in other groups of mechanically ventilated patients with COVID-19 pneumonia.^4,18,19,20^ Our results are also consistent with 2 prior studies comparing mortality for mechanically ventilated patients with ARDS from COVID-19 and other etiologies.^5,6^ These studies, although with fewer patients and shorter follow-up of 28 to 60 days, also showed similar ICU or hospital mortality in adjusted analyses.

While we did not find differences in survival, patients with COVID-19 spent more time on mechanical ventilation than those with non–COVID-19 pneumonia. Consistent with our findings, COVID-19 pneumonia has been associated with longer duration of mechanical ventilation than H1N1 and other etiologies of ARDS.^5,6,21,22^ Reasons why patients with COVID-19 pneumonia have longer ventilator courses are likely multifactorial due to both pathophysiology and clinical practice. Emerging evidence suggests SARS-CoV-2 may cause persistent and slower-to-resolve alveolar inflammation that could contribute to a longer duration of mechanical ventilation.^23^ COVID-19 has also been associated with higher doses of hypnotic-sedative medications during the beginning of the pandemic, which along with constraints on family visitation, may increase risk of delirium and duration of mechanical ventilation.^24,25,26,27^

Potentially novel physiologic phenotypes in COVID-19 respiratory failure have been a topic of extensive and, at times, contentious debate. In contrast to the relative paucity of studies comparing outcomes between patients with COVID-19 and pneumonia from other causes, studies of comparative respiratory physiology abound. Our findings align with prior studies that have not identified novel physiologic phenotypes for patients with COVID-19 pneumonia. Like others, we did not find a bimodal distribution of respiratory system compliance in COVID-19 pneumonia that would suggest high and low elastance phenotypes.^28,29,30,31,32,33,34,35^ Our findings are also consistent with studies that have not identified clinically important differences in compliance between patients with COVID-19 and respiratory failure from other etiologies.^5,6,31,36^ In contrast to other studies, however, we did not find a difference in ventilatory ratio between patients with COVID-19 and non–COVID-19 pneumonias. While not defined by respiratory system compliance, emerging evidence does suggest that subphenotypes of COVID-19 respiratory failure exist. For example, patients with COVID-19 ARDS may exhibit both hypo-inflammatory and hyperinflammatory subphenotypes that have been identified in other etiologies of ARDS.^37^

### Strengths and Limitations

Our study has several strengths. First, it is one of the largest comparative studies of mortality for mechanically ventilated patients with COVID-19 and non–COVID-19 pneumonia. Second, our data include patients from both academic and community hospitals from our hospital health system, reflecting practice in multiple settings. Third, we used rigorous analytic methods to account for observed differences between patients with COVID-19 and pneumonia from other etiologies. Furthermore, longer follow-up of 90 days enabled us to better understand patient trajectories beyond the ICU.^4^

Our study also has limitations. First, we studied outcomes in a single health system, which may limit generalizability to other settings. Second, this is a retrospective study of EHR data, and despite careful measures to account for data missingness and data validation, there may be unidentified differential patterns of data errors or missingness that may introduce bias. For example, we did not have data regarding superimposed bacterial infections for patients with COVID-19, and patients with non–COVID-19 pneumonia were identified using *ICD-10* codes rather than microbiologic data. We also adjusted for a large set of observed factors, including demographic characteristics and severity scores that may explain mortality and other clinical outcomes, but we cannot exclude the possibility of unmeasured or residual confounding in our analysis. Third, we did not systematically capture differences in management, such as therapies for ARDS (eg, compliance with low tidal volume ventilation, or use of prone positioning). However, we did not find significant differences in management of mechanical ventilation parameters, including Vt or PEEP, between 2016 and 2019 and did not observe differences in mortality, time on mechanical ventilation, or time to discharge when the selection of propensity matched sets was limited to any single calendar year between 2016 and 2019. Further work is needed to examine differences in practice patterns before and during the pandemic and to determine whether this affected our findings. Fourth, we could not include patients with non–COVID-19 pneumonia between March 1, 2020, and June 30, 2021, because there were an insufficient number of them during the pandemic in our medical ICUs through many of the waves of the pandemic. Instead, we included patients with non–COVID-19 pneumonia from a recent historical period, ie, 2016 to 2019. Additionally, even though propensity score analyses aim to achieve balance in covariates between groups, remaining unmeasured confounders may still be present.

## Conclusions

In this cohort study conducted in a multiple hospital health care system, mechanically ventilated patients with severe COVID-19 pneumonia experienced similar hospital mortality but a longer duration of mechanical ventilation compared with patients with pneumonia from other etiologies. We did not find convincing evidence of different physiologic phenotypes in patients with COVID-19 pneumonia. We caution that deviating from current evidence-based practices (until there are robust data indicating why, how, and when) risks harm.

## Notes

Supplement 1. **eAppendix 1**. *ICD-10* Codes **eAppendix 2. ***ICD-10* Codes Used to Define Comorbidities **eAppendix 3. **Description of Data **eAppendix 4. **Analytical Methods and Code **eAppendix 5. **Boxplots of Tidal Volume, Positive End-Expiratory Pressure, and Plateau Pressure by Calendar Year Between 2016 and 2019 **eAppendix 6. **Love Plot Comparing Absolute Standardized Mean Differences for Key Variables Between Patients With Severe COVID-19 and Non–COVID-19 Pneumonia Before and After Full Optimal Propensity Score Matching **eAppendix 7. **Odds Ratio of Hospital Mortality and Subdistribution Hazard Ratios of Times on Mechanical Ventilation and Hospital Discharge Among Those Alive When Matched Sets for Propensity Score Adjustment Was Limited to Patients With Non–COVID-19 for Any Single Calendar Year Between 2016 and 2019

Supplement 2.
Data Sharing Statement
