# Supplementary material for: Outcomes Among Mechanically Ventilated Patients With Severe Pneumonia and Acute Hypoxemic Respiratory Failure From SARS-CoV-2 and Other Etiologies
Source: JAMA Netw Open. 2023 Jan 10;6(1):e2250401. doi: 10.1001/jamanetworkopen.2022.50401 (PMC9856712; doi:10.1001/jamanetworkopen.2022.50401)
Supplement: Supplement 1. — eAppendix 1. ICD-10 Codes eAppendix 2. ICD-10 Codes Used to Define Comorbidities eAppendix 3. Description of Data eAppendix 4. Analytical Methods and Code eAppendix 5. Boxplots of Tidal Volume, Positive End-Expiratory Pressure, and Plateau Pressure by Calendar Year Between 2016 and 2019 eAppendix 6. Love Plot Comparing Absolute Standardized Mean Differences for Key Variables Between Patients With Severe COVID-19 and Non–COVID-19 Pneumonia Before and After Full Optimal Propensity Score Matching eAppendix 7. Odds Ratio of Hospital Mortality and Subdistribution Hazard Ratios of Times on Mechanical Ventilation and Hospital Discharge Among Those Alive When Matched Sets for Propensity Score Adjustment Was Limited to Patients With Non–COVID-19 for Any Single Calendar Year Between 2016 and 2019 [file jamanetwopen-e2250401-s001.pdf]

Supplemental Online Content

Nolley EP, Sahetya SK, Hochberg CH, et al. Outcomes among mechanically ventilated patients with severe pneumonia and acute hypoxemic respiratory failure from SARS-CoV-2 and other etiologies. *JAMA Netw Open.* 2023;6(1):e2250401. doi:10.1001/jamanetworkopen.2022.50401

eAppendix 1 . *ICD-10* Codes

eAppendix 2. *ICD-10* Codes Used to Define Comorbidities

eAppendix 3. Description of Data

eAppendix 4. Analytical Methods and Code

eAppendix 5. Boxplots of Tidal Volume, Positive End-Expiratory Pressure, and Plateau Pressure by Calendar Year Between 2016 and 2019

eAppendix 6. Love Plot Comparing Absolute Standardized Mean Differences for Key Variables Between Patients With Severe COVID-19 and Non–COVID-19 Pneumonia Before and After Full Optimal Propensity Score Matching

eAppendix 7. Odds Ratio of Hospital Mortality and Subdistribution Hazard Ratios of Times on Mechanical Ventilation and Hospital Discharge Among Those Alive When Matched Sets for Propensity Score Adjustment Was Limited to Patients With Non–COVID-19 for Any Single Calendar Year Between 2016 and 2019

This supplemental material has been provided by the authors to give readers additional information about their work.

© 2023 Nolley EP et al. *JAMA Network Open.*

eAppendix 1. *ICD-10* Codes

For both COVID and non-COVID Cohorts: Initial data comprised patients having an ICD-10 PCS procedure codes of 5A1945Z (Respiratory Ventilation, 24-96 Consecutive Hours) or 5A1955Z (Respiratory Ventilation, Greater than 96 Consecutive Hours).

For the non-COVID Cohort: Patients further filtered to those with a discharge diagnosis of pneumonia that is present on admission (present on admission [POA] flag = yes).

Pneumonia ICD-10 codes for inclusion:

**Type of pneumonia**	**ICD-10 code**
Viral pneumonia	J12.89, J10.00, J12.9, J10.08, J11.08, U07.1,
	J12.0, J12.3, J09.X1, J10.01, J11.00, J12.2,
	J12.1, B97.29
Bacterial pneumonia	J15.9, J15.0, B95.3, B96.1, J13, J14, J15.5,
	J15.212, J15.211, J15.7, J15.6, J15.8,
	J15.29, J15.4, J15.1, J15.20, J15.3, A40.3,
	A37.81
Fungal pneumonia	J16.8, B37.1, J18.1
Aspiration pneumonia	J69.0
Pneumonia with unspecified etiology	J18.9, J18.0, J18.8

© 2023 Nolley EP et al. *JAMA Network Open.*

eAppendix 2. *ICD-10* Codes Used to Define Comorbidities

COPD	J44.
Asthma	J45
Chronic Heart Failure (CHF)	I50
Chronic Kidney Disease (CKD)	N18
End Stage Renal Disease (ESRD)	N18.6
Immunosuppressed	D84., Z94.
Pregnant	Z33
Current smoker	F17.21
Former smoker	Z87.891
Confusion	R41.0
Myocardial infarction (MI)	I21, I22, I25.2
Peripheral Vascular Disease	I71, I79.0, I73.9, R02, Z95.8, Z95.9
Cerebrovascular Disease (CVD)	I60, I61, I62, I63, I65, I66, G45.0, G45.1, G45.2, G45.8, G45.9, G46, I64, G45.4, I67.0, I67.1, I67.2, I67.4, I67.5, I67.6, I67.7, I67.8, I67.9, I68.1, I68.2, I68.8, I69
Dementia	F00, F01, F02, F05.1
Pulmonary diseases	J40, J41, J42, J44, J43, J45, J46, J47, J67, J44, J60, J61, J62, J63, J66, J64, J65
Connective tissue disorders	M32, M34, M33.2, M05.3, M05.8, M05.9, M06.0, M06.3, M06.9, M05.0, M05.2, M05.1, M35.3
Peptic Ulcer Disease	K25, K26, K27, K28
Liver diseases	K70.2, K70.3, K73, K71.7, K74.0, K74.2, K74.6, K74.3, K74.4, K74.5
Diabetes mellitus (DM)	E10.9, E11.9, E13.9, E14.9, E10.1, E11.1, E13.1, E14.1, E10.5, E11.5, E13.5, E14.5
Diabetes mellitus complications	E10.2, E11.2, E13.2, E14.2, E10.3, E11.3, E13.3, E14.3, E10.4, E11.4, E13.4, E14.4
Paraplegia	G81, G04.1, G82.0, G82.1, G82.2
Renal diseases	N03, N05.2, N05.3, N05.4, N05.5, N05.6, N07.2, N07.3, N07.4, N01, N18, N19, N25
Cancer	C0, C1, C2, C3, C40, C41, C43, C45, C46, C47, C48, C49, C5, C6, C70, C71, C72, C73, C74, C75, C76, C80, C81, C82, C83, C84, C85, C88.3, C88.7, C88.9, C90.0, C90.1, C91, C92, C93, C94.0, C94.1, C94.2, C94.3, C94.51, C94.7, C95, C96
Metastatic cancer	C77, C78, C79, C80
Liver diseases	K72.9, K76.6, K76.7, K72.1
HIV	B20, B21, B22, B23, B24
Leukemia	C95.9
Lymphoma	C85.90

© 2023 Nolley EP et al. *JAMA Network Open.*

Uncomplicated DM	E11.9
DM with end organ damage	E11.22
Liver cirrhosis	K74.69, K70.30, K74.60, K71.7, K74, K70.31
Multiple myeloma	C90
Hepatic failure	K91.82, K70.41, K70.40, K12.01, K72

© 2023 Nolley EP et al. *JAMA Network Open.*

eAppendix 3. Description of Data

Validation Data validation

A 5% random sample of each cohort was selected, and a manual chart review was performed to validate EHR data extraction for select data elements and outcomes. EHR extracted values were compared to those determined by manual chart review for the following data elements:

1. Mechanical ventilation start time
2. Hospital discharge date
3. Vital status (alive/dead) at hospital discharge
4. Tidal volume and positive-end expiratory pressure on mechanical ventilation day 0

Regarding mechanical ventilation, 100% of patients were correctly identified as receiving mechanical ventilation. The start time of mechanical ventilation was within 12-hours of the start time determined by manual chart in 99% of the validation sample, and when discrepancies arose, they stemmed from delay in charting ventilator mode after intubation.

Hospital discharge date and vital status at discharge was 100% accurate when comparing EHR-extracted and manual chart review values.

Tidal volume and PEEP were also 100% accurate when compared to manual chart review.

© 2023 Nolley EP et al. *JAMA Network Open.*

eAppendix 4. Analytical Methods and Code

Data manipulation and dataset creation

```
### SQL Server info
server = # Provide server name
db_name = # Provide database name
pwd = # Provide JHED password
stopifnot(nzchar(pwd))
JHED = # Provide JHED ID
stopifnot(nzchar(pwd))

connectionString <- # Develop a connection string with Driver, TDS_version, Server, Port,
    Database name, Uid, JHED & password

con <- odbcDriverConnect(connection=connectionString)
tabs <- sqlTables(con)
tabs = tabs[tabs$TABLE_SCHEM=="dbo",]

df.list = tabs$TABLE_NAME[tabs$TABLE_SCHEM=="dbo"]

### Rename datasets
df.list[df.list == "covid_pmcoe_covid_positive"] = "pmcoe"
df.list[df.list == "crown_nolley_epic_vitals_control"] = "vitals_ctrl"
df.list[df.list == "curated_Adult_RTAssessment"] = "rta"
df.list[df.list == "curated_BMI"] = "bmi"
df.list[df.list == "curated_elixhauser_comorbidities"] = "comorb"
df.list[df.list == "Curated_IPEvents"] = "events"
df.list[df.list == "curated_IPVitals"] = "vitals"
df.list[df.list == "curated_Labs_backup"] = "labs_backup"
df.list[df.list == "curated_PositivePatients"] = "positive"
df.list[df.list == "derived_encounter_dx"] = "encounter"
df.list[df.list == "derived_encounter_dx_control"] = "encounter_ctrl"
df.list[df.list == "derived_epic_patient"] = "epic"
df.list[df.list == "derived_epic_patient_control"] = "epic_ctrl"
df.list[df.list == "derived_epic_vitals"] = "der_vitals"
df.list[df.list == "derived_epic_vitals_control"] = "der_vitals_ctrl"
df.list[df.list == "derived_flowsheet_data"] = "flowsheet"
df.list[df.list == "derived_flowsheet_data_control"] = "flowsheet_ctrl"
df.list[df.list == "derived_hosp_billing_dx"] = "billingdx"
df.list[df.list == "derived_hosp_billing_dx_control"] = "billingdx_ctrl"
df.list[df.list == "derived_infections"] = "infection"
df.list[df.list == "derived_infections_control"] = "infection_ctrl"
df.list[df.list == "derived_inpatient_encounters"] = "inpatient_encounters"
df.list[df.list == "derived_inpatient_encounters_control"] = "inpatient_encounters_ctrl"
df.list[df.list == "derived_lab_results"] = "lab"
```

© 2023 Nolley EP et al. *JAMA Network Open.*

```r
df.list[df.list == "derived_lab_results_control"] = "lab_ctrl"
df.list[df.list == "derived_lda_data_control"] = "lda"
df.list[df.list == "derived_med_admin"] = "med"
df.list[df.list == "derived_med_admin_control"] = "med_ctrl"
df.list[df.list == "derived_medical_hx_summary"] = "hx"
df.list[df.list == "derived_medical_hx_summary_control"] = "hx_ctrl"
df.list[df.list == "derived_problem_list"] = "problem"
df.list[df.list == "derived_problem_list_control"] = "problem_ctrl"
df.list[df.list == "dervied_encounter_dx"] = "encounterdx"
df.list

### Download the datasets from SQL database to Crunchr
for(i in 1:length(df.list)){
  print(paste0("write.csv(df.",df.list[i], dir,"/df.",df.list[i],".csv)"))

  data = assign(paste0("df.",df.list[i]), sqlFetch(con, tabs[i,3]))
  write.csv(data, file = paste0(dir, "/df.", df.list[i],".csv"))
}

##############################################
###   Load the datasets for analysis    ###
##############################################
data.list = list.files(dir,pattern="*.csv")
for(i in 1:length(data.list)) assign(str_remove(data.list[i], ".csv"),
 ↪  fread(data.list[i]))

####################
###    Functions    ###
####################
### Assign disease name from ICD-10 codes
fun.morb = function(datain, icd10, disease){
  dataout = datain %>%
    dplyr::rename(id = osler_id) %>%
    dplyr::filter(grepl(icd10, icd10_code)) %>%
    dplyr::filter(!is.na(present_on_admission) & present_on_admission != "No") %>%
    dplyr::select(id, icd10_code, dx_name)

  dataout$dx_name <- ifelse(grepl(icd10, dataout$icd10_code), 1, dataout$dx_name)
  dataout$icd10_code = NULL
  colnames(dataout) = c("id", disease)
  dataout
}


### Extract minimum and maximum values within 24 hours of admission
fun.max24 = function(datain, dataadmit, oldvar, newvar="x", wide, meas, maxtime){
  if(wide == 1){datain = full_join(datain , dataadmit, by = "id")}
  if(wide == 0){
    datain = datain %>% dplyr::filter(meas_name %in% meas)
    datain = full_join(datain , dataadmit, by = "id")
  }

  datain$recorded_time = substr(datain$recorded_time, 1, 16)
```

© 2023 Nolley EP et al. *JAMA Network Open.*

```
  datain$hospdate = substr(datain$hospdate, 1, 16)

  dataout  =  datain  %>%
    dplyr::mutate(timegap = ymd_hm(recorded_time) - ymd_hm(hospdate)) %>%
    dplyr::filter(timegap <= maxtime & timegap>=0) %>%
    dplyr::group_by(id) %>%
    dplyr::summarise(min  =  min(as.numeric(eval(parse(text=oldvar))),  na.rm  =  TRUE),
                     max  =  max(as.numeric(eval(parse(text=oldvar))),  na.rm  =  TRUE))  %>%
    dplyr::select(id,  min,  max)
  colnames(dataout) = c("id", paste0("l_",newvar), paste0("h_",newvar))
  dataout
}

### Extract minimum and maximum values from a 72 hours window period (48 hours before
↪ admission to 24 hours after admission)
fun.48_24 = function(datain, dataadmit, oldvar, newvar="x", meas, mintime, maxtime){

  datain = datain %>% dplyr::filter(meas_name %in% meas)
  datain  =  full_join(datain , dataadmit,  by  =  "id")

  datain$recorded_time  =  substr(datain$recorded_time,  1,  16)
  datain$hospdate = substr(datain$hospdate, 1, 16)

  dataout  =  datain  %>%
    dplyr::mutate(timegap = ymd_hm(recorded_time) - ymd_hm(hospdate)) %>%
    dplyr::filter(timegap >= mintime & timegap <= maxtime) %>%
    dplyr::group_by(id) %>%
    dplyr::summarise(min  =  min(as.numeric(eval(parse(text=oldvar))),  na.rm  =  TRUE),
                     max  =  max(as.numeric(eval(parse(text=oldvar))),  na.rm  =  TRUE))  %>%
    dplyr::select(id,  min,  max)
  colnames(dataout) = c("id", paste0("l_",newvar), paste0("h_",newvar))
  dataout
}

### Extract mean value from a 72 hours window period (48 hours before admission to 24
↪ hours after admission)
fun.48_24.mean = function(datain, dataadmit, oldvar, newvar="x", meas, mintime, maxtime){

  datain = datain %>% dplyr::filter(meas_name %in% meas)
  datain  =  full_join(datain , dataadmit,  by  =  "id")

  datain$recorded_time  =  substr(datain$recorded_time,  1,  16)
  datain$hospdate = substr(datain$hospdate, 1, 16)

  dataout  =  datain  %>%
    dplyr::mutate(timegap = ymd_hm(recorded_time) - ymd_hm(hospdate)) %>%
    dplyr::filter(timegap >= mintime & timegap <= maxtime) %>%
    dplyr::group_by(id) %>%
    dplyr::summarise(mean  =  mean(as.numeric(eval(parse(text=oldvar))),  na.rm  =  TRUE))  %>%
    dplyr::select(id,   mean)
  colnames(dataout) = c("id", paste0("mean_",newvar))
  dataout
}
```

© 2023 Nolley EP et al. *JAMA Network Open.*

```
### Extract first measurement of a particular date (admission date/ first day on
→ mechanical ventilation)
fun.firstmeas = function(datain, dataadmit, meas, varname, after){
  datain = full_join(datain, dataadmit, by="id")
  dataout = datain %>%
    dplyr::filter(meas_name == meas) %>%
    dplyr::mutate(timegap = (ymd_hms(recorded_time) - ymd_hms(hospdate)))
if(after==1){
  dataout = dataout %>%
    dplyr::filter(timegap>=0) %>%
    dplyr::arrange(id, recorded_time) %>%
    dplyr::select(id, meas_value) %>%
    dplyr::distinct(id, .keep_all = TRUE)
}
  if(after==0){
    dataout = dataout %>%
      dplyr::arrange(id, recorded_time) %>%
      dplyr::select(id, meas_value) %>%
      dplyr::distinct(id, .keep_all = TRUE)
  }
  colnames(dataout) = c("id", varname)
  dataout
}


### Extract data from 72 hours prior to admission (used to extract weight only)
fun.firstmeas.before72 = function(datain,dataadmit, meas, varname, tg){
  datain = full_join(datain, dataadmit, by="id")
  dataout = datain %>%
    dplyr::filter(meas_name == meas) %>%
    dplyr::mutate(timegap = (ymd_hms(recorded_time) - ymd_hms(hospdate)))

    dataout = dataout %>%
      dplyr::filter(timegap>= (-259200)) %>%
      dplyr::arrange(id, recorded_time) %>%
      dplyr::select(id, meas_value) %>%
      dplyr::distinct(id, .keep_all = TRUE)

  colnames(dataout) = c("id", varname)
  dataout
}


### Extract the date when any procedure was performed for the first time
fun.firstdate = function(datain, dataadmit, meas, newvar, recorded_time , wide){
  datain = full_join(datain, dataadmit, by = "id")
  dataout = datain %>%
    dplyr::filter(grepl(meas, meas_name))
  if(recorded_time == 1){
    dataout = dataout %>%
      dplyr::mutate(timegap = (as.Date(recorded_time) - as.Date(hospdate))) %>%
```

© 2023 Nolley EP et al. *JAMA Network Open*.

```
      dplyr::mutate(date = as.Date(recorded_time))
  }

  if(recorded_time == 0){
    dataout = dataout %>%
      dplyr::mutate(timegap = (as.Date(meas_value) - as.Date(meas_value))) %>%
      dplyr::mutate(date = as.Date(meas_value))
  }
  dataout = dataout %>%
    dplyr::distinct(id, date, .keep_all = TRUE) %>%
    dplyr::filter(timegap>=0)   %>%
    dplyr::arrange(id, date) %>%
    dplyr::select(id, date)

  if(wide == 0){
    dataout = dataout %>%
      dplyr::distinct(id, .keep_all = TRUE)
    colnames(dataout) = c("id", newvar)
  }
  if(wide == 1){
    dataout = dataout %>%
      dplyr::group_by(id) %>%
      dplyr::mutate(order = 1:n()) %>%
      spread(order, date)
  }
  dataout
}

### Calculate mean arterial pressure
fun.map = function(sbp, dbp){
  output = (sbp + (2*dbp))/3
  output
}


### Generate table reporting data missingness
fun.miss = function(datain){
  xx = NULL
  for(i in colnames(datain)){
    names = i
    miss_case = sum(is.na(datain[[i]][datain$group == "COVID19"]))
    miss_ctrl = sum(is.na(datain[[i]][datain$group == "Non-COVID19"]))
    miss_perc_case = (miss_case/dim(datain[datain$group == "COVID19",])[1])*100
    miss_perc_ctrl = (miss_ctrl/dim(datain[datain$group == "Non-COVID19",])[1])*100
    yy = cbind(names, miss_case, miss_perc_case, miss_ctrl, miss_perc_ctrl)
    xx = rbind(xx, yy)
  }
  xx = as.data.frame(xx)
  xx$miss_perc_case = paste0(xx$miss_case, " (", sprintf("%3.1f",
 ↪as.numeric(as.character(xx$miss_perc_case))), "%)")
  xx$miss_perc_ctrl = paste0(xx$miss_ctrl, " (", sprintf("%3.1f",
 ↪as.numeric(as.character(xx$miss_perc_ctrl))),   "%)")
  xx$miss_case = NULL; xx$miss_ctrl = NULL
```

© 2023 Nolley EP et al. *JAMA Network Open.*

```
  colnames(xx) = c("","COVID19", "Non-COVID19")
  xx
}

### Impute PaO2 from SpO2
fun.pao2.imp = function(spo2){
  A = (11700/(1/spo2 - 1))
  B = (((50^3) + ((11700/(1/spo2 - 1))^2))^(0.5))
  pao2_imp = ((B + A)^(1/3)) + ((B - A)^(1/3))
  return(pao2_imp)
}

### Calculate time period from admission to any perticular event/intervention (eg: low
↪ flow oxygen, high flow oxygen etc.)
fun.admission_to_mv = function(datain = df.flowsheet_1,
                               data = df1,
                               meas = c("R OXYGEN FLOW RATE", "*OLD* R JHM IP PT NEW
                                ↪ OXYGEN FLOW RATE"),
                               varname = "o2",
                               dataadmit = df.admit1,
                               datamv0 = df.mv1){
  datamv0 = datamv0 %>% dplyr::rename(mvdate0 = hospdate)
  dataout = datain %>%
    dplyr::filter(meas_name %in% meas) %>%
    dplyr::left_join(dataadmit, by = "id") %>%
    dplyr::left_join(datamv0, by = "id") %>%
    dplyr::filter(recorded_time >= hospdate & recorded_time <= mvdate0) %>%
    dplyr::arrange(id, recorded_time) %>%
    dplyr::filter(!is.na(meas_value)) %>%
    dplyr::distinct(id, .keep_all = TRUE) %>%
    dplyr::select(id, meas_value)

  colnames(dataout) = c("id", varname)
  data = left_join(data, dataout, by = c("id"))
  return(data)
}

### Extract mechanical ventilation data

fun.vent = function(datain, dataadmit){
meas_list = c("R JHM IP RT VENTILATOR MODES", "R ETT TUBE SIZE", "R ETT TYPE", "R
↪ SURGICAL AIRWAY CUFF STATUS","R JHM IP RT EVENT")
varlist = c("vent_mode", "ett_size", "ett_type", "cuff_stat", "intube_stat")
timelist = c("vent_time", "ettsize_time", "etttype_time", "cuffstat_time", "intube_time")

for(i in 1:5){
  ddd = datain %>%
    dplyr::filter(meas_name == meas_list[i]) %>%
    dplyr::mutate(date = as.Date(recorded_time)) %>%
    dplyr::select(-meas_name, -meas_template_id)
  colnames(ddd) = c("id", varlist[i], timelist[i], "date")
  assign(paste0("d",i), ddd)
}
```

© 2023 Nolley EP et al. *JAMA Network Open.*

```
d = list(d1, d2, d3, d4) %>% reduce(full_join, by = c("id", "date"))
d = d %>%
  filter((cuff_stat != "Cuffless" & cuff_stat != "Deflated") | is.na(cuff_stat)) %>%
  filter(vent_mode %in% c("PRVC", "SIMV Pressure", "SIMV PRVC", "SIMV Vol", "VC", "VS",
  ↪ "Bi-level/Bi-vent") |
           (vent_mode == "PC" & (cuff_stat == "Inflated" | !is.na(ett_size)))) %>%
  left_join(dataadmit, by = "id") %>%
  filter(vent_time > hospdate) %>%
  dplyr::select(id, vent_time) %>%
  dplyr::rename(hospdate = vent_time) %>%
  arrange(id, hospdate) %>%
  distinct(id, .keep_all = TRUE)
return(d)
}

### Extract first value of any particular measurement on each day
fun.firstmeas.day = function(datain, data, meas, varname, oldvar, admit, min, max){
  datain = datain
  dataout = left_join(datain, admit, by="id") %>%
    dplyr::filter(meas_name %in% meas)

  if("ord_value" %in% names(datain) == "TRUE"){
    dataout = dataout %>% dplyr::rename(meas_value = ord_value)}
  dataout = dataout %>%
    dplyr::filter(as.numeric(meas_value) >= min & as.numeric(meas_value) <= max) %>%
    dplyr::mutate(id = paste0(id, "_", as.character(as.Date(hospdate)))) %>%
    arrange(id, recorded_time) %>%
    mutate(mvdate = as.Date(recorded_time)) %>%
    dplyr::distinct(id, mvdate, .keep_all = TRUE) %>%
    dplyr::select(id, oldvar, mvdate)

  colnames(dataout) = c("id", varname, "mvdate")
  data = left_join(data, dataout, by = c("id", "mvdate"))
  return(data)
}


### Extract lowest, highest and mean value on each day
fun.lhm = function(datain, meas, oldvar, name, data, admit){
  dataout = left_join(datain, admit, by="id") %>%
    dplyr::filter(meas_name == meas) %>%
    dplyr::mutate(id = paste0(id, "_", as.character(as.Date(hospdate)))) %>%
    dplyr::mutate(mvdate = as.Date(recorded_time)) %>%
    dplyr::group_by(id, mvdate) %>%
    dplyr::summarise(low = min(as.numeric(eval(parse(text=oldvar))), na.rm = TRUE),
                     high = max(as.numeric(eval(parse(text=oldvar))), na.rm = TRUE),
                     mean = mean(as.numeric(eval(parse(text=oldvar))), na.rm = TRUE))

  colnames(dataout) = c("id", "mvdate", paste0(name,"_low"), paste0(name,"_high"),
  ↪ paste0(name,"_mean"))
  data = full_join(data, dataout, by = c("id", "mvdate"))
  return(data)
}
```

© 2023 Nolley EP et al. *JAMA Network Open.*

```
### Filter if the patient got any particular drug on day or not
fun.today = function(datain, var, drug, rt, varname, data, ds, admit){
  dataout = full_join(datain, admit, by="id") %>%
    dplyr::filter(grepl(drug,  eval(parse(text=var))))  %>%
    dplyr::filter(dose >= ds) %>%
    dplyr::filter(route %in% rt) %>%
    dplyr::mutate(mvdate = as.Date(recorded_time)) %>%
    dplyr::distinct(id, mvdate, .keep_all = TRUE) %>%
    dplyr::mutate(yes = 1) %>%
    dplyr::mutate(id = paste0(id, "_", as.character(as.Date(hospdate)))) %>%
    dplyr::select(id, mvdate, yes)

  colnames(dataout) = c("id", "mvdate", varname)
  data = full_join(data, dataout, by = c("id", "mvdate"))
  return(data)
}

### Make the IDs unique
fun.uniqueid = function(datain, datamerge){
  datain = left_join(datain, datamerge, by = "id")
  datain$id = paste0(datain$id, "_", as.character(as.Date(datain $hospdate)))
  return(datain)
}


### Extract mean value within the window period of 72 hours (48 hours before MV date to
↪ 24 hours after MV)
fun.48_24.mean.mv0 = function(datain, data, dataadmit, datamv0, oldvar, newvar="x", meas,
↪ mintime, maxtime){
  datamv0 = datamv0 %>% dplyr::rename(mvdate0 = mvdate)

  dataout = datain %>%
    dplyr::filter(grepl(meas, meas_name)) %>%
    left_join(dataadmit, by="id") %>%
    left_join(datamv0, by = "id") %>%
    dplyr::mutate(id = paste0(id, "_", as.character(as.Date(hospdate)))) %>%
    dplyr::mutate(timegap = ymd_hms(recorded_time) - ymd_hms(mvdate0))

dataout = dataout %>%
    dplyr::filter(timegap >= mintime & timegap <= maxtime) %>%
    dplyr::mutate(mvdate = as.Date(recorded_time)) %>%
    dplyr::group_by(id) %>%
    dplyr::summarise(mean = mean(as.numeric(eval(parse(text=oldvar))), na.rm = TRUE)) %>%
    dplyr::select(id,  mean)

  colnames(dataout) = c("id", newvar)
  data = left_join(data, dataout, by = "id")
  return(data)
}


### Extract mechanical ventilation data on each day
```

© 2023 Nolley EP et al. *JAMA Network Open.*

```
fun.vent.long = function(datain, idmatch, dataadmit){
  meas_list = c("R JHM IP RT VENTILATOR MODES", "R ETT TUBE SIZE", "R ETT TYPE", "R
  ↪  SURGICAL AIRWAY CUFF STATUS","R JHM IP RT EVENT")
  varlist = c("vent_mode", "ett_size", "ett_type", "cuff_stat", "intube_stat")
  timelist = c("vent_time", "ettsize_time", "etttype_time", "cuffstat_time",
  ↪  "intube_time")

  for(i in 1:5){
    ddd = datain %>%
      dplyr::filter(meas_name == meas_list[i]) %>%
      dplyr::mutate(date = as.Date(recorded_time)) %>%
      dplyr::select(-meas_name, -meas_template_id)
    colnames(ddd) = c("id", varlist[i], timelist[i], "date")
    assign(paste0("d",i),   ddd)
  }
 d = list(d1, d2, d3, d4) %>% reduce(full_join, by = c("id", "date"))
 d = d %>%
    filter((cuff_stat != "Cuffless" & cuff_stat != "Deflated") | is.na(cuff_stat)) %>%
    filter(vent_mode %in% c("PRVC", "SIMV Pressure", "SIMV PRVC", "SIMV Vol", "VC", "VS",
 ↪  "Bi-level/Bi-vent", "PS") |
                (vent_mode == "PC" & (cuff_stat == "Inflated" | !is.na(ett_size)))) %>%
    filter(id %in% idmatch) %>%
    left_join(dataadmit, by = "id") %>%
    filter(vent_time >= hospdate) %>%
    dplyr::mutate(id = paste0(id, "_", as.character(as.Date(hospdate)))) %>%
    mutate(mvdate = as.Date(vent_time),
           date = as.Date(vent_time),
           vent_time = as.POSIXct(vent_time, format = "%Y-%m-%d %H:%M:%S"),
           time = format(vent_time, format = "%H:%M:%S")) %>%
    dplyr::select(id, mvdate, date, time) %>%
   distinct()
  return(d)
}

### Extract Blood pressure data
fun.bp = function(datain, dataadmit){
  dataout = left_join(datain, dataadmit, by="id") %>%
    dplyr::mutate(mvdate = as.Date(recorded_time)) %>%
    dplyr::mutate(id = paste0(id, "_", as.character(as.Date(hospdate)))) %>%
    dplyr::arrange(id, recorded_time) %>%
    dplyr::distinct(id, mvdate, .keep_all = TRUE) %>%
    dplyr::select(-recorded_time)
return(dataout)
}

###   Generate list of IDs who had tracheostomy on admission
fun.trach = function(datain, dataadmit){
  dataout = datain %>%
    filter(meas_name %in% c("OLD R AIRWAY SUCTION TYPE", "R SURGICAL AIRWAY TYPE", "R JHM
 ↪  IP RT PATIENT STATUS",
                          "R SURGICAL AIRWAY INNER CANNULA CARE", "R RESP CPT SUCTION",
                          ↪
                          "HCGH ED R ASSISTIVE NEEDS", "R SURGICAL AIRWAY SITE CARE"))
                          ↪  %>%
```

© 2023 Nolley EP et al. *JAMA Network Open.*

```r
    right_join(dataadmit, by = "id") %>%
    filter(as.Date(hospdate) == as.Date(recorded_time)) %>%
    filter(grepl("trach|Trach", meas_value)) %>%
    dplyr::select(id) %>%
    distinct()
  return(dataout)
}
df.trach1 = fun.trach(df.flowsheet_1, df.admit1)
df.trach2 = fun.trach(df.flowsheet_2, df.admit2)

#################################################################
###   Gender, Ethnicity, Race, Death date, Birth date, group   ###
#################################################################
df.epic_1 = df.epic %>%
  dplyr::select(OSLER_ID, Pat_gender, Ethnic_group, first_race, Death_date , Birth_Date,
↪   EMRN) %>%
  dplyr::rename(id  =  OSLER_ID,
                ma0fe1 = Pat_gender,
                ethnicity = Ethnic_group,
                date_death = Death_date,
                birth_date = Birth_Date,
                race = first_race,
                emrn = EMRN)

df.epic_2 = df.epic_ctrl %>%
  dplyr::select(osler_id, gender, ethnic_group, first_race, death_date, birth_date, emrn)
↪   %>%
  dplyr::rename(id = osler_id,
                ethnicity = ethnic_group,
                ma0fe1 = gender,
                race = first_race,
                date_death = death_date) %>%
  dplyr::distinct(id, .keep_all = TRUE)

### Case-control variable
df.epic_1$group = "COVID19"
df.epic_2$group = "Non-COVID19"

#####################################
###    Admission and discharge time    ###
#####################################
df.events_1 = df.events %>%
  dplyr::select(OSLER_ID, ADMIT_TIME, FINAL_HOSP_DISCH_TIME, FINAL_DISCH_DISP_NAME) %>%
  dplyr::rename(id  =  OSLER_ID,
                hospdate = ADMIT_TIME,
                date_discharge = FINAL_HOSP_DISCH_TIME,
                ltac = FINAL_DISCH_DISP_NAME)

df.inpatient_encounters_2 = df.inpatient_encounters_ctrl %>%
  dplyr::select(osler_id, hosp_admsn_time, hosp_disch_time, disc_disp) %>%
  dplyr::rename(id = osler_id,
                hospdate = hosp_admsn_time,
                date_discharge = hosp_disch_time,
```

© 2023 Nolley EP et al. *JAMA Network Open.*

```
                    ltac = disc_disp) %>%
  dplyr::arrange(id, desc(hospdate)) %>%
  dplyr::distinct(id, .keep_all = TRUE)

df1  = merge(df.epic_1, df.events_1, by="id", all  =TRUE)
df2  = merge(df.epic_2, df.inpatient_encounters_2, by="id", all  = TRUE)

df.admit1 = df1 %>% dplyr::select(id, hospdate)
df.admit2 = df2 %>% dplyr::select(id, hospdate)

#############################
###    Creating datasets     ###
#############################
df.flowsheet_1   =  df.flowsheet  %>%
  dplyr::select(osler_id, meas_name, meas_value, recorded_time, meas_template_id) %>%
  dplyr::rename(id  =  osler_id)  %>%
  dplyr::filter(!is.na(meas_value))

df.flowsheet_2   =  df.flowsheet_ctrl  %>%
  dplyr::select(osler_id, meas_name, meas_value, recorded_time, meas_template_id) %>%
  dplyr::rename(id  =  osler_id)  %>%
  dplyr::filter(!is.na(meas_value))

df.vitals_1   =  df.der_vitals  %>%
  dplyr::select(osler_id, meas_name, meas_value, recorded_time, meas_template_id) %>%
  dplyr::rename(id  =  osler_id)  %>%
  dplyr::filter(!is.na(meas_value))

df.vitals_2   =  df.der_vitals_ctrl  %>%
  dplyr::select(osler_id, meas_name, meas_value, recorded_time, meas_template_id) %>%
  dplyr::rename(id  =  osler_id)     %>%
  dplyr::filter(!is.na(meas_value))

df.bp1  = df.vitals_1  %>%
  dplyr::filter(meas_name  ==  "BLOOD  PRESSURE")  %>%
  dplyr::select(id, meas_value, recorded_time)  %>%
  separate(meas_value, c("sbp", "dbp"))

df.bp2  = df.vitals_2  %>%
  dplyr::filter(meas_name  ==  "BLOOD  PRESSURE")  %>%
  dplyr::select(id, meas_value, recorded_time)  %>%
  separate(meas_value, c("sbp", "dbp"))

df.bpart1  =  df.vitals_1  %>%
  dplyr::filter(meas_name == "R ARTERIAL  LINE  BLOOD  PRESSURE") %>%
  dplyr::select(id, meas_value, recorded_time) %>%
  separate(meas_value, c("sbp", "dbp"))

df.bpart2   =   df.flowsheet_2   %>%
  dplyr::filter(meas_name == "R ARTERIAL  LINE  BLOOD  PRESSURE") %>%
  dplyr::select(id, meas_value, recorded_time) %>%
  separate(meas_value, c("sbp", "dbp"))
```

© 2023 Nolley EP et al. *JAMA Network Open.*

```
df.lab_1  =  df.lab  %>%
  dplyr::rename(recorded_time = specimen_taken_time,
                 meas_name = component_base_name,
                id  =  osler_id)  %>%
 dplyr::filter(!is.na(ord_value))

df.lab_2  =  df.lab_ctrl  %>%
  dplyr::rename(recorded_time = specimen_taken_time,
                 meas_name = component_base_name,
                id  =  osler_id)  %>%
 dplyr::filter(!is.na(ord_value))

df.lab_2$meas_name = as.character(df.lab_2$meas_name)

df.rta_1 = df.rta %>%
  dplyr::select(osler_id, recorded_time, meas_name, meas_disp_name, meas_value,
 ↪meas_comment) %>%
  dplyr::rename(id = osler_id) %>%
  dplyr::filter(!is.na(meas_value))

df.med_1 = df.med %>% dplyr::rename(id = osler_id, recorded_time = TakenTime, name =
 ↪  MedName, class = PharmClass,
                                  dose  =  Dose,  route  =  MAR_Route)
df.med_2 = df.med_ctrl %>% dplyr::rename(id = osler_id, recorded_time = taken_time, name
 ↪  = medication_name,
                                        class = pharm_classname, dose = sig)

df.mv1 = fun.vent(df.flowsheet_1, df.admit1)
df.mv2  =  fun.vent(df.flowsheet_2,  df.admit2)

######################################
###    Extract  Height  and  Weight    ###
######################################
df.bmi1  =  fun.firstmeas(df.vitals_1,  df.admit1,  "R  BMI",  "bmi",  after  =  0);  df1  =
 ↪  full_join(df1, df.bmi1, by="id")
df.bmi2  =  fun.firstmeas(df.vitals_2,  df.admit2,  "R  BMI",  "bmi",  after  =  0);  df2  =
 ↪  full_join(df2, df.bmi2, by="id")

df.height1  =  fun.firstmeas(df.vitals_1,  df.admit1,  "HEIGHT",  "height",  after  =  0);  df1  =
 ↪  full_join(df1,  df.height1,  by="id")
df.height2  =  fun.firstmeas(df.vitals_2,  df.admit2,  "HEIGHT",  "height",  after  =  0);  df2  =
 ↪  full_join(df2,  df.height2,  by="id")

df.weight1 = fun.firstmeas(df.vitals_1, df.admit1, "WEIGHT/SCALE", "weight", after = 0);
 ↪  df1  =  full_join(df1,  df.weight1,  by="id")
df.weight2 = fun.firstmeas(df.vitals_2, df.admit2, "WEIGHT/SCALE", "weight", after = 0);
 ↪  df2  =  full_join(df2,  df.weight2,  by="id")

df1$height = as.numeric(df1$height)*2.54
df2$height = as.numeric(df2$height)*2.54

df1$weight = as.numeric(as.character(df1$weight))*0.0283495
df2$weight = as.numeric(as.character(df2$weight))*0.0283495
```

© 2023 Nolley EP et al. *JAMA Network Open.*

```
df1$bmi_calc = df1$weight/(df1$height/100)^2
df2$bmi_calc = df2$weight/(df2$height/100)^2

df1$bmi = ifelse(is.na(df1$bmi), as.numeric(df1$bmi_calc), df1$bmi)
df2$bmi  =  ifelse(is.na(df2$bmi),  as.numeric(df2$bmi_calc),  df2$bmi)

####################################################
###     SBP, DBP, Temp, Resp, GCS on Admission     ###
####################################################
df.sbp1 = fun.max24(df.bp1, df.admit1, "sbp", "sbp_admit", wide = 1, "sbp", maxtime =
↪  86400); df1 = full_join(df1, df.sbp1, by = "id")
df.dbp1 = fun.max24(df.bp1, df.admit1, "dbp", "dbp_admit", wide = 1, "dbp", maxtime =
↪  86400); df1 = full_join(df1, df.dbp1, by = "id")

df.sbp1 = fun.max24(df.bpart1, df.admit1, "sbp", "sbpart_admit", wide = 1, "sbp", maxtime
↪  = 86400); df1 = full_join(df1, df.sbp1, by = "id")
df.dbp1 = fun.max24(df.bpart1, df.admit1, "dbp", "dbpart_admit", wide = 1, "dbp", maxtime
↪  = 86400); df1 = full_join(df1, df.dbp1, by = "id")


df.temp1 = fun.max24(df.vitals_1, df.admit1, "meas_value", "temp_admit", wide = 0,
↪  "TEMPERATURE", maxtime = 86400)
df1 = full_join(df1, df.temp1, by="id")

df.resp1 = fun.max24(df.vitals_1, df.admit1, "meas_value", "resp_admit", wide = 0,
↪  "RESPIRATIONS", maxtime = 86400)
df1 = full_join(df1, df.resp1, by="id")

df.gcs1 = fun.max24(df.flowsheet_1, df.admit1, "meas_value", "gcs_admit", wide = 0, "R
↪  CPN GLASGOW COMA SCALE SCORE", maxtime = 86400)
df1 = full_join(df1, df.gcs1, by = "id")


df.sbp2 = fun.max24(df.bp2, df.admit2, "sbp", "sbp_admit", wide = 1, "sbp", maxtime =
↪  86400); df2 = full_join(df2, df.sbp2, by = "id")
df.dbp2 = fun.max24(df.bp2, df.admit2, "dbp", "dbp_admit", wide = 1, "dbp", maxtime =
↪  86400); df2 = full_join(df2, df.dbp2, by = "id")

df.sbp2 = fun.max24(df.bpart2, df.admit2, "sbp", "sbpart_admit", wide = 1, "sbp", maxtime
↪  = 86400); df2 = full_join(df2, df.sbp2, by = "id")
df.dbp2 = fun.max24(df.bpart2, df.admit2, "dbp", "dbpart_admit", wide = 1, "dbp", maxtime
↪  = 86400); df2 = full_join(df2, df.dbp2, by = "id")

df.temp2 = fun.max24(df.vitals_2, df.admit2, "meas_value", "temp_admit", wide = 0,
↪  "TEMPERATURE", maxtime = 86400)
df2 = full_join(df2, df.temp2, by="id")

df.resp2 = fun.max24(df.vitals_2, df.admit2, "meas_value", "resp_admit", wide = 0,
↪  "RESPIRATIONS", maxtime = 86400)
df2 = full_join(df2, df.resp2, by="id")

df.gcs2 = fun.max24(df.flowsheet_2, df.admit2, "meas_value", "gcs_admit", wide = 0, "R
↪  CPN GLASGOW COMA SCALE SCORE", maxtime = 86400)
```

© 2023 Nolley EP et al. *JAMA Network Open.*

```
df2 = full_join(df2, df.gcs2, by = "id")

##################################################################
###   SBP, DBP, Temp, Resp, GCS on Mechanical Ventilation      ###
##################################################################
df.sbp1_mv = fun.max24(df.bp1, df.mv1, "sbp", "sbp_mv", wide = 1, "sbp", maxtime =
↪ 86400); df1 = full_join(df1, df.sbp1_mv, by = "id")
df.dbp1_mv = fun.max24(df.bp1, df.mv1, "dbp", "dbp_mv", wide = 1, "dbp", maxtime =
↪ 86400); df1 = full_join(df1, df.dbp1_mv, by = "id")

df.sbp1_mv = fun.max24(df.bpart1, df.mv1, "sbp", "sbpart_mv", wide = 1, "sbp", maxtime =
↪ 86400); df1 = full_join(df1, df.sbp1_mv, by = "id")
df.dbp1_mv = fun.max24(df.bpart1, df.mv1, "dbp", "dbpart_mv", wide = 1, "dbp", maxtime =
↪ 86400); df1 = full_join(df1, df.dbp1_mv, by = "id")

df.temp1_mv = fun.max24(df.vitals_1, df.mv1, "meas_value", "temp_mv", wide = 0,
↪ "TEMPERATURE", maxtime = 86400)
df1 = full_join(df1, df.temp1_mv, by="id")

df.resp1_mv = fun.max24(df.vitals_1, df.mv1, "meas_value", "resp_mv", wide = 0,
↪ "RESPIRATIONS", maxtime = 86400)
df1 = full_join(df1, df.resp1_mv, by="id")

df.gcs1_mv = fun.max24(df.flowsheet_1, df.mv1, "meas_value", "gcs_mv", wide = 0, "R CPN
↪ GLASGOW COMA SCALE SCORE", maxtime = 86400)
df1 = full_join(df1, df.gcs1_mv, by = "id")


df.sbp2_mv = fun.max24(df.bp2, df.mv2, "sbp", "sbp_mv", wide = 1, "sbp", maxtime =
↪ 86400); df2 = full_join(df2, df.sbp2_mv, by = "id")
df.dbp2_mv = fun.max24(df.bp2, df.mv2, "dbp", "dbp_mv", wide = 1, "dbp", maxtime =
↪ 86400); df2 = full_join(df2, df.dbp2_mv, by = "id")

df.sbp2_mv = fun.max24(df.bpart2, df.mv2, "sbp", "sbpart_mv", wide = 1, "sbp", maxtime =
↪ 86400); df2 = full_join(df2, df.sbp2_mv, by = "id")
df.dbp2_mv = fun.max24(df.bpart2, df.mv2, "dbp", "dbpart_mv", wide = 1, "dbp", maxtime =
↪ 86400); df2 = full_join(df2, df.dbp2_mv, by = "id")

df.temp2_mv = fun.max24(df.vitals_2, df.mv2, "meas_value", "temp_mv", wide = 0,
↪ "TEMPERATURE", maxtime = 86400)
df2 = full_join(df2, df.temp2_mv, by="id")

df.resp2_mv = fun.max24(df.vitals_2, df.mv2, "meas_value", "resp_mv", wide = 0,
↪ "RESPIRATIONS", maxtime = 86400)
df2 = full_join(df2, df.resp2_mv, by="id")

df.gcs2_mv = fun.max24(df.flowsheet_2, df.mv2, "meas_value", "gcs_mv", wide = 0, "R CPN
↪ GLASGOW COMA SCALE SCORE", maxtime = 86400)
df2 = full_join(df2, df.gcs2_mv, by = "id")


df.pulse1_mv = fun.max24(df.vitals_1, df.mv1, "meas_value", "pulse_mv", wide = 0,
↪ "PULSE", maxtime = 86400)
```

```r
df1 = full_join(df1, df.pulse1_mv, by="id")

df.pulse2_mv = fun.max24(df.vitals_2, df.mv2, "meas_value", "pulse_mv", wide = 0, "PULSE", maxtime = 86400)
df2 = full_join(df2, df.pulse2_mv, by="id")

df.sat1_mv = fun.max24(df.vitals_1, df.mv1, "meas_value", "sat_mv", wide = 0, "PULSE OXIMETRY", maxtime = 86400)
df1 = full_join(df1, df.sat1_mv, by="id")

df.sat2_mv = fun.max24(df.vitals_2, df.mv2, "meas_value", "sat_mv", wide = 0, "PULSE OXIMETRY", maxtime = 86400)
df2 = full_join(df2, df.sat2_mv, by="id")


bplist1 = c("h_sbp_admit", "h_sbp_admit", "l_sbp_admit", "l_sbp_admit", "h_sbp_mv", "h_sbp_mv", "l_sbp_mv", "l_sbp_mv")
bplist2 = c("h_sbpart_admit", "h_sbpart_admit", "l_sbpart_admit", "l_sbpart_admit", "h_sbpart_mv", "h_sbpart_mv", "l_sbpart_mv", "l_sbpart_mv")

for(i in 1:length(bplist1)){
  df1[[bplist1[i]]] = ifelse(is.na(df1[[bplist2[i]]]), df1[[bplist1[i]]], df1[[bplist2[i]]])
  df2[[bplist1[i]]] = ifelse(is.na(df2[[bplist2[i]]]), df2[[bplist1[i]]], df2[[bplist2[i]]])
}

###############################
###   Respiratory values    ###
###############################
meas = c("R JHH IP RT FIO2 (OXYGEN THERAPY)", "R JHH IP RT WALL CPAP FIO2 %", "R JHH RT SET FIO2", "R JHM IP MEASURED FIO2",
         "R JHM IP RT FIO2", "R JHM IP RT FIO2 (%) 3", "R JHM IP RT HEATED HIGH FLOW FIO2 (%)", "R FIO2")

df.fio1_mv = fun.max24(df.rta_1, df.mv1, "meas_value", "fio2_mv", wide = 0, meas, maxtime = 86400)
df1 = full_join(df1, df.fio1_mv, by="id")

df.fio2_mv = fun.max24(df.flowsheet_2, df.mv2, "meas_value", "fio2_mv", wide = 0, meas, maxtime = 86400)
df2 = full_join(df2, df.fio2_mv, by="id")


df.o2flow_admit1 = fun.max24(df.flowsheet_1, df.admit1, "meas_value", "o2flow_admit", wide = 0, c("R OXYGEN FLOW RATE", "R JHH IP RT OXYGEN FLOW (L/MIN) WALL CPAP"), maxtime = 86400)
df1 = full_join(df1, df.o2flow_admit1, by="id")

df.o2flow_admit2 = fun.max24(df.flowsheet_2, df.admit2, "meas_value", "o2flow_admit", wide = 0, c("R OXYGEN FLOW RATE", "R JHH IP RT OXYGEN FLOW (L/MIN) WALL CPAP"), maxtime = 86400)
df2 = full_join(df2, df.o2flow_admit2, by="id")
```

© 2023 Nolley EP et al. *JAMA Network Open.*

```
df.sofa_mv1 = fun.max24(df.flowsheet_1, df.mv1, "meas_value", "sofa_mv", wide = 0, "R
  ↪   SOFA SCORE", maxtime = 86400)
df1 = full_join(df1, df.sofa_mv1, by="id")

df.sofa_mv2 = fun.max24(df.flowsheet_2, df.mv2, "meas_value", "sofa_mv", wide = 0, "R
  ↪   SOFA SCORE", maxtime = 86400)
df2 = full_join(df2, df.sofa_mv2, by="id")


####################################################################################
###    Low-flow oxygen, HFNC & NPPV data from admission to Mechanical ventilation       ###
####################################################################################
df1 = fun.admission_to_mv(datain = df.flowsheet_1, data = df1, varname = "o2", dataadmit
  ↪   = df.admit1, datamv0 = df.mv1,
                              meas = c("R OXYGEN FLOW RATE (L/MIN)"))

df2 = fun.admission_to_mv(datain = df.flowsheet_2, data = df2, varname = "o2", dataadmit
  ↪   = df.admit2, datamv0 = df.mv2,
                                      meas = c("R OXYGEN FLOW RATE", "*OLD* R JHM IP PT NEW OXYGEN
                                        ↪   FLOW RATE"))

df1$lowflow <- df2$lowflow <- 0
df1$lowflow = replace(as.numeric(df1$lowflow), df1$o2 <= 6, 1)
df2$lowflow = replace(as.numeric(df2$lowflow), df2$o2 <= 6, 1)

df1 = fun.admission_to_mv(datain = df.flowsheet_1, data = df1, varname = "hfnc",
  ↪   dataadmit = df.admit1, datamv0 = df.mv1,
                                       meas = c("R JHM IP RT HEATED HIGH FLOW DEVICE"))

df2 = fun.admission_to_mv(datain = df.flowsheet_2, data = df2, varname = "hfnc",
  ↪   dataadmit = df.admit2, datamv0 = df.mv2,
                                       meas = c("R JHM IP RT HEATED HIGH FLOW DEVICE"))

df1$hfnc = replace(df1$hfnc, df1$hfnc == "Nasal Cannula", 1)
df1$hfnc = replace(df1$hfnc, is.na(df1$hfnc), 0)
df1$hfnc = replace(df1$hfnc, df1$hfnc == "Trach Connect", 1)


df2$hfnc = replace(df2$hfnc, df2$hfnc == "Nasal Cannula", 1)
df2$hfnc = replace(df2$hfnc, is.na(df2$hfnc) | df2$hfnc == "Other (see comments)", 0)
df2$hfnc = replace(df2$hfnc, df2$hfnc == "Trach Connect", 1)


nppv = c("R JHM IP RT NPPV DEVICE", "R JHH RT NPPV MODE", "R JHH RT NPPV DELIVERY", "R
  ↪   JHH RT NPPV RR TOTAL")
df1 = fun.admission_to_mv(datain = df.flowsheet_1, data = df1, varname = "nppv",
  ↪   dataadmit = df.admit1, datamv0 = df.mv1, meas = nppv)
df2 = fun.admission_to_mv(datain = df.flowsheet_2, data = df2, varname = "nppv",
  ↪   dataadmit = df.admit2, datamv0 = df.mv2, meas = nppv)

df1$nppv = ifelse(is.na(df1$nppv), 0, 1)
```

© 2023 Nolley EP et al. *JAMA Network Open*.

```r
df2$nppv = ifelse(is.na(df2$nppv), 0, 1)

#########################
###    Comorbidities    ###
#########################
icd10_list = c("J44.", #=====================> copd
               "J45.", #=====================> asthma
               "I50.", #=====================> chf
               "N18.", #=====================> ckd
               "N18.6", #=====================> esrd
               "D84.|Z94.", #=====================> immunosupressed
               "Z33.", #=====================> pregnant
               "F17.21", #=====================> current smoker
               "Z87.891", #=====================> former smoker
               "R41.0", #=====================> confusion
               "I21|I22|I25.2", #=====================> mi
               "I71|I79.0|I73.9|R02|Z95.8|Z95.9", #=====================> pvd

                ↪ "I60|I61|I62|I63|I65|I66|G45.0|G45.1|G45.2|G45.8|G45.9|G46|I64|G45.4|I67.0|I67.1|I67
               I67.8|I67.9|I68.1|I68.2|I68.8|I69", #=====================> cvd
               "F00|F01|F02|F05.1", #=====================> dementia
               "J40|J41|J42|J44|J43|J45|J46|J47|J67|J44|J60|J61|J62|J63|J66|J64|J65",
                ↪ #=====================> pulmonary

                ↪ "M32|M34|M33.2|M05.3|M05.8|M05.9|M06.0|M06.3|M06.9|M05.0|M05.2|M05.1|M35.3",
                ↪ #=====================> connective
               "K25|K26|K27|K28",#=====================>  pid

                ↪ "K70.2|K70.3|K73|K71.7|K74.0|K74.2|K74.6|K74.3|K74.4|K74.5",#=====================>
                ↪ liver

                ↪ "E10.9|E11.9|E13.9|E14.9|E10.1|E11.1|E13.1|E14.1|E10.5|E11.5|E13.5|E14.5",#=========
                ↪ dm

                ↪ "E10.2|E11.2|E13.2|E14.2|E10.3|E11.3|E13.3|E14.3|E10.4|E11.4|E13.4|E14.4",#=========
                ↪ dm_cx
               "G81|G04.1|G82.0|G82.1|G82.2",#=====================>  paraplegia

                ↪ "N03|N05.2|N05.3|N05.4|N05.5|N05.6|N07.2|N07.3|N07.4|N01|N18|N19|N25",#=============
                ↪ renal

                ↪ "C0|C1|C2|C3|C40|C41|C43|C45|C46|C47|C48|C49|C5|C6|C70|C71|C72|C73|C74|C75|C76|C80|C

 ↪ C90.0|C90.1|C91|C92|C93|C94.0|C94.1|C94.2|C94.3|C94.51|C94.7|C95|C96",#=====================>
 ↪ cancer
               "C77|C78|C79|C80",#=====================> metastatic
               "K72.9|K76.6|K76.7|K72.1",#=====================> severeliver
               "B20|B21|B22|B23|B24",#=====================> hiv
               "C95.9",#=====================> leukemia
               "C85.90",#=====================> lymphoma
               "E11.9",#====================>   uncomplicated  dm
               "E11.22", #=====================> end organ damage dm
               "K74.69|K70.30|K74.60|K71.7|K74|K70.31",   #====================>   Cirrhois
```

© 2023 Nolley EP et al. *JAMA Network Open.*

```
                    "C90.", #====================> Multiple myeloma
                    "K91.82|K70.41|K70.40|K12.01|K72",   #====================>   Hepatic   failure

                    ↪ "J12.89|J10.00|J12.9|J10.08|J11.08|U07.1|J12.0|J12.3|J09.X1|J10.01|J11.00|J12.2|J12.
                    ↪ #====================> Viral pneumonia

                    ↪ "J15.9|J15.0|B95.3|B96.1|J13|J14|J15.5|J15.212|J15.211|J15.7|J15.6|J15.8|J15.29|J15.
                    ↪ #====================> Bacterial
                    "J16.8|B37.1|J18.1", #====================> Fungal
                    "J69.0", #=====================> Aspiration
                    "J95.851", #=====================> vap
                    "J18.9|J18.0|J18.8")     #====================> unspecified

diseaselist = c("copd", "asthma", "chf", "ckd", "esrd", "immunosuppressed", "pregnant",
 ↪  "current_smoker", "former_smoker",
                     "confusion",
                     "mi",
                     "pvd",
                     "cvd",
                     "dementia",
                     "pulmonary",
                     "connective",
                     "pid",
                     "liver",
                     "diabetes",
                     "diabetes_cx",
                     "paraplegia",
                     "renal",
                     "cancer",
                     "metastatic",
                     "severeliver",
                     "hiv",
                     "leukemia",
                     "lymphoma",
                     "uncomplicated_dm",
                     "endorgan_dm",
                     "corrhosis",
                     "myeloma",
                     "hepfailure",
                     "viral",
                     "bacterial",
                     "fungal",
                     "aspiration",
                     "vap",
                    "pneumonia with unspecified organism")

for(i in c(1:length(icd10_list))){
  dfx  =  fun.morb(df.billingdx,  icd10_list[i],  diseaselist[i])
  dfx = dfx %>% dplyr::distinct()
  df1 = full_join(df1, dfx, by="id")
}

for(i in c(1:length(icd10_list))){
```

© 2023 Nolley EP et al. *JAMA Network Open.*

```
  dfy  =  fun.morb(df.billingdx_ctrl,  icd10_list[i],  diseaselist[i])
  dfy = dfy %>% dplyr::distinct()
  df2 = full_join(df2, dfy, by="id")
}

#######################
###    Lab results      ###
#######################
df.lab_1$meas_name = replace(df.lab_1$meas_name, df.lab_1$component_name == "SODIUM",
 ↪  "SODIUM")
df.lab_2$meas_name = replace(df.lab_2$meas_name, df.lab_2$component_name == "SODIUM",
 ↪  "SODIUM")

list1 = c("CREATININE", "BILITOT", "WBC", "LDH", "DDIMER", "CRP", "PLT", "CKTOTAL",
 ↪  "BUN")
list2 = c("cr_admit", "tbili_admit", "wbc_admit", "ldh_admit", "ddimer_admit",
 ↪  "cpr_admit", "plt_admit", "cpk_admit", "bun_admit")

for(i in c(1:length(list1))){
  dfx = fun.48_24(df.lab_1, df.admit1, "ord_value", list2[i], list1[i], mintime =
 ↪  -172800, maxtime = 86400)
  df1 = full_join(df1, dfx, by="id")
}

for(i in c(1:length(list1))){
  dfy = fun.48_24(df.lab_2, df.admit2, "ord_value", list2[i], list1[i], mintime =
 ↪  -172800, maxtime = 86400)
  df2 = full_join(df2, dfy, by="id")
}

dfx  =  fun.48_24(df.lab_1,  df.admit1,  "ord_value",  "lymph_admit",  c("LYMPHABSMAN",
 ↪  "LYMPHOABS"), mintime = -172800, maxtime = 86400)
df1 = full_join(df1, dfx, by="id")

dfy  =  fun.48_24(df.lab_2,  df.admit2,  "ord_value",  "lymph_admit",  c("LYMPHABSMAN",
 ↪  "LYMPHOABS"), mintime = -172800, maxtime = 86400)
df2 = full_join(df2, dfy, by="id")

list3 = c("CREATININE", "SODIUM", "K", "HCT", "WBC", "PLT", "BILITOT", "BUN", "ALBUMIN")
list4 = c("cr_mv", "sodium_mv", "potassium_mv", "hematocrit_mv", "wbc_mv", "plt_mv",
           "tbili_mv", "bun_mv", "albumin_mv")

for(i in c(1:length(list3))){
  dfx  =  fun.48_24(df.lab_1,  df.mv1,  "ord_value",  list4[i],  list3[i],  mintime  =  -172800,
 ↪  maxtime = 86400)
  df1 = full_join(df1, dfx, by="id")
}

for(i in c(1:length(list3))){
  dfy  =  fun.48_24(df.lab_2,  df.mv2,  "ord_value",  list4[i],  list3[i],  mintime  =  -172800,
 ↪  maxtime = 86400)
  df2 = full_join(df2, dfy, by="id")
}
```

© 2023 Nolley EP et al. *JAMA Network Open.*

```
dfx = fun.48_24(df.lab_1, df.mv1, "ord_value", "glu_mv", c("GLU", "GLUPOC"), mintime = -172800, maxtime = 86400)
df1 = full_join(df1, dfx, by="id")

dfy = fun.48_24(df.lab_2, df.mv2, "ord_value", "glu_mv", c("GLU", "GLUPOC"), mintime = -172800, maxtime = 86400)
df2 = full_join(df2, dfy, by="id")

dfx = fun.48_24(df.lab_1, df.mv1, "ord_value", "ph_mv", c("PHART", "ISTATPH"), mintime = -172800, maxtime = 86400)
df1 = full_join(df1, dfx, by="id")

dfy = fun.48_24(df.lab_2, df.mv2, "ord_value", "ph_mv", c("PHART", "ISTATPH"), mintime = -172800, maxtime = 86400)
df2 = full_join(df2, dfy, by="id")

dfx = fun.48_24(df.lab_1, df.mv1, "ord_value", "pco2_mv", c("PCO2ART", "ISTATPCO22"), mintime = -172800, maxtime = 86400)
df1 = full_join(df1, dfx, by="id")

dfy = fun.48_24(df.lab_2, df.mv2, "ord_value", "pco2_mv", c("PCO2ART", "ISTATPCO22"), mintime = -172800, maxtime = 86400)
df2 = full_join(df2, dfy, by="id")

dfx = fun.48_24(df.lab_1, df.mv1, "ord_value", "pao2_mv", c("PO2ART", "ISTATPO2"), mintime = 0, maxtime = 172800)
df1 = full_join(df1, dfx, by="id")

dfy = fun.48_24(df.lab_2, df.mv2, "ord_value", "pao2_mv", c("PO2ART", "ISTATPO2"), mintime = 0, maxtime = 172800)
df2 = full_join(df2, dfy, by="id")

dfx = fun.48_24.mean(df.lab_1, df.mv1, "ord_value", "pao2_mv", c("PO2ART", "ISTATPO2"), mintime = 0, maxtime = 172800)
df1 = full_join(df1, dfx, by="id")

dfy = fun.48_24.mean(df.lab_2, df.mv2, "ord_value", "pao2_mv", c("PO2ART", "ISTATPO2"), mintime = 0, maxtime = 172800)
df2 = full_join(df2, dfy, by="id")

########################
###    vasopressors     ###
########################
df.sofamed = df.med %>%
  dplyr::filter(PharmSubClass == "Cardiovascular Sympathomimetics") %>%
  dplyr::filter(grepl("Given|New Bag|Restarted", MAR_Action)) %>%
  dplyr::filter(grepl("DOBUTAMINE|DOPAMINE|EPINEPHRINE|NOREPINEPHRINE", MedName)) %>%
  dplyr::filter(grepl("mcg/kg/min", DoseUnit)) %>%
  dplyr::rename(id = osler_id)

df.sofamed$med = substr(df.sofamed$MedName, 1, 3)
df.sofamed = full_join(df.sofamed, df.mv1, by = "id")
```

© 2023 Nolley EP et al. *JAMA Network Open.*

```
df.sofamed$timediff = ymd_hms(df.sofamed$TakenTime) - ymd_hms(df.sofamed$hospdate)
df.sofamed = df.sofamed %>%
  dplyr::filter(timediff <= 86400 & timediff >=0) %>%
  dplyr::select(id, med, Dose, timediff) %>%
  dplyr::arrange(id, timediff) %>%
  dplyr::distinct(id, med, .keep_all=TRUE) %>%
  spread(med, Dose) %>%
  dplyr::select(-timediff)  %>%
  dplyr::rename(dob_mv = DOB, nor_mv = NOR, dop_mv = DOP, epi_mv = EPI) %>%
  group_by(id) %>%
  fill(everything(), .direction = "down") %>%
  fill(everything(), .direction = "up") %>%
  slice(1)

df.sofamed$dob_mv = NA
df.sofamed$dop_mv = NA

df.sofamed2 = df.med_ctrl %>%
  dplyr::filter(pharm_subclassname == "Cardiovascular Sympathomimetics") %>%
  dplyr::filter(grepl("Given|New Bag|Restarted", mar_action)) %>%
  dplyr::filter(grepl("DOBUTAMINE|DOPAMINE|EPINEPHRINE|NOREPINEPHRINE", medication_name))
  ↪ %>%
  dplyr::filter(grepl("mcg/kg/min", dose_unit)) %>%
  dplyr::rename(id = osler_id, dose = sig)

df.sofamed2$med = substr(df.sofamed2$medication_name, 1, 3)
df.sofamed2 = full_join(df.sofamed2, df.mv2, by = "id")
df.sofamed2$timediff = ymd_hms(df.sofamed2$taken_time) - ymd_hms(df.sofamed2$hospdate)
df.sofamed2 = df.sofamed2 %>%
  dplyr::filter(timediff <= 86400 & timediff >=0) %>%
  dplyr::select(id, med, dose, timediff) %>%
  dplyr::arrange(id, timediff) %>%
  dplyr::distinct(id, med, .keep_all=TRUE) %>%
  spread(med, dose) %>%
  dplyr::select(-timediff)  %>%
  dplyr::rename(dob_mv = DOB, nor_mv = NOR, dop_mv = DOP, epi_mv = EPI) %>%
  dplyr::relocate(nor_mv, .after = dob_mv) %>%
  group_by(id) %>%
  fill(everything(), .direction = "down") %>%
  fill(everything(), .direction = "up") %>%
  slice(1)

df1 = full_join(df1, df.sofamed, by = "id")
df2 = full_join(df2, df.sofamed2, by = "id")

#####################################################
### Tracheostomy, intubation, extubation dates ###
#####################################################
df.intube1 = fun.firstdate(df.flowsheet_1, df.admit1, "PLACEMENT DATE", "intubedate" ,
  ↪ recorded_time=1 , wide=0)
df.intube2 = fun.firstdate(df.flowsheet_2, df.admit2, "PLACEMENT DATE", "intubedate" ,
  ↪ recorded_time=1 , wide=0)
```

© 2023 Nolley EP et al. *JAMA Network Open.*

```
df1 = full_join(df1, df.intube1, by = "id")
df2 = full_join(df2, df.intube2, by = "id")

df.extube1 = fun.firstdate(df.flowsheet_1, df.admit1, "REMOVAL DATE", "extubedate" ,
↪ recorded_time=1 , wide=0)
df.extube2 = fun.firstdate(df.flowsheet_2, df.admit2, "REMOVAL DATE", "extubedate" ,
↪ recorded_time=1 , wide=0)

#colnames(df.extube1) = colnames(df.extube2) = c("id", "extubedate")
df1 = full_join(df1, df.extube1, by = "id")
df2 = full_join(df2, df.extube2, by = "id")

####################################################
###    Weight and Age on Mechanical Ventilation      ###
####################################################
df.weight1_mv = fun.firstmeas.before72(df.vitals_1, df.mv1, "WEIGHT/SCALE", "weight_mv");
↪ df1 = full_join(df1, df.weight1_mv, by="id")
df.weight2_mv = fun.firstmeas.before72(df.vitals_2, df.mv2, "WEIGHT/SCALE", "weight_mv");
↪ df2 = full_join(df2, df.weight2_mv, by="id")

df1$weight_mv = as.numeric(as.character(df1$weight_mv))*0.0283495
df2$weight_mv  =  as.numeric(as.character(df2$weight_mv))*0.0283495

df.mv1 = df.mv1 %>% dplyr::rename(mvdate = hospdate)
df.mv2 = df.mv2 %>% dplyr::rename(mvdate = hospdate)

df1 = left_join(df1, df.mv1, by = "id")
df2 = left_join(df2, df.mv2, by = "id")

df1$age_admit = as.period(interval(as.Date(df1$birth_date), as.Date(df1$hospdate)),unit =
↪ "years")$year
df2$age_admit = as.period(interval(as.Date(df2$birth_date), as.Date(df2$hospdate)),unit =
↪ "years")$year

df1$age_mv = as.period(interval(as.Date(df1$birth_date), as.Date(df1$mvdate)),unit =
↪ "years")$year
df2$age_mv = as.period(interval(as.Date(df2$birth_date), as.Date(df2$mvdate)),unit =
↪ "years")$year

d1 = right_join(df1, df.mv1)
d2 = right_join(df2, df.mv2)
df = rbind(d1, d2)

##############################
###    Recoding variables      ###
##############################
### Recoding ma0fe1
df$ma0fe1 = ifelse(df$ma0fe1 == "Male", 0,
                          ifelse(df$ma0fe1 == "Female", 1, NA))

df$lymph_admit = ifelse(df$l_lymph_admit < 1.1, 1,
                         ifelse(df$l_lymph_admit >= 1.1, 0, NA))
```

© 2023 Nolley EP et al. *JAMA Network Open.*

```
########################################
###     Charlson  Comorbidity  Index     ###
########################################
df$charl_age = replace(df$charl_age, df$age_mv < 50, 0)
df$charl_age = replace(df$charl_age, df$age_mv >= 50 & df$age <= 59, 1)
df$charl_age = replace(df$charl_age, df$age_mv >= 60 & df$age <= 69, 2)
df$charl_age = replace(df$charl_age, df$age_mv >= 70 & df$age <= 79, 3)
df$charl_age = replace(df$charl_age, df$age_mv >= 80, 4)

df$charl_severeliver = ifelse(df$severeliver == 1, 3)
df$charl_diabetes_cx = ifelse(df$diabetes_cx == 1, 2)
df$charl_paraplegia = ifelse(df$paraplegia == 1, 2)
df$charl_renal = ifelse(df$renal == 1, 2)
df$charl_cancer = ifelse(df$cancer == 1, 2)
df$charl_metastatic = ifelse(df$metastatic == 1, 6)
df$charl_leukemia = ifelse(df$leukemia == 1, 2)
df$charl_lymphoma = ifelse(df$lymphoma == 1, 2)
df$charl_hiv = ifelse(df$hiv == 1, 6)

for(i in c("charl_age", diseaselist,
              "charl_severeliver","diabetes","charl_diabetes_cx","charl_paraplegia","charl_renal","cha
            "charl_metastatic","charl_leukemia","charl_lymphoma","charl_hiv")){
  df[[i]] = replace(df[[i]], is.na(df[[i]]), 0)
}

df$charlson_mv = df$charl_age+ df$mi+ df$chf+ df$pvd+ df$cvd+ df$dementia+ df$copd+
   df$connective+ df$pid+ df$liver+
                     df$charl_severeliver+ df$diabetes+ df$charl_diabetes_cx+
   df$charl_paraplegia+ df$charl_renal+ df$charl_cancer+
                      df$charl_metastatic+  df$charl_leukemia+  df$charl_lymphoma+  df$charl_hiv


df$intubeday = as.period(interval(lubridate::mdy(01012016),
   as.Date(as.character(df$intubedate))),unit = "days")$day
df$extubeday = as.period(interval(lubridate::mdy(01012016), as.Date(df$extubedate)),unit
   = "days")$day
df$day_death = as.period(interval(lubridate::mdy(01012016), as.Date(df$date_death)),unit
   = "days")$day
df$day_discharge = as.period(interval(lubridate::mdy(01012016),
   as.Date(df$date_discharge)),unit = "days")$day
df$hospday2 = as.period(interval(lubridate::mdy(01012016), as.Date(df$hospdate)),unit =
   "days")$day

### If death then discharge is missing
df$date_discharge = replace(df$date_discharge , as.Date(df$date_discharge) ==
   as.Date(df$date_death), NA)
df$day_discharge = replace(df$day_discharge , df$day_discharge == df$day_death, NA)


for(i in colnames(df)){
  df[[i]] = ifelse(is.infinite(df[[i]]), NA, df[[i]])
```

© 2023 Nolley EP et al. *JAMA Network Open.*

```
}

for(i in
  c("diabetes","asthma","copd","chf","ckd","esrd","immunosuppressed","pregnant","current_smoker","for
  "confusion")){
  df[[i]] = replace(df[[i]], is.na(df[[i]]), 0)
}

######################
###   SOFA score    ###
######################
df$sofa_pafi = df$l_pao2_mv / (df$l_fio2_mv/100)
df$sofa_pafi = cut(df$sofa_pafi, breaks=c(-Inf, 99, 199, 299, 399, Inf),
  labels=c(4,3,2,1,0))
df$sofa_coag = cut(df$l_plt_mv, breaks=c(-Inf, 19, 49, 99, 149, Inf),
  labels=c(4,3,2,1,0))
df$sofa_gcs = cut(df$l_gcs_mv, breaks=c(-Inf, 5, 9, 12, 14, Inf), labels=c(4,3,2,1,0))
df$sofa_bili = cut(df$h_tbili_mv, breaks=c(-Inf, 1.19, 1.99, 5.99, 11.99, Inf),
  labels=c(0,1,2,3,4))

df$sofa_renal = replace(df$sofa_renal, df$h_cr_mv < 1.2, 0)
df$sofa_renal = replace(df$sofa_renal, df$h_cr_mv >= 1.2 & df$h_cr_mv <= 1.9, 1)
df$sofa_renal = replace(df$sofa_renal, df$h_cr_mv < 2 & df$h_cr_mv <= 3.4, 2)
df$sofa_renal = replace(df$sofa_renal, (df$h_cr_mv < 3.5 & df$h_cr_mv <= 4.9) | df$urine
  < 500, 3)
df$sofa_renal = replace(df$sofa_renal, df$h_cr_mv > 5 | df$urine < 200, 4)

df$sofa_vaso = replace(df$sofa_vaso, df$l_map_mv > 70, 0)
df$sofa_vaso = replace(df$sofa_vaso, df$l_map_mv < 70, 1)
df$sofa_vaso = replace(df$sofa_vaso, df$dop_mv <= 5 | df$dob_mv > 0, 2)
df$sofa_vaso = replace(df$sofa_vaso, df$dop_mv > 5 | df$epi_mv <= 0.1 | df$nor_mv <= 0.1,
  3)
df$sofa_vaso = replace(df$sofa_vaso, df$dop_mv > 15 | df$epi_mv > 0.1 | df$nor_mv > 0.1,
  4)

for(i in c("sofa_pafi", "sofa_coag", "sofa_gcs", "sofa_bili", "sofa_renal",
  "sofa_vaso")){
df[[i]] = replace(df[[i]], is.na(df[[i]]), 0)
df[[i]] = as.numeric(df[[i]])
}

df$sofa_calc = df$sofa_pafi + df$sofa_coag + df$sofa_vaso + df$sofa_renal + df$sofa_gcs +
  df$sofa_bili

###################
###   CURB-65   ###
###################
df$curb65_age = ifelse(df$age_admit >= 65, 1, 0)
df$curb65_bp = ifelse(df$l_sbp_admit < 90 | df$l_dbp_admit <= 60, 1, 0)
df$curb65_rr = ifelse(df$h_resp_admit >= 30, 1, 0)
df$curb65_bun = ifelse(df$h_bun_admit > 19, 1, 0)
df$curb65_confusion = ifelse(df$confusion == 1, 1, 0)
df$curb65 = df$curb65_age + df$curb65_bp + df$curb65_rr + df$curb65_bun +
  df$curb65_confusion
```

© 2023 Nolley EP et al. *JAMA Network Open.*

```r
##############################
###     Making IDs unique     ###
##############################
df$id = paste0(df$id, "_", as.character(as.Date(df$hospdate)))
df$date_discharge  =  ifelse(is.na(df$date_discharge),  df$date_death,  df$date_discharge)

##############################################################
###     Correcting extreme values and recoding variables     ###
##############################################################
df$height[df$emrn == ""] = 182.9 # EMR omitted here
df$height[df$emrn == ""] = 165.1 # EMR omitted here

df$bmi[df$group == "Non-COVID19" & as.numeric(df$bmi) > 160] = NA

df$fever_l = NA
df$fever_l = replace(df$fever_l, df$l_temp_admit >= 100.4, 1)
df$fever_l = replace(df$fever_l, df$l_temp_admit < 100.4, 0)

df$fever_h  =  NA
df$fever_h = replace(df$fever_h, df$l_temp_admit >= 100.4, 1)
df$fever_h = replace(df$fever_h, df$l_temp_admit < 100.4, 0)

df$h_pao2_mv_imp = fun.pao2.imp(df$h_sat_mv/100)
df$l_pao2_mv_imp  =  fun.pao2.imp(df$l_sat_mv/100)

df$l_pao2_mv  =  ifelse(is.na(df$l_pao2_mv),  df$l_pao2_mv_imp,  df$l_pao2_mv)

df$l_pafi = df$l_pao2_mv / (df$l_fio2_mv/100)
df$l_pafi  =  replace(df$l_pafi,  df$l_pafi  >  1500  |  is.na(df$l_pafi),  NA)

df$group2 = ifelse(df$group == "COVID19", 1, 0)
```

```r
################################
###     Long format dataset     ###
################################
df.daily  =  df  %>%
  dplyr::select(id, date_death, date_discharge, height, ma0fe1, group, mvdate)

for(i in c("date_death", "date_discharge", "mvdate")){
  df.daily[[i]] = as.Date(df.daily[[i]])
}

df.daily$date_discharge  =  as.Date(ifelse(is.na(df.daily$date_discharge),
 ↪  as.character(df.daily$date_death),
                                         as.character(df.daily$date_discharge)))

df.daily$date_discharge = replace(df.daily$date_discharge,
 ↪  is.na(df.daily$date_discharge),   data.date)

df.daily$maxday  =
 ↪  ifelse(!is.na(df.daily$date_discharge),as.numeric(df.daily$date_discharge  -
 ↪  df.daily$mvdate),
```

© 2023 Nolley EP et al. *JAMA Network Open.*

```
                              ifelse(is.na(df.daily$date_discharge), as.numeric(data.date -
                              ↪ df.daily$mvdate), NA))
df.daily = df.daily %>% filter(maxday >= 0)


dat = NULL
for(i in unique(df.daily$id)){
  ddd = df.daily %>% dplyr::filter(id == i)

  xx = data.frame("id" = rep(ddd$id, ddd$maxday+1),
                  "mvday" = c(0 : ddd$maxday),
                  "mvdate" = seq(as.Date(ddd$mvdate), as.Date(ddd$date_discharge),
                  ↪ "days"),
                  "height" = rep(ddd$height, ddd$maxday+1),
                  "ma0fe1" = rep(ddd$ma0fe1, ddd$maxday+1),
                  "group" = rep(ddd$group, ddd$maxday+1))
  dat = rbind(dat, xx)
}

dat$pbw = ifelse(dat$ma0fe1 == 0, 50+(0.91*(dat$height - 152.4)), 45.5+(0.91*(dat$height
↪ - 152.4)))


### Mechanical ventilation data
df.mv3 = fun.vent.long(df.flowsheet_1, df.mv1$id, df.admit1)
df.mv4 = fun.vent.long(df.flowsheet_2, df.mv2$id, df.admit2)

dat1 = dat %>% dplyr::filter(group == "COVID19") %>% distinct()
dat2 = dat %>% dplyr::filter(group == "Non-COVID19") %>% distinct()

dat1 = full_join(dat1, df.mv3, by = c("id", "mvdate"))
dat2 = full_join(dat2, df.mv4, by = c("id", "mvdate"))

dat1 = dat1 %>% dplyr::filter(!is.na(mvday))
dat2 = dat2 %>% dplyr::filter(!is.na(mvday))

dat1$mechvent = ifelse(is.na(dat1$date), 0, 1)
dat2$mechvent = ifelse(is.na(dat2$date), 0, 1)

######################
###   Prone data   ###
######################
df.pos1 = left_join(df.flowsheet_1, df.admit1, by="id") %>%
  dplyr::filter(meas_name == "R ORTHOSTATIC POSITION") %>%
  dplyr::filter(grepl("Prone|prone", meas_value)) %>%
  dplyr::mutate(id = paste0(id, "_", as.character(as.Date(hospdate)))) %>%
  dplyr::mutate(mvdate = as.Date(recorded_time)) %>%
  dplyr::select(id, meas_value, mvdate) %>%
  dplyr::rename(position = meas_value) %>%
  dplyr::distinct()

df.pos2 = left_join(df.flowsheet_2, df.admit2, by="id") %>%
  dplyr::filter(meas_name == "R ORTHOSTATIC POSITION") %>%
```

© 2023 Nolley EP et al. *JAMA Network Open.*

```
  dplyr::filter(grepl("Prone|prone", meas_value)) %>%
  dplyr::mutate(id = paste0(id, "_", as.character(as.Date(hospdate)))) %>%
  dplyr::mutate(mvdate = as.Date(recorded_time)) %>%
  dplyr::select(id, meas_value, mvdate) %>%
  dplyr::rename(position = meas_value) %>%
  dplyr::distinct()

dat1 = left_join(dat1, df.pos1, by = c("id", "mvdate")) %>%
  dplyr::arrange(desc(position)) %>%
  distinct(id, mvday, .keep_all = TRUE)

dat2 = left_join(dat2, df.pos2, by = c("id", "mvdate")) %>%
  dplyr::arrange(desc(position)) %>%
  distinct(id, mvday, .keep_all = TRUE)

dat1$position = ifelse(is.na(dat1$position), 0, 1)
dat2$position = ifelse(is.na(dat2$position), 0, 1)

### Respiratory measurements
list1 = list(
            "R JHM IP RT SET/TARGET TIDAL VOLUME (ML)",      #"R JHM IP RT SET/TARGET TIDAL
             ↪ VOLUME (ML)",
            "R JHH RT NPPV INSPIRATORY TIME",
            "R JHM IP RT SET RATE (BPM)",      #"R JHM IP RT SET RATE (BPM)",
            "R JHM IP RT RR TOTAL",
            "R JHM IP RT MINUTE VENTILATION (L/MIN)",               #    R JHM IP RT MINUTE
             ↪ VENTILATION (L/MIN
            "R JHH IP RT PEEP (CMH2O)",                             #"R JHH IP RT PEEP (CMH2O)",
            "R JHM IP RT PLATEAU PRESSURE (CMH2O)",                 #"R JHM IP RT PLATEAU
             ↪ PRESSURE (CMH2O)",
            "R JHM IP RT MEAN AIRWAY PRESSURE (CMH2O)",             #"R JHM IP RT MEAN AIRWAY
             ↪ PRESSURE (CMH2O)",
            "R JHM IP RT PEAK AIRWAY PRESSURE (CMH2O)",             #"R JHM IP RT PEAK AIRWAY
             ↪ PRESSURE (CMH2O)",
            "R IP RT JHM COMPLIANCE CALC",
            "R JHM IP RT VENTILATOR MODES",
            "R JHM IP RT SPO2",
            c("R JHH IP RT PAO2/FIO2 RATIO (MMHG)", "R JHM IP PAO2/FIO2"))

list2 = c("vt", "ti", "rrset", "rrtot", "mv", "peep", "pplat", "pmean", "pmax", "c_rs",
 ↪ "mode", "spo2", "pafi")
minlist = c(-Inf, -Inf, -Inf, -Inf, -Inf, 5, 10, -Inf, -Inf, -Inf, -Inf, -Inf, -Inf)
maxlist = c(2000, Inf, Inf, Inf, Inf, 24, 50, Inf, Inf, Inf, Inf, Inf, Inf)

for(i in c(1:length(list1))){
  dat1 = fun.firstmeas.day(df.flowsheet_1, dat1, list1[i], list2[i], 3, df.admit1,
 ↪ minlist[i], maxlist[i])
  dat2 = fun.firstmeas.day(df.flowsheet_2, dat2, list1[i], list2[i], 3, df.admit2,
 ↪ minlist[i], maxlist[i])
}

dat1 = fun.firstmeas.day(df.flowsheet_1, dat1,
                         c("R JHM IP RT EXHALED TIDAL VOLUME (ML)", "R JHM IP RT
                          ↪ SPONTANEOUS EXHALED TIDAL VOLUME"),
```

© 2023 Nolley EP et al. *JAMA Network Open.*

```
                                   "vt_exh", 3, df.admit1, -Inf, Inf)

dat1 = fun.firstmeas.day(df.flowsheet_1, dat1,
                                   c("R JHM IP RT FIO2 (%) 3","R FIO2", "R JHH IP RT FIO2 (OXYGEN
                                     ↪   THERAPY)", "R JHH RT SET FIO2"),
                                   "fio2", 3, df.admit1, -Inf, Inf)

dat2 = fun.firstmeas.day(df.flowsheet_2, dat2,
                                   c("R JHM IP RT EXHALED TIDAL VOLUME (ML)", "R JHM IP RT
                                     ↪   SPONTANEOUS EXHALED TIDAL VOLUME"),
                                     "vt_exh", 3, df.admit2, -Inf, Inf)

dat2 = fun.firstmeas.day(df.flowsheet_2, dat2,
                                   c("R JHM IP RT FIO2 (%) 3","R FIO2", "R JHH IP RT FIO2 (OXYGEN
                                     ↪   THERAPY)"),
                                   "fio2", 3, df.admit2, -Inf, Inf)

dat1$vtpbw  =  as.numeric(dat1$vt)  /  as.numeric(dat1$pbw)
dat2$vtpbw = as.numeric(dat2$vt) / as.numeric(dat2$pbw)

dat1$pdriv = as.numeric(dat1$pplat) - as.numeric(dat1$peep)
dat2$pdriv  =  as.numeric(dat2$pplat)  -  as.numeric(dat2$peep)

dat1$c_rs = ifelse(as.numeric(dat1$c_rs)  <0  |  as.numeric(dat1$c_rs) > 300, NA,
 ↪   as.numeric(dat1$c_rs))
dat2$c_rs = ifelse(as.numeric(dat2$c_rs)  <0  |  as.numeric(dat2$c_rs) > 300, NA,
 ↪   as.numeric(dat2$c_rs))

dat1$spo2 = ifelse(as.numeric(dat1$spo2)   <0  | as.numeric(dat1$spo2) > 100, NA,
 ↪   as.numeric(dat1$spo2))
dat2$spo2 = ifelse(as.numeric(dat2$spo2)   <0  | as.numeric(dat2$spo2) > 100, NA,
 ↪   as.numeric(dat2$spo2))

dat1  = fun.48_24.mean.mv0(df.flowsheet_1, dat1, df.admit1, df.mv1, "meas_value",
 ↪   "spo2_mv0", "R JHM IP RT SPO2", mintime = 0, maxtime = 172800)
dat2  = fun.48_24.mean.mv0(df.flowsheet_2, dat2, df.admit2, df.mv2, "meas_value",
 ↪   "spo2_mv0", "R JHM IP RT SPO2", mintime = 0, maxtime = 172800)

dat1$spo2_mv0  =  ifelse(as.numeric(dat1$spo2_mv0)  <0  |  as.numeric(dat1$spo2_mv0)  >  100,
 ↪   NA,  as.numeric(dat1$spo2_mv0))
dat2$spo2_mv0  =  ifelse(as.numeric(dat2$spo2_mv0)  <0  |  as.numeric(dat2$spo2_mv0)  >  100,
 ↪   NA,  as.numeric(dat2$spo2_mv0))

dat1  = fun.48_24.mean.mv0(df.flowsheet_1, dat1, df.admit1, df.mv1, "meas_value",
 ↪   "pafi_mv0", c("R JHH IP RT PAO2/FIO2 RATIO (MMHG)|R JHM IP PAO2/FIO2"), mintime = 0,
 ↪   maxtime = 172800)
dat2  = fun.48_24.mean.mv0(df.flowsheet_2, dat2, df.admit2, df.mv2, "meas_value",
 ↪   "pafi_mv0", c("R JHH IP RT PAO2/FIO2 RATIO (MMHG)|R JHM IP PAO2/FIO2"), mintime = 0,
 ↪   maxtime = 172800)

dat1  = fun.48_24.mean.mv0(df.flowsheet_1, dat1, df.admit1, df.mv1, "meas_value",
 ↪   "fio2_mv0", c("R JHM IP RT FIO2 (%) 3|R FIO2|R JHH IP RT FIO2 (OXYGEN THERAPY)"),
 ↪   mintime = 0, maxtime = 172800)
```

© 2023 Nolley EP et al. *JAMA Network Open.*

```
dat2 = fun.48_24.mean.mv0(df.flowsheet_2, dat2, df.admit2, df.mv2, "meas_value",
↪   "fio2_mv0", c("R JHM IP RT FIO2 (%) 3|R FIO2|R JHH IP RT FIO2 (OXYGEN THERAPY)"),
↪   mintime = 0, maxtime = 172800)

################
###   ECMO   ###
################
df.ecmo_1 = df.flowsheet_1 %>% filter((str_detect(meas_name, "ECMO")) |
↪   (str_detect(meas_value, "ECMO")))
df.ecmo_2 = df.flowsheet_2 %>% filter((str_detect(meas_name, "ECMO")) |
↪   (str_detect(meas_value, "ECMO")))

######################
###   Lab values   ###
######################
lab.list1 = c("PHART", "PO2ART", "PCO2ART", "PCO2NONART", "PLT", "HGB", "WBC",
↪   "CREATININE", "DDIMER", "CRP", "LDH", "PHNONAR", c("LYMPHABSMAN", "LYMPHOABS"))

lab.list2 = c("ph", "pao2", "paco2", "vpaco2", "plt", "hb", "wbc", "cr", "ddimer", "crp",
↪   "ldh", "vph", "lymph")

for(i in 1:length(lab.list1)){
  dat1 = fun.firstmeas.day(df.lab_1, dat1, lab.list1[i], lab.list2[i], 32, df.admit1,
↪   -Inf, Inf)
  dat2 = fun.firstmeas.day(df.lab_2, dat2, lab.list1[i], lab.list2[i], 36, df.admit2,
↪   -Inf, Inf)
}

dat1 = fun.48_24.mean.mv0(df.lab_1, dat1, df.admit1, df.mv1, "ord_value", "pao2_mv0",
↪   c("PO2ART|ISTATPO2"), mintime = 0, maxtime = 172800)
dat2 = fun.48_24.mean.mv0(df.lab_2, dat2, df.admit2, df.mv2, "ord_value", "pao2_mv0",
↪   c("PO2ART|ISTATPO2"), mintime = 0, maxtime = 172800)

### Converting PaO2<30 into missing
dat1$pao2 = as.numeric(dat1$pao2)
dat2$pao2 = as.numeric(dat2$pao2)
dat1$pao2 = ifelse(dat1$pao2 < 30, NA, dat1$pao2)
dat2$pao2 = ifelse(dat2$pao2 < 30, NA, dat2$pao2)

dat1$pao2_mv0 = as.numeric(dat1$pao2_mv0)
dat2$pao2_mv0 = as.numeric(dat2$pao2_mv0)
dat1$pao2_mv0 = ifelse(dat1$pao2_mv0 < 30, NA, dat1$pao2_mv0)
dat2$pao2_mv0 = ifelse(dat2$pao2_mv0 < 30, NA, dat2$pao2_mv0)

### Ventilator mode
dat1$mode = ifelse(dat1$mode == "PRVC", 2,
                   ifelse(dat1$mode == "SIMV", 3,
                          ifelse(dat1$mode == "PCV", 4,
                                 ifelse(dat1$mode == "PSV", 5,
                                        ifelse(dat1$mode == "APRV", 6, 7)))))

dat2$mode = ifelse(dat2$mode == "PRVC", 2,
                   ifelse(dat2$mode == "SIMV", 3,
```

© 2023 Nolley EP et al. *JAMA Network Open.*

```
                            ifelse(dat2$mode == "PCV", 4,
                                   ifelse(dat2$mode == "PSV", 5,
                                          ifelse(dat2$mode == "APRV", 6, 7)))))

### Blood pressure

df.bp1_long = fun.bp(df.bp1, df.admit1)
df.bp2_long = fun.bp(df.bp2, df.admit2)

df.bpart1_long = fun.bp(df.bpart1, df.admit1)
df.bpart2_long = fun.bp(df.bpart2, df.admit2)

df.bp1_long = full_join(df.bp1_long, df.bpart1_long, by = c("id", "mvdate", "hospdate"))
 ↪  %>%
  mutate(sbp = ifelse(is.na(sbp.y), sbp.x, sbp.y),
         dbp = ifelse(is.na(dbp.y), dbp.x, dbp.y)) %>%
  dplyr::select(-contains(".x"), -contains(".y"))

df.bp2_long = full_join(df.bp2_long, df.bpart2_long, by = c("id", "mvdate", "hospdate"))
 ↪  %>%
  mutate(sbp = ifelse(is.na(sbp.y), sbp.x, sbp.y),
         dbp = ifelse(is.na(dbp.y), dbp.x, dbp.y)) %>%
  dplyr::select(-contains(".x"), -contains(".y"))

dat1 = left_join(dat1, df.bp1_long, by = c("id", "mvdate"))
dat2 = left_join(dat2, df.bp2_long, by = c("id", "mvdate"))

df.bp1_lhm = left_join(df.bp1, df.admit1, by="id") %>%
  dplyr::mutate(mvdate = as.Date(recorded_time)) %>%
  dplyr::mutate(id = paste0(id, "_", as.character(as.Date(hospdate)))) %>%
  dplyr::group_by(id, mvdate) %>%
  dplyr::summarise(sbp_low = min(as.numeric(sbp), na.rm = TRUE),
            sbp_high = max(as.numeric(sbp), na.rm = TRUE),
            sbp_mean = mean(as.numeric(sbp), na.rm = TRUE),
            dbp_low = min(as.numeric(dbp), na.rm = TRUE),
            dbp_high = max(as.numeric(dbp), na.rm = TRUE),
            dbp_mean = mean(as.numeric(dbp), na.rm = TRUE))

df.bp2_lhm = left_join(df.bp2, df.admit2, by="id") %>%
  dplyr::mutate(mvdate = as.Date(recorded_time)) %>%
  dplyr::mutate(id = paste0(id, "_", as.character(as.Date(hospdate)))) %>%
  dplyr::group_by(id, mvdate) %>%
  dplyr::summarise(sbp_low = min(as.numeric(sbp), na.rm = TRUE),
            sbp_high = max(as.numeric(sbp), na.rm = TRUE),
            sbp_mean = mean(as.numeric(sbp), na.rm = TRUE),
            dbp_low = min(as.numeric(dbp), na.rm = TRUE),
            dbp_high = max(as.numeric(dbp), na.rm = TRUE),
            dbp_mean = mean(as.numeric(dbp), na.rm = TRUE))

dat1 = left_join(dat1, df.bp1_lhm, by = c("id", "mvdate"))
dat2 = left_join(dat2, df.bp2_lhm, by = c("id", "mvdate"))

dat1 = fun.lhm(df.flowsheet_1, "R JHM IP RT SPO2", "meas_value","spo2", dat1, df.admit1)
dat2 = fun.lhm(df.flowsheet_2, "R JHM IP RT SPO2", "meas_value","spo2", dat2, df.admit2)
```

© 2023 Nolley EP et al. *JAMA Network Open.*

```
dat1 = fun.lhm(df.lab_1, "PO2ART", "ord_value", "pao2", dat1, df.admit1)
dat2 = fun.lhm(df.lab_2, "PO2ART", "ord_value", "pao2", dat2, df.admit2)

dat1 = fun.lhm(df.lab_1, c("LACTATE", "LACTATEWB"), "ord_value", "lactate", dat1,
   df.admit1)
dat2 = fun.lhm(df.lab_2, c("LACTATE", "LACTATEWB"), "ord_value", "lactate", dat2,
   df.admit2)

dat1 = fun.today(df.med_1, "class", c("VASOPRESSOR"), df.med_1$route, "vaso", dat1, 0,
   df.admit1)
dat2 = fun.today(df.med_2, "class", c("VASOPRESSO"), df.med_2$route, "vaso", dat2, 0,
   df.admit2)

dat1 = fun.today(df.med_1, "class", c("VASODILATORS"), c("Inhalation", "Nebulization"),
   "ino", dat1, 0, df.admit1)
dat2 = fun.today(df.med_2, "class", c("VASODILATORS"), c("Inhalation", "Nebulization"),
   "ino", dat2, 0, df.admit2)

dat1 = fun.today(df.med_1, "name", "REMDESIVIR", df.med_1$route, "remdesivir", dat1, 0,
   df.admit1)
dat2 = fun.today(df.med_2, "name", "REMDESIVIR", df.med_1$route, "remdesivir", dat2, 0,
   df.admit2)

dat1 = fun.today(df.med_1, "class", "NEUROMUSCULAR BLOCKING AGENTS", df.med_1$route,
   "nmb", dat1, 0, df.admit1)
dat2 = fun.today(df.med_2, "class", "NEUROMUSCULAR BLOCKING AGENTS", df.med_1$route,
   "nmb", dat2, 0, df.admit2)

dat1 = fun.today(df.med_1, "name", "DEXAMETHASONE", df.med_1$route, "dexa", dat1, 6,
   df.admit1)
dat2 = fun.today(df.med_2, "name", "DEXAMETHASONE", df.med_1$route, "dexa", dat2, 6,
   df.admit2)

dat1 = fun.today(df.med_1, "name", "PREDNISONE", df.med_1$route, "prednisone", dat1, 40,
   df.admit1)
dat2 = fun.today(df.med_2, "name", "PREDNISONE", df.med_1$route, "prednisone", dat2, 40,
   df.admit2)

dat1 = fun.today(df.med_1, "name", "METHYLPREDNISOLONE", df.med_1$route, "prednisolone",
   dat1, 32, df.admit1)
dat2 = fun.today(df.med_2, "name", "METHYLPREDNISOLONE", df.med_1$route, "prednisolone",
   dat2, 32, df.admit2)

dat1 = fun.today(df.med_1, "name", "HYDROCORTISONE", df.med_1$route, "cortisone", dat1,
   200, df.admit1)
dat2 = fun.today(df.med_2, "name", "HYDROCORTISONE", df.med_1$route, "cortisone", dat2,
   200, df.admit2)

dat1$steroids = ifelse(dat1$dexa == 1 | dat1$prednisone == 1 | dat1$prednisolone == 1 |
   dat1$cortisone == 1, 1, 0)
dat2$steroids = ifelse(dat2$dexa == 1 | dat2$prednisone == 1 | dat2$prednisolone == 1 |
   dat2$cortisone == 1, 1, 0)
```

© 2023 Nolley EP et al. *JAMA Network Open.*

```r
### Making sure that the IDs are the same in long and wide format
dat1 = dat1 %>%
  dplyr::filter(id %in% df$id)

dat2 = dat2 %>%
  dplyr::filter(id %in% df$id)

data = rbind(dat1, dat2)

##########################################
###   Recoding Fio2 extreme values    ###
##########################################
ddd = data %>% filter(as.numeric(fio2) < 21) %>%
  dplyr::select(id, mvdate, fio2) %>%
  left_join(df[,c("id", "emrn")], by = "id") %>%
  mutate(id = substr(id, 1, 36),
         mvdate = as.Date(mvdate),
         fio2 = as.numeric(fio2))

dxd1 = df.flowsheet_1 %>%
  filter(id %in% ddd$id & meas_name %in% c("R JHM IP RT FIO2 (%) 3","R FIO2", "R OXYGEN
  ↪ DEVICE")) %>%
  mutate(mvdate = as.Date(recorded_time))

dxd2 = df.flowsheet_2 %>%
  filter(id %in% ddd$id & meas_name %in% c("R JHM IP RT FIO2 (%) 3","R FIO2", "R OXYGEN
  ↪ DEVICE")) %>%
  mutate(mvdate = as.Date(recorded_time))

dxd = rbind(dxd1, dxd2)

dxd = dxd %>%
  dplyr::select(-meas_template_id) %>%
  right_join(ddd, by = c("id", "mvdate")) %>%
  filter(meas_name == "R OXYGEN DEVICE" | meas_value == fio2) %>%
  group_by(id, meas_name) %>%
  arrange(recorded_time)

dxd.fio2 = dxd %>%
  filter(meas_name %in% c("R JHM IP RT FIO2 (%) 3","R FIO2")) %>%
  distinct(id, mvdate, .keep_all = TRUE)
dxd.device = dxd %>% filter(meas_name == "R OXYGEN DEVICE")

dxd = full_join(dxd.fio2, dxd.device, by = c("id", "mvdate", "emrn", "fio2")) %>%
  mutate(timediff = ymd_hms(recorded_time.x) - ymd_hms(recorded_time.y)) %>%
  filter(timediff >= 0) %>%
  arrange(id, mvdate, timediff) %>%
  group_by(id, mvdate) %>%
  distinct(id, mvdate, .keep_all = TRUE) %>%
  filter(meas_value.y == "None-room air") %>%
  dplyr::select(id, mvdate)
```

© 2023 Nolley EP et al. *JAMA Network Open.*

```r
data$fio2 = replace(data$fio2, as.numeric(data$fio2) < 21, NA)
data$fio2[substr(data$id, 1, 36) %in% dxd$id & data$mvdate %in% dxd$mvdate] = 21
table(as.numeric(data$fio2))

###########################
###    PaO2 imputation    ###
###########################
data$pao2_imp = fun.pao2.imp(data$spo2/100)
data$pao2 = ifelse(is.na(data$pao2), data$pao2_imp, data$pao2)

data$pao2mv0_imp = fun.pao2.imp(data$spo2_mv0/100)
data$pao2_mv0 = ifelse(is.na(data$pao2_mv0), data$pao2mv0_imp, data$pao2_mv0)

#################################
###    Calculating PaO2/FiO2   ###
#################################
data$fio2 = as.numeric(data$fio2)/100
data$fio2_mv0 = as.numeric(data$fio2_mv0)/100
data$pao2 = as.numeric(data$pao2)
range(data$fio2, na.rm = T)

data$pafi_calc = as.numeric(data$pao2)/as.numeric(data$fio2)
data$pafi_calc_mv0 = as.numeric(data$pao2_mv0)/as.numeric(data$fio2_mv0)

data$pafi = ifelse(is.na(data$pafi_calc), data$pafi, data$pafi_calc)
data$pafi_mv0 = ifelse(is.na(data$pafi_calc_mv0), data$pafi_mv0, data$pafi_calc_mv0)

data = data %>% dplyr::filter(!is.na(group)) %>% distinct()
df.age = df %>% dplyr::select(id, age_admit)
data = left_join(data, df.age, by = "id")

df.weight = df %>% dplyr::select(id, weight_mv)
data = left_join(data, df.weight, by = "id") %>% filter(!is.na(mvday))

########################################
###    Calculating Mechanical power    ###
########################################
for(i in c("rrtot", "pmax", "vt", "pplat", "peep")){
  data[[i]] = as.numeric(data[[i]])
}
data$mech_power = 0.098*data$rrtot*(data$vt/1000)*(data$pmax-(0.5*(data$pplat -
  data$peep)))

#########################################
###    Calculating Ventilatory ratio     ###
#########################################
data$paco2_
data$vent_ratio =
  ((as.numeric(data$mv)*1000)*as.numeric(data$paco2))/(as.numeric(data$weight_mv)*100*37.5)

#####################################
###    Calculating ARDS severity      ###
#####################################
```

© 2023 Nolley EP et al. *JAMA Network Open.*

```
data$paficat = ifelse(data$pafi_mv0 <= 150, 1,
                      ifelse(data$pafi_mv0 > 150, 0, NA))

data$ards = ifelse(data$pafi_mv0 <= 100, "PF < 100",
               ifelse(data$pafi_mv0 > 100 & data$pafi_mv0 <= 200, "PF 100-199",
                  ifelse(data$pafi_mv0 > 200 & data$pafi_mv0 <= 300, "PF 200-299",
                         ifelse(data$pafi_mv0 > 300, "PF >= 300", NA))))

####################################
###   Dropping Missing PaO2/FiO2    ###
####################################
ddd = data %>%
  filter(mvday == 0) %>%
  filter(is.na(pafi_mv0))     %>%
  dplyr::select(id, pafi_mv0, pafi_calc_mv0, pao2_mv0, fio2, spo2_mv0, group, fio2_mv0,
  ↪  mvdate)

### Dropping if patient was not on mechanical ventilator within 14 days from admission
df = df %>% filter(!(id %in% ddd$id)) %>% filter(as.Date(mvdate) - as.Date(hospdate) <=
 ↪  14)
df.daily = df.daily %>% filter(!(id %in% ddd$id)) %>% filter(id %in% df$id)
data = data %>% filter(!(id %in% ddd$id)) %>% filter(id %in% df$id)

### Dropping the patients who had tracheostomy on admission
df = df %>%
  filter(!(substr(id, 1, 36) %in% df.trach1$id)) %>%
  filter(!(substr(id, 1, 36) %in% df.trach2$id))

data = data %>%
  filter(!(substr(id, 1, 36) %in% df.trach1$id)) %>%
  filter(!(substr(id, 1, 36) %in% df.trach2$id))
```

Dataset version: 06/30/2021

```
# Seed
set.seed(443527)
# Directory
dir = getwd()
data.date = lubridate::mdy(06302021)

list.of.packages <- c("tidyverse", "RODBC", "plyr", "dplyr", "gmodels", "lubridate",
 ↪  "data.table", "comorbidity", "gtools", "tibbletime",
                      "cowplot", "gtable", "grid", "gridExtra", "reshape2", "ggpubr",
                       ↪  "Rfit", "MatchIt", "optmatch", "knitr",
                      "Hmisc", "furniture", "janitor", "kableExtra", "lubridate",
                       ↪  "cobalt", "flextable", "broom", "lmtest", "sandwich", "lemon",
                       ↪  "caret",  "MASS")

new.packages  <-  list.of.packages[!(list.of.packages  %in%
 ↪  installed.packages()[,"Package"])]
if(length(new.packages)) {
```

© 2023 Nolley EP et al. *JAMA Network Open.*

```
  install.packages(new.packages)
}
```

```
dat = read.csv("dat.csv")
df = read.csv("df.csv")
df.daily = read.csv("df.daily.csv")

vap <- df %>% dplyr::filter(vap == 1)

dat <- dat %>% dplyr::filter(!(id %in% vap$id))
df <- df[!(df$id %in% vap$id), ]
df.daily <- df.daily %>% dplyr::filter(!(id %in% vap$id))

### Checking
dtest = df %>% dplyr::select(id, hospdate, date_discharge, date_death, group)
dtest$deathday = as.Date(dtest$date_death) - as.Date(dtest$hospdate)
dtest$disday = as.Date(dtest$date_discharge) - as.Date(dtest$hospdate)

x = dtest$id[is.na(dtest$date_death) & is.na(dtest$date_discharge)]

# There are two IDs with missing death+discharge date and pt were in the hospital for 90+
↪ days

stillhosp = c("008599da-5fc7-4494-b082-3accd000ef57_2021-06-10",
              "13dfb91a-fd60-4d04-ad58-e367cd0ad49e_2021-06-25",
              "447d54a3-ae83-43b0-ab9d-735e7f443b62_2021-05-10",
              "64fad376-b08d-4c9b-8eab-87662589b69b_2021-04-26",
              "b618b535-4efc-4d48-ace6-9adc7964186b_2021-06-05",
              "d09cfca2-059b-4bf6-bcd8-6e1f7990a8ac_2021-06-29",
              "d4e18b36-fc11-4ba7-a32c-1fb378e73d3d_2021-06-16",
              "f42462a4-69f4-435d-b0fd-c6f7ce5eedb5_2021-04-28",
              "f4a0cb9f-9ea8-47d6-a82d-d3f6e665be1e_2021-04-30")

dat = dat %>% dplyr::filter(!(id %in% stillhosp))
df = df %>% dplyr::filter(!(id %in% stillhosp))
df.daily = df.daily %>% dplyr::filter(!(id %in% stillhosp))

# Converting extreme PEEP values into missing values
dat$peep = ifelse(dat$peep <5 | dat$peep>24, NA, dat$peep)
# Recoding variables
df$race2 = ifelse(df$race %in% c("Am Indian", "Asian", "Black", "Hispanic", "Other",
↪ "Unknown"), 1, 0)
df$race2 = ifelse(df$race == "Unknown", NA, df$race2)

# 1: White or Caucasian
# 2: Black or African American
# 3: American Indian or Alaska Native
# 4: Asian
# 5: Native Hawaiian or Other Pacific Islander
# 6: Other
# 7: Patient Refused
# 8: Unknown
# 9: Two or More Races
```

© 2023 Nolley EP et al. *JAMA Network Open.*

```r
# 10: Declined to Answer
#  11:  Hispanic
#  12:  Native  Hawaiian
#  13:  Other  Pacific  Islander

df$ethnicity2 = ifelse(df$ethnicity == "Unknown" | df$ethnicity == "", NA,
                       ifelse(df$ethnicity == "Hispanic", 1,
                              ifelse(df$ethnicity == "Not Hispanic", 0, df$ethnicity)))
```

```r
d0 = dat %>%
  filter(mvday == 0) %>%
  dplyr::select(id, pafi_mv0, paficat, ards)

dff = left_join(df, d0, by = "id")

#dff$group = factor(dff$group, levels = c("COVID19", "Non-COVID19"), labels =
↪  c("COVID19", "Non-COVID19"))

####################
###    Matching    ###
####################
var.adjusted  =  "age_admit  +  bmi  +  factor(ma0fe1)  +  charlson_mv  +
                            pafi_mv0  +  sofa_calc  +  factor(race2)  +  factor(ethnicity2)"

df.tomatch  =  dff  %>%
  filter(!is.na(bmi)  &  !is.na(age_admit)  &  !is.na(paficat)  &  !is.na(ma0fe1)  &
↪  !is.na(charlson_mv)     &
             !is.na(sofa_calc)  &  !is.na(race2)  &  !is.na(ethnicity2))


ppnsity.model = as.formula(paste0("factor(group2) ~ ", var.adjusted))

set.seed(443527)
### Sampling weight adjusted propensity match estimation
match.out <-     matchit(ppnsity.model, data = df.tomatch, method = "full")

summary(match.out)$nn
```

```
##                     Control  Treated
## All (ESS)         1089.0000      697
## All               1089.0000      697
## Matched (ESS)      104.3005      697
## Matched           1089.0000      697
## Unmatched            0.0000        0
## Discarded            0.0000        0
```

```r
set.seed(443527)
df.matched <- match.data(match.out, distance = "prop.score")

lab = c(bquote(PaO[2]/FiO[2]), "Charlson comorbidity index", "Gender", "Age", "Ethnicity:
↪  Non hispanic", "Race: White",
          "Body mass index", "SOFA score", "Distance")
```

© 2023 Nolley EP et al. *JAMA Network Open.*

```
x =
love.plot(bal.tab(match.out), stat = "mean.diffs", threshold = .1,
          var.order = "unadjusted", size = 7, grid = FALSE) +
  #scale_y_discrete("")
 scale_y_discrete("", labels = lab) +
 scale_x_continuous("Mean differences", limits = c(-1,1.1), breaks = seq(-1, 1, 0.2)) +
 scale_color_manual(values = c("black", "grey")) +
 theme(legend.title = element_blank(),
       legend.position = "bottom",
       plot.title = element_blank(),
       axis.text = element_text(size = 16),
       axis.title = element_text(size = 19),
       legend.text = element_text(size = 16))



df.tomatch$year = substr(df.tomatch$id, 38, 41)
```

Figure 2: Mean differences in key variables before and after propensity (full) matching.

```
plot(x)
```

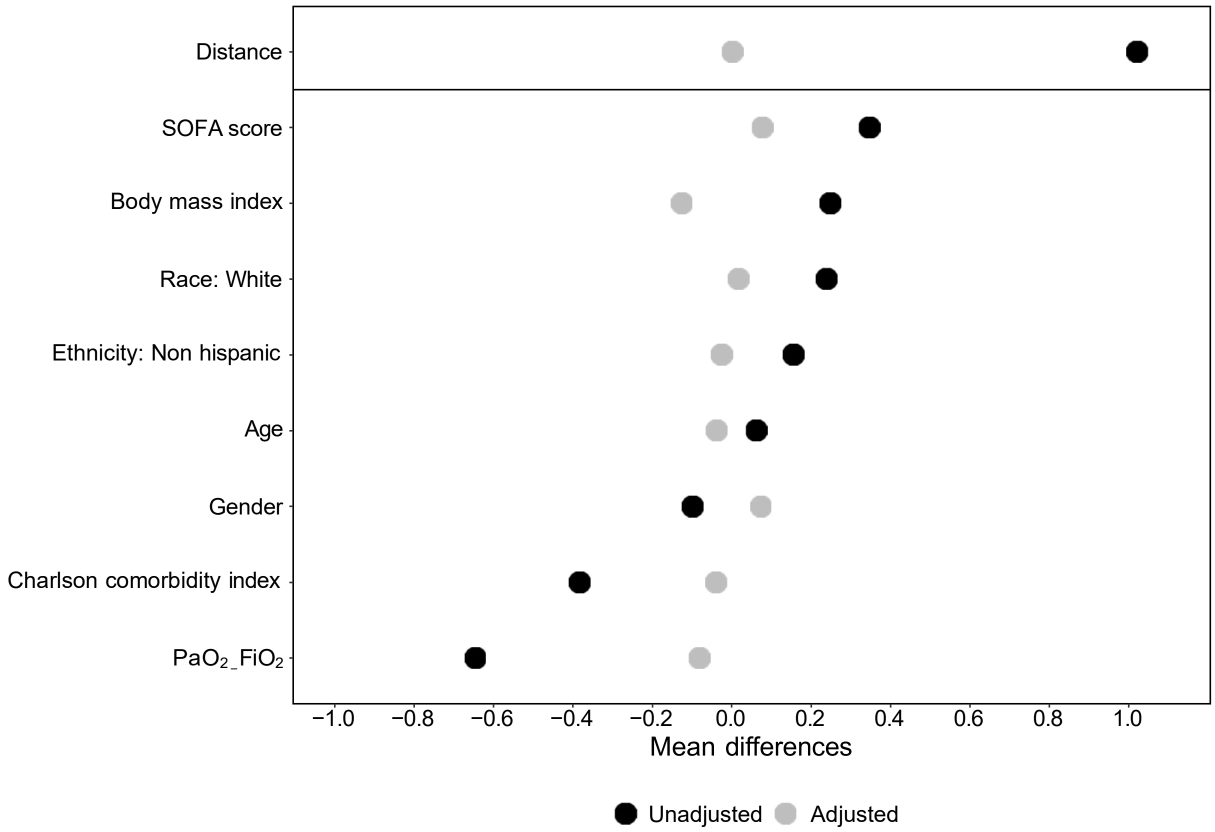

© 2023 Nolley EP et al. *JAMA Network Open.*

```
ggsave(x, filename = "Figure-3.jpg", dpi = 300, type = "cairo",
        width = 12, height = 10, units = "in")

ggsave(x, filename = "Figure-3.tiff", dpi = 300, type = "cairo",
        width = 12, height = 10, units = "in")
```

```
ppnsity.model11 = as.formula(paste0("factor(group2) ~ ", var.adjusted))

set.seed(443527)
match.out11 <-     matchit(ppnsity.model, data = df.tomatch, method = "nearest", ratio = 1)

match.out11$n
```

```
## NULL
```

```
df.matched11 <- match.data(match.out11, distance = "prop.score")

lab = c(bquote(PaO[2]/FiO[2]), "Charlson comorbidity index", "Gender", "Age", "Ethnicity:
 ↪ Non hispanic", "Race: White",
          "Body mass index", "SOFA score", "Distance")

x11 =
  love.plot(bal.tab(match.out11), stat = "mean.diffs", threshold = .1,
             var.order = "unadjusted", size = 7, grid = FALSE) +
  #scale_y_discrete("")
  scale_y_discrete("", labels = lab) +
  scale_x_continuous("Mean differences", limits = c(-1,1.1), breaks = seq(-1, 1, 0.2)) +
  scale_color_manual(values = c("black", "grey")) +
  theme(legend.title = element_blank(),
          legend.position = "bottom",
          plot.title = element_blank(),
          axis.text = element_text(size = 16),
          axis.title = element_text(size = 19),
          legend.text = element_text(size = 16))

ggsave(x11, filename = "Figure-3_11.png", dpi = 300,
        width = 12, height = 10, units = "in")

ppnsity.model21 = as.formula(paste0("factor(group2) ~ ", var.adjusted))
set.seed(443527)
match.out21 <-     matchit(ppnsity.model, data = df.tomatch, method = "nearest", ratio = 2)

match.out21$n
```

```
## NULL
```

```
df.matched21 <- match.data(match.out21, distance = "prop.score")

lab = c(bquote(PaO[2]/FiO[2]), "Charlson comorbidity index", "Gender", "Age", "Ethnicity:
 ↪ Non hispanic", "Race: White",
          "Body mass index", "SOFA score", "Distance")
```

© 2023 Nolley EP et al. *JAMA Network Open.*

```r
x21 =
  love.plot(bal.tab(match.out21), stat = "mean.diffs", threshold = .1,
            var.order = "unadjusted", size = 7, grid = FALSE) +
  #scale_y_discrete("")
  scale_y_discrete("", labels = lab) +
  scale_x_continuous("Mean differences", limits = c(-1,1.1), breaks = seq(-1, 1, 0.2)) +
  scale_color_manual(values = c("black", "grey")) +
  theme(legend.title = element_blank(),
        legend.position = "bottom",
        plot.title = element_blank(),
        axis.text = element_text(size = 16),
        axis.title = element_text(size = 19),
        legend.text = element_text(size = 16))

ggsave(x21, filename = "Figure-3_21.png", dpi = 300,
       width = 12, height = 10, units = "in")
```

Mean differences in key variables before and after propensity (1:1) matching.

```r
plot(x11)
```

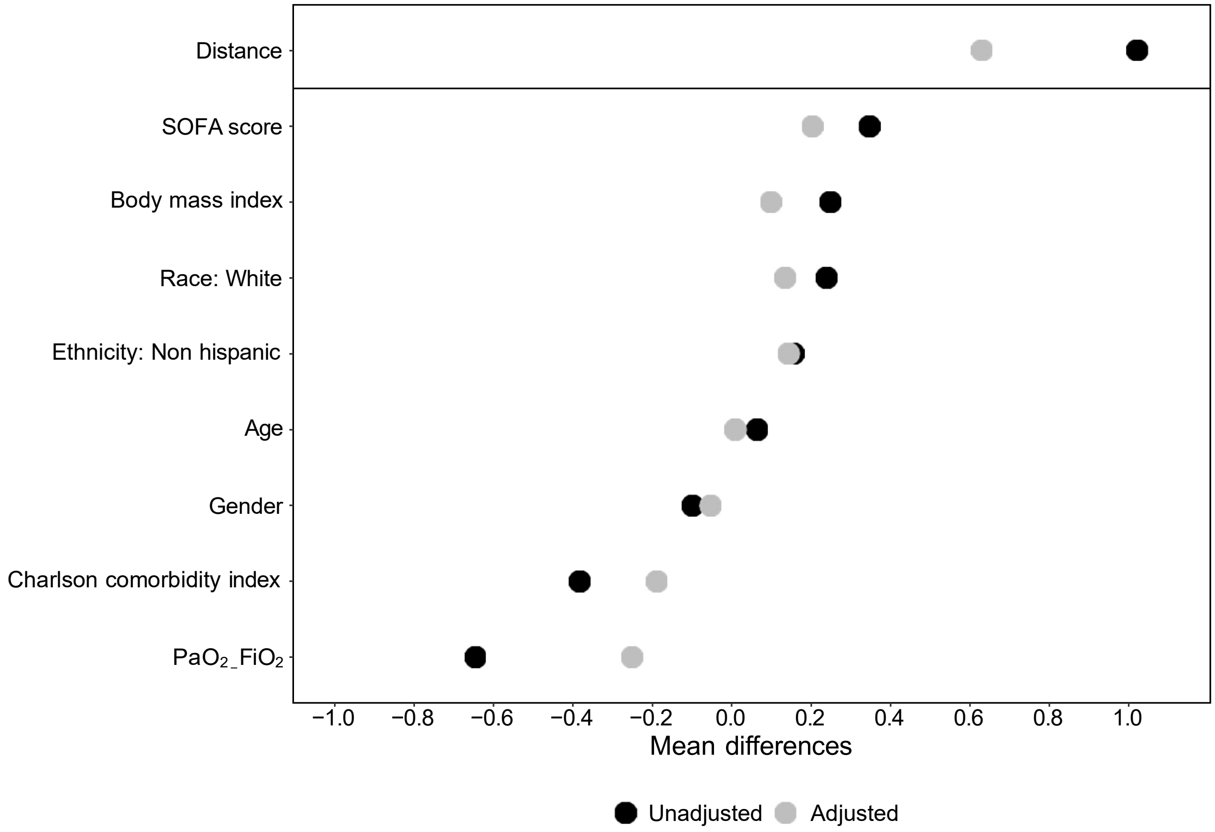

© 2023 Nolley EP et al. *JAMA Network Open.*

Mean differences in key variables before and after propensity (2:1) matching.

```
plot(x21)
```

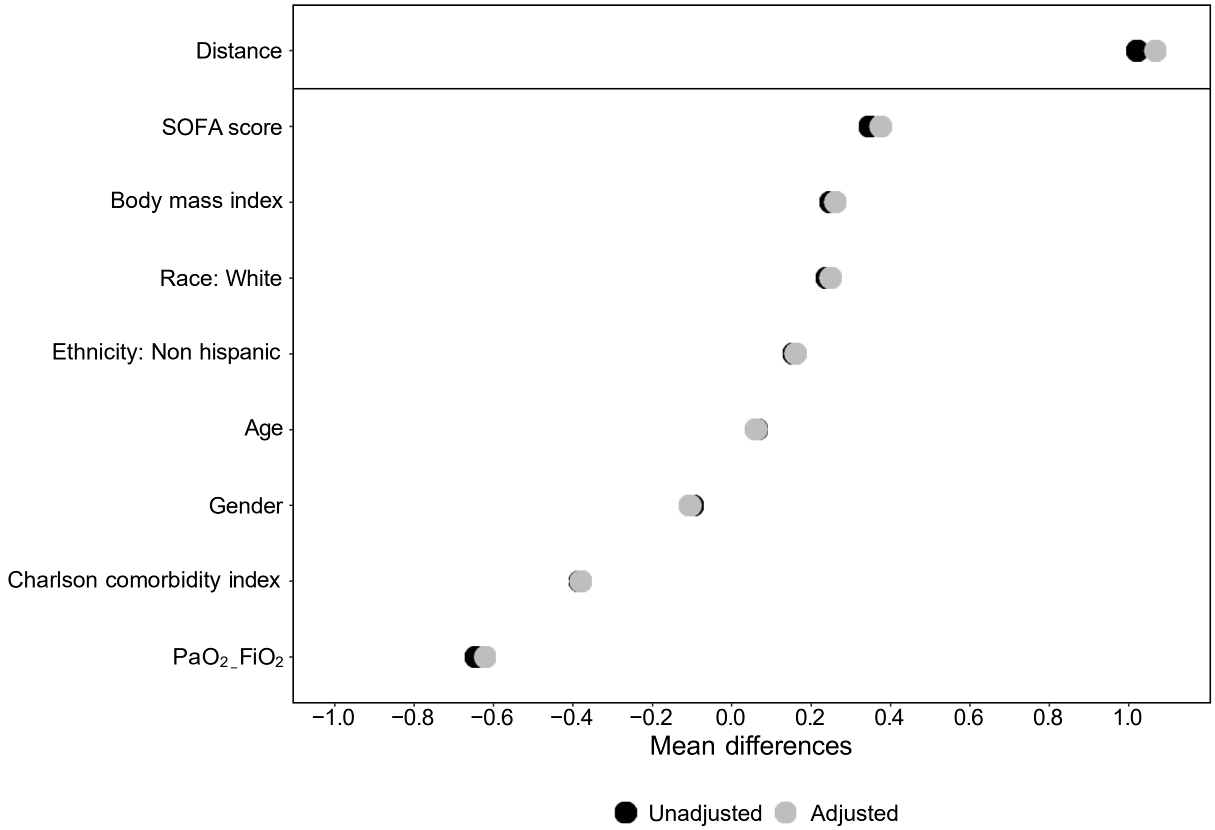

Ventilatory ratio sensitivity analysis

```
dfv <- dat

dfv0 <- dfv %>%
  dplyr::filter(mvday == 0) %>%
  dplyr::select(id, pafi, c_rs)

dfv1 <- left_join(dff, dfv0, by = "id") %>%
  dplyr::filter(pafi < median(pafi, na.rm = TRUE)) %>%
  filter(!is.na(bmi) & !is.na(age_admit) & !is.na(paficat) & !is.na(ma0fe1) &
↪  !is.na(charlson_mv) &
          !is.na(sofa_calc) & !is.na(race2) & !is.na(ethnicity2))
set.seed(443527)
v1.out <-   matchit(ppnsity.model, data = dfv1, method = "full")
dfv1 <- match.data(v1.out, distance = "prop.score")

dfv1x <- dfv %>%
      dplyr::filter(mvday <= 6) %>%
```

© 2023 Nolley EP et al. *JAMA Network Open.*

```
    dplyr::group_by(id)      %>%
    dplyr::summarise(vent_ratio = mean(vent_ratio, na.rm = TRUE)) %>%
    dplyr::right_join(dfv1[, c("id", "weights", "subclass", "group")], by = "id") %>%
    dplyr::select(id, group,vent_ratio , weights, subclass)

dfv1x$group <- factor(dfv1x$group, levels = c("Non-COVID19", "COVID19"))

fit1 <- glm(vent_ratio ~ factor(group), data = dfv1x,
                    weights = weights)
print(summary(fit1 ))
```

```
##
## Call:
## glm(formula = vent_ratio ~ factor(group), data = dfv1x, weights = weights)
##
## Deviance Residuals:
## Min            1Q Median        3Q      Max
##  -2.0138   -0.3237   -0.0761    0.1989   10.9694
##
## Coefficients:
##                          Estimate Std. Error t value Pr(>|t|)
## (Intercept)               1.40494    0.03875  36.257   <2e-16 ***
## factor(group)COVID19 0.01575      0.05639   0.279      0.78
## ---
## Signif. codes:  0 '***' 0.001 '**' 0.01 '*' 0.05 '.' 0.1 ' ' 1
##
## (Dispersion parameter for gaussian family taken to be 0.5403325)
##
##     Null deviance: 356.66  on 661  degrees of freedom ##
Residual deviance: 356.62  on 660  degrees of freedom ##
(123 observations deleted due to missingness)
## AIC: 1808.7
##
## Number of Fisher Scoring iterations: 2
```

```
print(coeftest(fit1, vcov. = vcovCL, cluster = ~subclass))
```

```
##
## z test of coefficients:
##
##                          Estimate  Std. Error  z value  Pr(>|z|)
## (Intercept)              1.404939    0.051281 27.3967    <2e-16 ***
## factor(group)COVID19 0.015755 0.098274   0.1603    0.8726
## ---
## Signif. codes:  0 '***' 0.001 '**' 0.01 '*' 0.05 '.' 0.1 ' ' 1
```

```
dfv2 <- left_join(dff, dfv0, by = "id") %>%
  dplyr::filter(c_rs < median(c_rs, na.rm = TRUE)) %>%
  filter(!is.na(bmi) & !is.na(age_admit) & !is.na(paficat) & !is.na(ma0fe1) &
  ↪  !is.na(charlson_mv)   &
           !is.na(sofa_calc) & !is.na(race2) & !is.na(ethnicity2))
```

© 2023 Nolley EP et al. *JAMA Network Open.*

```r
set.seed(443527)
v2.out <-     matchit(ppnsity.model, data = dfv2, method = "full")
dfv2 <- match.data(v2.out, distance = "prop.score")

dfv2x <- dfv %>%
       dplyr::filter(mvday  <=  6)  %>%
       dplyr::group_by(id)      %>%
       dplyr::summarise(vent_ratio = mean(vent_ratio, na.rm = TRUE)) %>%
       dplyr::right_join(dfv2[, c("id", "weights", "subclass", "group")], by = "id") %>%
       dplyr::select(id, group,vent_ratio , weights, subclass)

dfv2x$group <- factor(dfv2x$group, levels = c("Non-COVID19", "COVID19"))


 fit2 <- glm(vent_ratio ~ factor(group), data = dfv2x,
                     weights = weights)
print(summary(fit2))
```

```
##
## Call:
## glm(formula = vent_ratio ~ factor(group), data = dfv2x, weights = weights)
##
## Deviance Residuals:
## Min                 1Q Median            3Q        Max
##  -2.6820    -0.3885    -0.1716      0.1475    10.9979
##
## Coefficients:
##                            Estimate Std. Error t value Pr(>|t|)
## (Intercept)                1.52303     0.07506  20.290    <2e-16 ***
## factor(group)COVID19 -0.13083      0.09897  -1.322      0.187
## ---
## Signif. codes:  0 '***' 0.001 '**' 0.01 '*' 0.05 '.' 0.1 ' ' 1
##
## (Dispersion parameter for gaussian family taken to be 0.9111743)
##
##     Null deviance: 386.11  on 423  degrees of freedom
## Residual deviance: 384.52  on 422  degrees of freedom
##   (79 observations deleted due to missingness)
## AIC: 1342.1
##
## Number of Fisher Scoring iterations: 2
```

```r
print(coeftest(fit2, vcov. = vcovCL, cluster = ~subclass))
```

```
##
## z test of coefficients:
##
##                            Estimate Std. Error z value Pr(>|z|)
## (Intercept)                1.52303    0.12421  12.262    <2e-16 ***
## factor(group)COVID19 -0.13083  0.15020  -0.871     0.3837
## ---
## Signif. codes:  0 '***' 0.001 '**' 0.01 '*' 0.05 '.' 0.1 ' ' 1
```

```
dat1 = subset(dat, dat$group == "COVID19")
dat2 = subset(dat, dat$group == "Non-COVID19")
dat1$mvdate = as.Date(dat1$mvdate)
dat2$mvdate = as.Date(dat2$mvdate)

df.man1 = NULL
for(i in unique(dat1$id)){
  dfxx = dat1 %>%
    dplyr::filter(id == i) %>%
    dplyr::select(id, mvdate, mechvent) %>%
    dplyr::mutate(date = mvdate)

  id = rep(i, 90)
  date =  seq(as.Date(df$hospdate[df$id == i]),
              as.Date(df$hospdate[df$id == i])+89, "days")

  day = c(0:89)
  death = rep(as.Date(df.daily$date_death[df.daily$id == i]), 90)
  #discharge = rep(as.Date(df$date_discharge[df.daily$id == i]), 90)    ##### It makes the
   ↪ 40 in hospital patient into group == NA >>>>>>>>>>>>>
  discharge = rep(as.Date(df.daily$date_discharge[df.daily$id == i]), 90)
  df.temp = data.frame(id, date, day, death, discharge)

  dfyy = left_join(data.frame(id, date, day, death, discharge), dfxx, by = c("id",
 ↪  "date"))

  dfyy$group = NA
  dfyy$group = replace(dfyy$group, (dfyy$mechvent==0|is.na(dfyy$mechvent)) &
 ↪dfyy$date<=dfyy$discharge & (dfyy$date<=dfyy$death | is.na(dfyy$death)) , 0)
  dfyy$group = replace(dfyy$group, dfyy$mechvent==1 & dfyy$date<=dfyy$discharge &
 ↪  (dfyy$date<=dfyy$death | is.na(dfyy$death)) , 1)
  dfyy$group = replace(dfyy$group, is.na(dfyy$mechvent) & dfyy$date>dfyy$discharge &
 ↪  (dfyy$discharge<=dfyy$death | is.na(dfyy$death)) , 2)
  dfyy$group = replace(dfyy$group, is.na(dfyy$mechvent) & dfyy$date>dfyy$discharge &
 ↪  (dfyy$discharge == dfyy$death) , 3)

  df.man1 = rbind(df.man1, dfyy)
}


df.man2 = NULL
for(i in unique(dat2$id)){
  dfxx = dat2 %>%
    dplyr::filter(id == i) %>%
    dplyr::select(id, mvdate, mechvent) %>%
    dplyr::mutate(date = mvdate)

  id = rep(i, 90)
  date =  seq(as.Date(df$hospdate[df$id == i]),
              as.Date(df$hospdate[df$id == i])+89, "days")

  day = c(0:89)
  death = rep(as.Date(df.daily$date_death[df.daily$id == i]), 90)
```

© 2023 Nolley EP et al. *JAMA Network Open.*

```r
  discharge = rep(as.Date(df.daily$date_discharge[df.daily$id == i]), 90)
  df.temp = data.frame(id, date, day, death, discharge)

  dfyy = left_join(data.frame(id, date, day, death, discharge), dfxx, by = c("id",
↪   "date"))

  dfyy$group = NA
  dfyy$group  =  replace(dfyy$group,  (dfyy$mechvent==0|is.na(dfyy$mechvent))  &
↪dfyy$date<=dfyy$discharge & (dfyy$date<=dfyy$death | is.na(dfyy$death)) , 0)
  dfyy$group = replace(dfyy$group, dfyy$mechvent==1 & dfyy$date<=dfyy$discharge &
↪   (dfyy$date<=dfyy$death | is.na(dfyy$death)) , 1)
  dfyy$group  =  replace(dfyy$group,  is.na(dfyy$mechvent)  &  dfyy$date>dfyy$discharge  &
↪   (dfyy$discharge<=dfyy$death | is.na(dfyy$death)) , 2)
  dfyy$group  =  replace(dfyy$group,  is.na(dfyy$mechvent)  &  dfyy$date>dfyy$discharge  &
↪   (dfyy$discharge == dfyy$death) , 3)

  df.man2 = rbind(df.man2, dfyy)
}

detach("package:plyr")

df.manhtn1 = df.man1 %>%
  distinct() %>%
  dplyr::group_by(id) %>%
  mutate(free = sum(group == 0)) %>%
  mutate(vent = sum(group == 1)) %>%
  mutate(home = sum(group == 2)) %>%
  mutate(dead = sum(group == 3))

df.manhtn2 = df.man2 %>%
  distinct() %>%
  group_by(id) %>%
  mutate(free = sum(group == 0)) %>%
  mutate(vent = sum(group == 1)) %>%
  mutate(home = sum(group == 2)) %>%
  mutate(dead = sum(group == 3))
library(plyr)

df.manhtn1$id = factor(df.manhtn1$id, levels=unique(as.character(df.manhtn1$id)))
df.manhtn2$id = factor(df.manhtn2$id, levels=rev(unique(as.character(df.manhtn2$id))))

df.manhtn1x = df.manhtn1
df.manhtn2x = df.manhtn2

df.manhtn1x$group = "COVID19"
df.manhtn2x$group = "Non-COVID19"

dd = rbind(df.manhtn1x, df.manhtn2x)
write.csv(dd, "dd.csv")
write.csv(df.man1, "df.man1.csv")
write.csv(df.man2, "df.man2.csv")
write.csv(df.manhtn1, "df.manhtn1.csv")
write.csv(df.manhtn2, "df.manhtn2.csv")
```

© 2023 Nolley EP et al. *JAMA Network Open.*

```
df.man1 = read.csv("df.man1.csv")
df.man2 = read.csv("df.man2.csv")
df.manhtn1 = read.csv("df.manhtn1.csv")
df.manhtn2 = read.csv("df.manhtn2.csv")
dd = read.csv("dd.csv")

df.man1 = df.man1 %>% dplyr::filter(!(id %in% stillhosp))
df.man2 = df.man2 %>% dplyr::filter(!(id %in% stillhosp))
df.manhtn1 = df.manhtn1 %>% dplyr::filter(!(id %in% stillhosp))
df.manhtn2 = df.manhtn2 %>% dplyr::filter(!(id %in% stillhosp))
dd = dd %>% dplyr::filter(!(id %in% stillhosp))

ddd = dd %>%
  dplyr::select(id, dead, home, vent, group) %>%
  dplyr::mutate(dead = ifelse(dead > 0, 1, 0),
                home = ifelse(home > 0, 1, 0),
                vent = ifelse(vent > 89, 1, 0)) %>%
  distinct()

# dtest = df[df$id %in% df$id[!(df$id %in% ddd$id)] ,]

dxx = df %>% dplyr::select(id, mvdate) %>% dplyr::rename(mvfirst = mvdate)
dxxx = full_join(dd, dxx, by = "id") %>%
  dplyr::filter(as.Date(date) < as.Date(mvfirst)) %>%
  dplyr::mutate(free_before = 1) %>%
  dplyr::group_by(group, id) %>%
  dplyr::summarise(n = n())

mean(dxxx$n[dxxx$group == "COVID19"], na.rm=TRUE)
```

```
## [1] 4.06
```

```
ci(dxxx$n[dxxx$group == "COVID19"], na.rm=TRUE)
```

```
##   Estimate   CI lower   CI upper  Std. Error
##   4.060000   3.769509   4.350491   0.147853
```

```
mean(dxxx$n[dxxx$group == "Non-COVID19"], na.rm=TRUE)
```

```
## [1] 3.377176
```

```
ci(dxxx$n[dxxx$group == "Non-COVID19"], na.rm=TRUE)
```

```
##   Estimate   CI lower   CI upper  Std. Error
## 3.3771760  3.1154862  3.6388658  0.1332045
```

```
t.test(dxxx$n ~ dxxx$group)
```

© 2023 Nolley EP et al. *JAMA Network Open.*

```
##
##  Welch Two Sample t-test
##
## data:  dxxx$n by dxxx$group
## t = 3.4311, df = 1000.4, p-value = 0.0006256
## alternative hypothesis: true difference in means is not equal to 0
## 95 percent confidence interval:
##  0.2923041 1.0733438
## sample estimates:
##     mean in group COVID19 mean in group Non-COVID19
##                  4.060000                  3.377176
```

```
freebefore.case <- sprintf("%3.1f",round(mean(dxxx$n[dxxx$group == "COVID19"],
    na.rm=TRUE), 1))
freebefore.case.lb <- sprintf("%3.1f",round(ci(dxxx$n[dxxx$group == "COVID19"],
    na.rm=TRUE)[2], 1))
freebefore.case.ub <- sprintf("%3.1f",round(ci(dxxx$n[dxxx$group == "COVID19"],
    na.rm=TRUE)[3], 1))

freebefore.ctrl <- sprintf("%3.1f",round(mean(dxxx$n[dxxx$group == "Non-COVID19"],
    na.rm=TRUE), 1))
freebefore.ctrl.lb <- sprintf("%3.1f",round(ci(dxxx$n[dxxx$group == "Non-COVID19"],
    na.rm=TRUE)[2], 1))
freebefore.ctrl.ub <- sprintf("%3.1f",round(ci(dxxx$n[dxxx$group == "Non-COVID19"],
    na.rm=TRUE)[3], 1))

freebefore.pval <- t.test(dxxx$n ~ dxxx$group)$p.value

message("COVID19 patients intubated after admission:")
print(paste0(freebefore.case, " (", freebefore.case.lb, "-", freebefore.case.ub, ")"))
```

```
## [1] "4.1 (3.8-4.4)"
```

```
message("Non-COVID19 patients intubated after admission:")
print(paste0(freebefore.ctrl, " (", freebefore.ctrl.lb, "-", freebefore.ctrl.ub, ")"))
```

```
## [1] "3.4 (3.1-3.6)"
```

```
### Ventilator free days
temp.df = NULL
for(i in unique(dd$id)){
  dd$mvdate = as.Date(dd$mvdate)
  vv = dd %>% filter(id == i)
  mvdate = min(vv$mvdate, na.rm = TRUE)
  t.eight = mvdate + 28
  maxmv = max(vv$mvdate[vv$mechvent == 1], na.rm = TRUE)

  vv$vfd = 28 - as.numeric(maxmv - mvdate)
  vv$vfd = replace(vv$vfd, maxmv > t.eight, 0)
  vv$vfd = replace(vv$vfd, !is.na(vv$death) & (unique(vv$death) <= t.eight), 0)

  temp.df = rbind(temp.df, vv)
```

© 2023 Nolley EP et al. *JAMA Network Open.*

```r
}

temp.dff = temp.df %>% dplyr::select(id, vfd) %>% distinct()
dff = left_join(dff, temp.dff, by = "id")
df.matched = left_join(df.matched, temp.dff, by = "id")

#chisq.test(q$dead > 0, q$group)
### Hospital free days
d = df %>%
  dplyr::select(id, hospdate, date_discharge, date_death)

d$date_discharge = replace(d$date_discharge, d$date_discharge == d$date_death, NA)
d$date_death = replace(d$date_death, d$date_discharge < d$date_death, NA)

d$deathday = as.Date(d$date_death) - as.Date(d$hospdate)
d$deathday = replace(d$deathday, d$deathday>90, NA)

d$disday = as.Date(d$date_discharge) - as.Date(d$hospdate)

d$hfd = NA
d$hfd = ifelse(d$disday > 60, 0, d$hfd)
d$hfd = ifelse(!is.na(d$deathday), 0, d$hfd)
d$hfd = ifelse(d$disday < 60, 60-d$disday, d$hfd)

dff = left_join(dff, d, by = "id")
df.matched = left_join(df.matched, d, by = "id")

##################
###    Table1    ###
##################
fun.tab1 = function(datain){
  tab.cont = NULL
  for(i in c("age_admit", "bmi", "l_wbc_admit", "l_o2flow_admit", "sofa_calc",
   ↪ "charlson_mv", "pafi_mv0", "vfd",
             "l_temp_admit", "h_temp_admit", "hfd")){
    cv.m = mean(as.numeric(datain[[i]][datain$group == "COVID19"]), na.rm = TRUE)
    ncv.m = mean(as.numeric(datain[[i]][datain$group == "Non-COVID19"]), na.rm = TRUE)
    cv.sd = sd(as.numeric(datain[[i]][datain$group == "COVID19"]), na.rm = TRUE)
    ncv.sd = sd(as.numeric(datain[[i]][datain$group == "Non-COVID19"]), na.rm = TRUE)

    cv = paste0(sprintf("%3.1f", round(cv.m, 1)), " (", sprintf("%3.1f", round(cv.sd,
 ↪ 1)), ")")
    ncv = paste0(sprintf("%3.1f", round(ncv.m, 1)), " (", sprintf("%3.1f", round(ncv.sd,
 ↪ 1)), ")")

    p = sprintf("%3.4f",t.test(datain[[i]] ~ datain$group)$p.value)

    tab.cont = rbind(tab.cont, c(i, cv, ncv, p))
  }


  tab.cat = NULL
  for(i in c("ma0fe1", "race2", "ethnicity2", "diabetes", "asthma", "copd", "cvd", "ckd",
   ↪ "immunosuppressed",
```

© 2023 Nolley EP et al. *JAMA Network Open.*

```
                "current_smoker", "former_smoker", "fever_l", "fever_h", "curb65", "viral",
                 ↪  "bacterial",
               "fungal", "pneumonia.with.unspecified.organism", "aspiration", "ards",
                 ↪  "lowflow", "hfnc",
               "nppv", "dead", "home", "vent", "ards")){
    datain$temp = NULL
    datain$temp = as.factor(datain[[i]])
    yy = datain %>%
      tabyl(temp, group) %>%
      adorn_percentages("col") %>%
      adorn_pct_formatting(digits = 1) %>%
      adorn_ns(position = "front")


    p = substr(format(chisq.test(datain$temp, datain$group)$p.value , scientific = F), 1,
 ↪  6)
    yy$temp = paste0(i , " (", yy$temp, ")")
    yy = cbind(yy, p)
    tab.cat = rbind(tab.cat, yy)
  }
  colnames(tab.cont) <- colnames(tab.cat) <- c("Variable", "COVID19", "Non-COVID19",
   ↪  "P-value")
  tab1 = rbind(tab.cont, tab.cat)
  return(tab1)
}

dff = left_join(dff, ddd, by = c("id", "group"))
tb1 = as.data.frame(fun.tab1(dff))
```

```
###########################
###    Table 1 matched    ###
###########################
fun.tab1.match = function(datain){
  tab.cont = NULL
  for(i in c("age_admit", "bmi", "l_wbc_admit", "l_o2flow_admit", "sofa_calc",
   ↪  "charlson_mv", "pafi_mv0", "vfd",
               "l_temp_admit", "h_temp_admit", "hfd")){

frmla = paste0(i, " ~ as.factor(group)")

fit <- lm(frmla, data = df.matched, weights = weights)
xx = tidy(coeftest(fit, vcov. = vcovCL, cluster = ~subclass), conf.int=TRUE)

case.m = xx$estimate[xx$term == "(Intercept)"]
sd = summary(fit)$sigma
case = paste0(sprintf("%3.1f",case.m), " (", sprintf("%3.1f",sd), ")")

ctrl.m = sprintf("%3.1f", case.m + xx$estimate[xx$term == "as.factor(group)Non-COVID19"])
ctrl = paste0(ctrl.m, " (", sprintf("%3.1f",sd), ")")

p = sprintf("%3.4f", xx$p.value[xx$term == "as.factor(group)Non-COVID19"])
tab.cont = rbind(tab.cont, c(i, case, ctrl, p))
}
```

© 2023 Nolley EP et al. *JAMA Network Open.*

```
tab.cat  =  NULL
for(r in c("ma1fe2", "race2", "ethnicity2", "diabetes", "asthma", "copd", "cvd", "ckd",
 ↪  "immunosuppressed",
               "current_smoker", "former_smoker", "fever_l", "fever_h", "curb65", "viral",
                ↪  "bacterial",  "fungal",
              "pneumonia.with.unspecified.organism", "aspiration", "vap", "ards", "lowflow",
                ↪  "hfnc", "nppv", "ards",
              "dead", "home", "vent")){

frmla2 = paste0("as.factor(",r, ")", " ~ as.factor(group)")
fit2 <- glm(frmla2, data = df.matched, weights = weights,
                 family = quasibinomial(link = "logit"))

yy = tidy(coeftest(fit2, vcov. = vcovCL, cluster = ~subclass), conf.int = TRUE,
 ↪  exponentiate = FALSE)

case.cat = (exp(yy$estimate[1]) / (1+exp(yy$estimate[1])))*100
ctrl.cat = (exp(yy$estimate[1] + yy$estimate[2]) / (1+exp(yy$estimate[1] +
 ↪  yy$estimate[2])))*100

n = glance(fit2)$nobs
p2 = sprintf("%3.4f", yy$p.value[yy$term == "as.factor(group)Non-COVID19"])

tab.cat = rbind(tab.cat,
                   c(r, paste0(sprintf("%3.1f", case.cat), "%", "(", n, ")"),
                     paste0(sprintf("%3.1f", ctrl.cat), "%", "(", n, ")"), p2))
}

colnames(tab.cont) <- colnames(tab.cat) <- c("Variable", "COVID19", "Non-COVID19",
 ↪  "P-value")
tab1 = rbind(tab.cont, tab.cat)
colnames(tab1) <- c("Variable", "COVID19", "Non-COVID19", "P-value")
return(tab1)
}

df.matched$ma1fe2 = ifelse(df.matched$ma0fe1==0, 2, df.matched$ma0fe1)
df.matched = left_join(df.matched, ddd, by = c("id", "group"))

tb1_matched    =    as.data.frame(fun.tab1.match(df.matched))
```

```
rwnames = c("Demographics", "Age in years, mean (SD)", "% males (n)", "Body mass index in
 ↪  kg/m2, mean (SD)", "% Non-white race (n)", "% Hispanic ethnicity (n)",
 ↪  "Comorbidities", "% Diabetes (n)", "% Cardiovascular disease (n)", "% COPD (n)", "%
 ↪  Chronic kidney disease (n)", "% Immunosuppression (n)", "% Current smoker (n)", "%
 ↪  Former smoker (n)", "Charlson comorbidity index, mean (SD)", "Clinical parameters on
 ↪  admission", "Lowest white blood cell count in cells/mm3, mean (SD)", "Highest
 ↪  temperature in oC, mean (SD)", "Lowest temperature in oC, mean (SD)", "Modes of
 ↪  respiratory support prior to intubation", "Low-flow oxygen, % (n)", "High-flow
 ↪  oxygen, % (n)",
"NIPPV ", "Clinical parameters on first day of mechanical ventilation", "SOFA score, mean
 ↪  (SD)", "PaO2/FiO2 , mean (SD)", "Clinical outcomes", "Overall hospital mortality, %
 ↪  (n)")
```

© 2023 Nolley EP et al. *JAMA Network Open.*

```
rowlist1 <- c("age_admit", "ma0fe1 (0)", "bmi", "race2 (1)", "ethnicity2 (1)",
                "diabetes (1)", "cvd (1)", "copd (1)", "ckd (1)", "immunosuppressed (1)",
                "current_smoker (1)", "former_smoker (1)", "charlson_mv",
                "l_wbc_admit", "h_temp_admit", "l_temp_admit",
                "lowflow (1)", "hfnc (1)", "nppv (1)",
                "sofa_calc", "pafi_mv0", "dead (1)")

rowlist2 <- c("age_admit", "ma1fe2", "bmi", "race2", "ethnicity2",
                "diabetes", "cvd", "copd", "ckd", "immunosuppressed",
                "current_smoker", "former_smoker", "charlson_mv",
                "l_wbc_admit", "h_temp_admit", "l_temp_admit",
                "lowflow", "hfnc", "nppv",
                "sofa_calc", "pafi_mv0", "dead")

lst1 <- NULL
rwlist1 <- for(v1 in rowlist1){lst1 <- c(lst1, which(tb1$Variable %in% v1))}
lst2 <- NULL
rwlist2 <- for(v2 in rowlist2){lst2 <- c(lst2, which(tb1_matched$Variable %in% v2))}


tbl1 = tb1[lst1, c(2,3,4)]
tbl2 = tb1_matched[lst2, c(2,3,4)]
tbl = cbind(tbl1, tbl2)
tbl[[3]] = ifelse(tbl[[3]] == "0.0000", "<0.001", tbl[[3]])
tbl[[6]] = ifelse(tbl[[6]] == "0.0000", "<0.001", tbl[[6]])

blank = rep("", 6)
tbl = rbind(blank, tbl[c(1:5),],
             blank, tbl[c(6:13),],
             blank, tbl[c(14:16),],
             blank, tbl[c(17:19),],
             blank, tbl[c(20,21),],
             blank, tbl[c(22),])

rownames(tbl) = rwnames

tbl[[4]] = ifelse(grepl("%", tbl[[4]]) == "TRUE", str_extract(tbl[[4]], "^(.*?%)"),
 ↪ tbl[[4]])
tbl[[5]] = ifelse(grepl("%", tbl[[5]]) == "TRUE", str_extract(tbl[[5]], "^(.*?%)"),
 ↪ tbl[[5]])

tbl[[3]] = ifelse(grepl("0.000", tbl[[3]]) == "TRUE", "<0.01",
        ifelse(tbl[[3]] != "<0.001" & tbl[[3]] != "<0.01", substr(tbl[[3]], 1, 4),
         ↪ tbl[[3]]))

tbl[[6]] = ifelse(grepl("0.000", tbl[[6]]) == "TRUE", "<0.01",
        ifelse(tbl[[6]] != "<0.001" & tbl[[6]] != "<0.01", substr(tbl[[6]], 1, 4),
         ↪ tbl[[6]]))


kable(tbl, "latex", booktabs = TRUE,
      col.names = c("COVID-19", "Non-COVID-19", "p-value", "COVID-19", "Non-COVID-19",
       ↪ "p-value")) %>%
```

© 2023 Nolley EP et al. *JAMA Network Open.*

```
add_header_above(c("", "Before matching"= 3, "After optimal full matching" = 3)) %>%
add_indent(c(2:6, 8:15, 17:19, 21:23, 25, 26, 28),1) %>%
kable_classic(latex_options = c("scale_down", "HOLD_position"))
```

	Before matching			After optimal full matching		
	COVID-19	Non-COVID-19	p-value	COVID-19	Non-COVID-19	p-value
Demographics						
Age in years, mean (SD)	61.8 (15.3)	60.9 (15.8)	0.22	61.9 (15.7)	62.4 (15.7)	0.75
% males (n)	442 (61.5%)	586 (52.0%)	<0.001	61.7%	69.0%	0.01
Body mass index in kg/m2, mean (SD)	32.0 (9.1)	29.7 (11.6)	<0.001	32.0 (15.6)	33.1 (15.6)	0.61
% Non-white race (n)	460 (64.0%)	459 (40.7%)	<0.001	65.0%	63.3%	0.56
% Hispanic ethnicity (n)	127 (17.7%)	28 (2.5%)	<0.001	18.1%	20.5%	0.47
Comorbidities						
% Diabetes (n)	164 (22.8%)	165 (14.6%)	<0.001	23.0%	15.6%	0.02
% Cardiovascular disease (n)	63 (8.8%)	141 (12.5%)	0.01	8.6%	10.3%	0.36
% COPD (n)	89 (12.4%)	328 (29.1%)	<0.001	12.3%	23.6%	<0.001
% Chronic kidney disease (n)	227 (31.6%)	369 (32.7%)	0.63	31.6%	24.5%	0.03
% Immunosuppression (n)	43 (6.0%)	47 (4.2%)	0.09	6.2%	1.6%	<0.001
% Current smoker (n)	41 (5.7%)	253 (22.4%)	<0.001	5.7%	26.2%	<0.001
% Former smoker (n)	230 (32.0%)	341 (30.3%)	0.46	32.7%	26.1%	0.04
Charlson comorbidity index, mean (SD)	2.7 (2.7)	3.8 (3.1)	<0.001	2.7 (2.6)	2.9 (2.6)	0.55
Clinical parameters on admission						
Lowest white blood cell count in cells/mm3, mean (SD)	8.6 (5.3)	11.4 (11.2)	<0.001	8.4 (7.2)	12.0 (7.2)	<0.001
Highest temperature in oC, mean (SD)	100.3 (1.8)	100.0 (1.7)	0.00	100.3 (1.8)	99.9 (1.8)	0.04
Lowest temperature in oC, mean (SD)	96.9 (5.1)	96.3 (5.5)	0.01	97.0 (4.2)	96.6 (4.2)	0.15
Modes of respiratory support prior to intubation						
Low-flow oxygen, % (n)	474 (65.9%)	628 (55.7%)	<0.001	67.1%	56.4%	0.02
High-flow oxygen, % (n)	391 (54.4%)	157 (13.9%)	<0.001	55.8%	14.2%	<0.001
NIPPV	116 (16.1%)	421 (37.4%)	<0.001	16.4%	34.3%	<0.001
Clinical parameters on first day of mechanical ventilation						
SOFA score, mean (SD)	15.4 (2.4)	14.5 (2.4)	<0.001	15.4 (2.3)	15.2 (2.3)	0.28
PaO2/FiO2 , mean (SD)	180.0 (104.7)	245.7 (142.5)	<0.001	180.1 (98.4)	188.5 (98.4)	0.25
Clinical outcomes						
Overall hospital mortality, % (n)	295 (41.0%)	385 (34.2%)	0.00	40.2%	38.0%	0.67

Figure 1: Time-to-death and to time-to-discharge alive for patients with COVID-19 and and non-COVID-19

```
d1 = df.manhtn1 %>% filter(dead > 0) %>% group_by(id) %>% arrange(desc(dead), desc(free))
d2 = df.manhtn2 %>% filter(dead > 0) %>% group_by(id) %>% arrange(desc(dead), desc(free))
d3 = df.manhtn1 %>% filter(dead == 0) %>% group_by(id) %>% arrange(desc(home),
  desc(free))
d4 = df.manhtn2 %>% filter(dead == 0) %>% group_by(id) %>% arrange(desc(home),
  desc(free))

d1$id = factor(d1$id, levels=unique(as.character(d1$id)))
d2$id = factor(d2$id, levels=rev(unique(as.character(d2$id))))
d3$id = factor(d3$id, levels=unique(as.character(d3$id)))
d4$id = factor(d4$id, levels=rev(unique(as.character(d4$id))))

fun.manhattalplot = function(datain, pos, xtitle, ytitle, ptitle, col){

  med = median(datain$vent, na.rm = TRUE)

  p =
    ggplot(data = datain,
```

© 2023 Nolley EP et al. *JAMA Network Open.*

```
            aes(y = as.numeric(day),
                x = as.factor(id),
                fill = factor(group))) +
    geom_tile() +
    ggtitle(ptitle)  +
    #geom_hline(yintercept = med, color = "white", size = 1.2, linetype = "dashed") +
    theme(plot.title = element_text(size = 24, hjust = 0.5),
          panel.background = element_rect(fill = "transparent"),
          plot.background = element_rect(fill = "transparent", color = NA),
          legend.position = "none",
          axis.ticks.x = element_blank(),
          axis.ticks.length = unit(0.2, "cm"),
          axis.title = element_text(size = 21),
          axis.text = element_text(size = 14),
          axis.text.x = element_blank())

  if(pos == 1){p = p +
    scale_fill_manual(values = c("grey", "black", col))+
    scale_y_continuous(ytitle, expand = c(0,0), limits = c(0,90), breaks = c(1, 30, 60,
↪   89),
    sec.axis = sec_axis(~., breaks = c(1, 30, 60, 89), labels = c(" 0", " 30", " 60", "
      ↪  90")))+
    scale_x_discrete("", expand = c(0,0)) +
    theme(plot.margin = unit(c(0.8, -0.4, -0.2, 0.2), "cm"),
          axis.text.y.left = element_blank(),
          axis.ticks.y.left = element_blank(),
          axis.text.y.right = element_text(hjust = 0.5))
    #annotate("text", label = round(med, 1), x = -Inf, hjust = -0.5, y = med + 5, color =
      ↪  "white", size = 6)
  }

  if(pos == 2){p = p +
    scale_fill_manual(values = c("grey", "black", col)) +
    scale_y_continuous(ytitle, expand = c(0,0), limits = c(0,90), breaks = c(1, 30, 60,
↪   89),
                       sec.axis = sec_axis(~., breaks = c(1, 30, 60, 89), labels = c("0",
                        ↪  "30", "60", "90")))+
    scale_x_discrete("", expand = c(0,0)) +
    theme(plot.margin = unit(c(0.8, 0, -0.2, -0.2), "cm"),
          axis.text.y.left = element_blank(),
          axis.ticks.y.right = element_blank(),
          axis.text.y.right = element_blank())
    #annotate("text", label = round(med, 1), x = Inf, hjust = 2, y = med + 5, color =
      ↪  "white", size = 6)
  }

  if(pos == 3){p = p +
    scale_fill_manual(values = c("grey", "black", col)) +
    scale_y_reverse(ytitle, expand = c(0,0), limits = c(90,0), breaks = c(1, 30, 60, 89),
    sec.axis = sec_axis(~., breaks = c(1, 30, 60, 89), labels = c(" 0", " 30", " 60", "
      ↪  90")))+
  scale_x_discrete("", expand = c(0,0)) +
  theme(plot.margin = unit(c(0.8, -0.4, -0.2, 0.2), "cm"),
```

© 2023 Nolley EP et al. *JAMA Network Open.*

```
          axis.text.y.left = element_blank(),
          axis.ticks.y.left = element_blank(),
          axis.text.y.right = element_text(hjust = 0.5))
   # annotate("text", label = round(med, 1), x = -Inf, hjust = -0.5, y = med + 5, color =
    ↪  "white", size = 6)
  }

  if(pos == 4){p = p +
    scale_fill_manual(values = c("grey", "black", col)) +
    scale_y_reverse(ytitle, expand = c(0,0), limits = c(90,0), breaks = c(1, 30, 60, 89),
                      sec.axis = sec_axis(~., breaks = c(1, 30, 60, 89), labels = c("0",
                       ↪  "30", "60", "90")))+
    scale_x_discrete("", expand = c(0,0)) +
    theme(plot.margin = unit(c(0.8, 0, -0.2, -0.2), "cm"),
          axis.text.y.left = element_blank(),
          axis.ticks.y.right = element_blank(),
          axis.text.y.right = element_blank())
   # annotate("text", label = round(med, 1), x = Inf, hjust = 2, y = med + 5, color =
    ↪  "white", size = 6)
  }
  return(p)
}

p1 = fun.manhattalplot(d1, pos = 1, "", "Time (days)", "COVID-19", col="red")
p2 = fun.manhattalplot(d2, pos = 2, "", "", "Non COVID-19", col="red")

p3 = fun.manhattalplot(d3, pos = 3, "", "Time (days)", "", col="blue")
p4 = fun.manhattalplot(d4, pos = 4, "", "", "", col="blue")

pp = ggplot(df.manhtn1, aes(x = as.numeric(day), y = as.factor(id), fill =
 ↪factor(group))) +
  geom_tile()+
  scale_fill_manual(values = c("grey", "black", "blue", "red"),
                      labels = c("Ventilator free but in hospital", "Ventilator",
                       ↪  "Discharged alive" ,"Dead")) +
  theme(legend.title = element_blank(), legend.position = "bottom", rect =
 ↪  element_rect(fill = "transparent"))

g <- ggplotGrob(pp + theme(legend.position="bottom",
                            legend.text = element_text(size=20)))$grobs
legend <- g[[which(sapply(g, function(x) x$name) == "guide-box")]]
lheight <- sum(legend$height)
legend$heights[2] = unit(c(0.10), "cm")

g1 = ggplotGrob(p1)
g2 = ggplotGrob(p2)
g3 = ggplotGrob(p3)
g4 = ggplotGrob(p4)

ggdraw()+
  draw_grob(g1, x = 0, y = 0.50, height = 0.53, width = 0.30) +
  draw_grob(g2, x = 0.3, y = 0.50, height = 0.53, width = 0.7) +
  draw_grob(g3, x = 0, y = 0.02, height = 0.53, width = 0.30) +
```

© 2023 Nolley EP et al. *JAMA Network Open.*

```
  draw_grob(g4, x = 0.3, y = 0.02, height = 0.53, width = 0.7) +
  draw_grob(legend, x = 0, y = 0, height = 0.05, width = 1) -> p
p
```

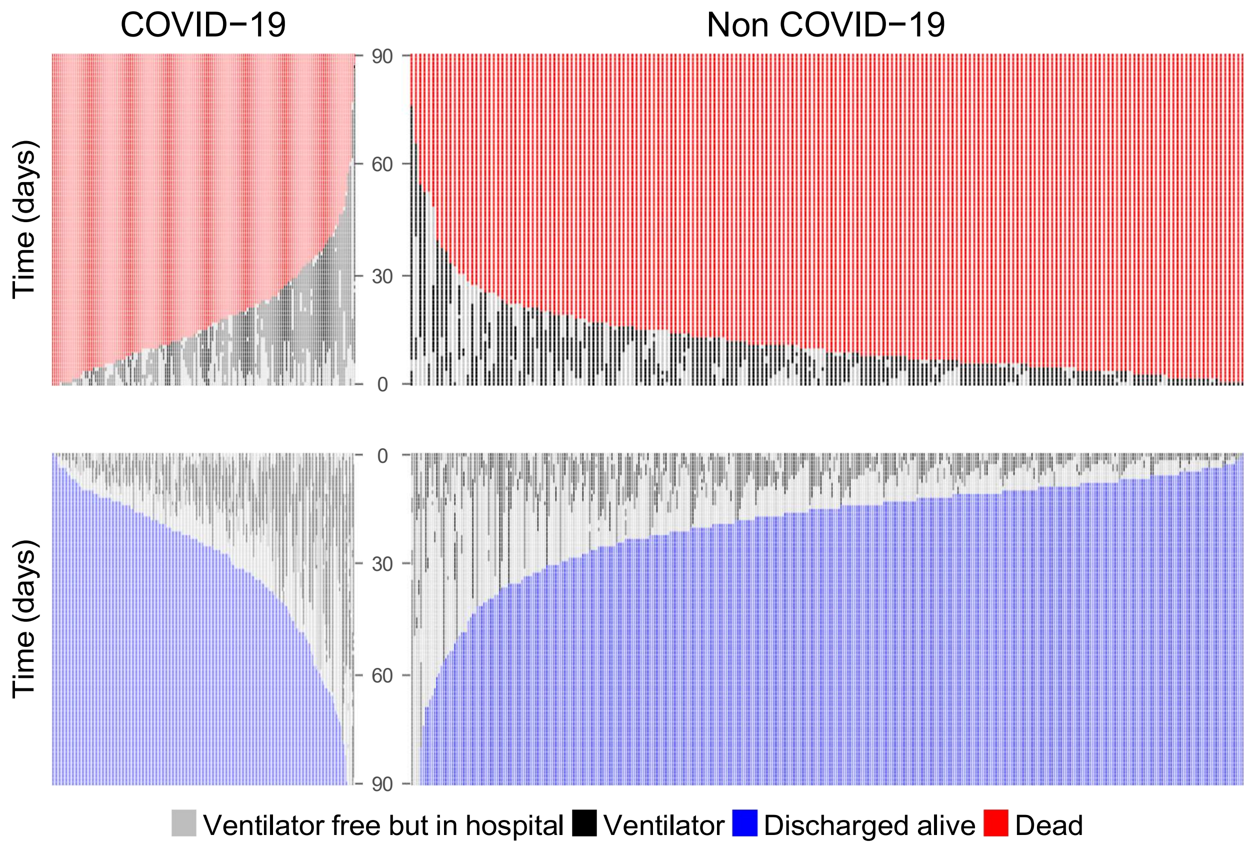


```
ggsave(p, filename = "Figure-1.jpg", dpi = 300, type = "cairo",
       width = 12, height = 7, units = "in")

ggsave(p, filename = "Figure-1.tiff", dpi = 300, type = "cairo",
       width = 12, height = 7, units = "in")

ggsave(p, filename = "Figure-1.pdf", device = "pdf",
       width = 12, height = 7, units = "in")


med.d3 <- median(d3$vent, na.rm = TRUE)
q2.d3 <- quantile(d3$vent, na.rm = TRUE)[2]
q3.d3 <- quantile(d3$vent, na.rm = TRUE)[4]

med.d4 <-median(d4$vent, na.rm = TRUE)
q2.d4 <- quantile(d4$vent, na.rm = TRUE)[2]
q3.d4 <- quantile(d4$vent, na.rm = TRUE)[4]

d3$group2 = "COVID19"
d4$group2 = "Non-COVID19"

d = rbind(d3, d4)
```

© 2023 Nolley EP et al. *JAMA Network Open.*

```
pd3d4 <- format(t.test(vent ~ group2, data = d)$p.value, scientific = FALSE)


message("Median and IQR mechanical ventilation days for the patients with COVID19
  pneumonia who were discharged aslive:")
print(paste0(med.d3, " (", q2.d3, " to ", q3.d3, ")"))
```

```
## [1] "10 (5 to 20)"
```

```
message("Median and IQR mechanical ventilation days for the patients with Non-COVID19
  pneumonia who were discharged aslive:")
print(paste0(med.d4, " (", q2.d4, " to ", q3.d4, ")"))
```

```
## [1] "5 (3 to 10)"
```

```
####################
###    Figure-4    ###
####################

theme_set(theme_bw(base_size = 21))

tab_x = NULL
for(i in 0:6){
  dx1 = dat %>%
    dplyr::filter(mvday == i) %>%
    dplyr::select(id, mvdate, mvday, group) %>%
    left_join(df[,c("id", "date_death", "date_discharge")], by = "id") %>%
    mutate(date_death = as.Date(date_death),
           date_discharge = as.Date(date_discharge))

  dx1$dead = ifelse(dx1$date_death == dx1$mvdate, 1, 0)
  dx1$discharge = ifelse((dx1$date_discharge == dx1$mvdate), 1, 0)
  dx1$discharge = replace(dx1$discharge, dx1$dead == 1, 0)

  a = table(dx1$dead[dx1$group == "COVID19"])[2]
  b = table(dx1$discharge[dx1$group == "COVID19"])[2]

  c = table(dx1$dead[dx1$group == "Non-COVID19"])[2]
  d = table(dx1$discharge[dx1$group == "Non-COVID19"])[2]

  tab_x = rbind(tab_x, c(i, a, b, c, d))
  colnames(tab_x) = c("mvday", "cov_dead", "cov_discharged", "ncov_dead",
    "ncov_discharged")
}
tab_x = data.frame(tab_x)
tab_x$cov_dead = ((cumsum(tab_x$cov_dead))/table(df$group)[1])*100
tab_x$cov_discharged = ((cumsum(tab_x$cov_discharged))/table(df$group)[1])*100

tab_x$ncov_dead = ((cumsum(tab_x$ncov_dead))/table(df$group)[2])*100
tab_x$ncov_discharged = ((cumsum(tab_x$ncov_discharged))/table(df$group)[2])*100
```

© 2023 Nolley EP et al. *JAMA Network Open.*

```
fun.fig3 = function(xvar,
                    groupvar,
                    yvar,
                    y.lab,
                    y.min,
                    y.max,
                    breaks,
                    x.lab,
                    yminl,
                    ymaxl,
                    yminh,
                    ymaxh,
                    coord1,
                    coord2,
                    labl,
                    labh,
                    matched,
                    x1,
                    x2,
                    pprint = NULL,
                    bluline = NULL){

  if(matched == 0){
    dff = read.csv("dat.csv")
    dff$vt = ifelse(dff$vt == 0, NA, dff$vt)
    dff$pdriv = ifelse(dff$pdriv <= 0, NA, dff$pdriv)
    dff$vtpbw = ifelse(dff$vtpbw <= 0 , NA, dff$vtpbw)


    dff$xvar = dff[[xvar]]
    dff$yvar = dff[[yvar]]
    dff$groupvar = dff[[groupvar]]

    frmla = paste0("yvar ~ as.factor(group)")

    bbb = NULL
    for(i in 0:6){
      #message(paste0("Day", i,":"))

      cs = dff$yvar[dff$mvday == i & dff$group == "COVID19"]
      cs.mean = mean(cs, na.rm = TRUE)
      cs.sd = sd(cs, na.rm = TRUE)
      cs.lb = cs.mean - 1.96*cs.sd
      cs.ub = cs.mean + 1.96*cs.sd

      case.m = cs.mean
      case.lb = cs.lb
      case.ub = cs.ub

      ct = dff$yvar[dff$mvday == i & dff$group == "Non-COVID19"]
      ct.mean = mean(ct, na.rm = TRUE)
      ct.sd = sd(ct, na.rm = TRUE)
```

© 2023 Nolley EP et al. *JAMA Network Open.*

```r
        ct.lb = ct.mean - 1.96*ct.sd
        ct.ub = ct.mean + 1.96*ct.sd

        ctrl.m = ct.mean
        ctrl.lb = ct.lb
        ctrl.ub = ct.ub

if(yvar %in% c("c_rs", "vent_ratio")){
        cs = log(dff$yvar[dff$mvday == i & dff$group == "COVID19"])
        cs.mean = mean(cs, na.rm = TRUE)
        cs.sd = sd(cs, na.rm = TRUE)
        cs.lb = cs.mean - 1.96*cs.sd
        cs.ub = cs.mean + 1.96*cs.sd

        case.m = exp(cs.mean)
        case.lb = exp(cs.lb)
        case.ub = exp(cs.ub)

        ct = log(dff$yvar[dff$mvday == i & dff$group == "Non-COVID19"])
        ct.mean = mean(ct, na.rm = TRUE)
        ct.sd = sd(ct, na.rm = TRUE)
        ct.lb = ct.mean - 1.96*ct.sd
        ct.ub = ct.mean + 1.96*ct.sd

        ctrl.m = exp(ct.mean)
        ctrl.lb = exp(ct.lb)
        ctrl.ub = exp(ct.ub)

}

        p = format(t.test(yvar ~ group, data = dff[dff$mvday == i,])$p.value, scientific =
  ↪  F)

if(yvar %in% c("c_rs", "vent_ratio")){
   p = format(tidy(glm(yvar ~ group, data = dff[dff$mvday == 0,], family = gaussian(link =
  ↪  "log")))$p.value, scientific = F)
}

if(i == 0 & !is.null(pprint) & !(yvar %in% c("c_rs", "vent_ratio"))){
   print(t.test(yvar ~ group, data = dff[dff$mvday == 0,]))
   }


if(i == 0 & !is.null(pprint) & (yvar %in% c("c_rs", "vent_ratio"))){
   dff$group2 <- factor(dff$group, levels = c("Non-COVID19", "COVID19"))
   fitxx <- glm(yvar ~ group2, data = dff[dff$mvday == 0,], family = gaussian(link =
  ↪"log"))
   print(summary(fitxx))
   print(exp(coef(fitxx)))
   print(exp(confint(fitxx)))
}

        bbb = rbind(bbb,
```

© 2023 Nolley EP et al. *JAMA Network Open.*

```
                  c("COVID19", i,case.m, case.lb, case.ub),
                  c("Non-COVID19", i, ctrl.m, ctrl.lb, ctrl.ub))
  }

  bbb = data.frame(bbb)
  colnames(bbb) = c("groupvar", "xvar", "mean", "lb", "ub")
  dff <-bbb

  for(b in c("xvar", "mean", "lb", "ub")){
    dff[[b]] = as.numeric(dff[[b]])
  }
}

p =
  ggplot(data = dff,
         aes(group = groupvar)) +
  geom_errorbar(aes(ymin = lb,
                    ymax = ub,
                    x = xvar,
                    color = factor(groupvar)),
                size = 1,
                position = position_dodge(width = 0.5),
                width = 0) +

   geom_pointline(aes(x  =  xvar,
                      y = mean,
                      color = factor(groupvar)),
                  position = position_dodge(width = 0.5),
                  size = 10,
                  shape = 18,
                  distance = 5)

for(v in 0:6){
  distance = sqrt((tab_x$ncov_dead[v+1]))/5
  distance2 = sqrt((tab_x$ncov_discharged[v+1]))/5

  distance3 = sqrt((tab_x$cov_dead[v+1]))/5
  distance4 = sqrt((tab_x$cov_discharged[v+1]))/5

  p = p +
    annotation_custom(grob = circleGrob(r = unit(distance, "npc"),
                                        gp = gpar(col = "black",
                                                  lty = 3, fill = "black")),
                      xmin = v-(distance/2),
                      xmax = v+distance,
                      ymin = yminl,
                      ymax = ymaxl) +

     annotation_custom(grob = circleGrob(r = unit(distance3, "npc"),
                                         gp = gpar(col = "grey",
                                                   lty = 3, fill = "grey")),
                       xmin = v-distance3,
                       xmax = v + (distance3/2),
```

© 2023 Nolley EP et al. *JAMA Network Open.*

```
                        ymin = yminl,
                        ymax = ymaxl) +

      annotation_custom(grob = circleGrob(r = unit(distance2, "npc"),
                                          gp = gpar(col = "black",
                                                    lty = 3,
                                                    fill = "black")),
                        xmin = v - (distance2/2),
                        xmax = v + distance2,
                        ymin = yminh,
                        ymax = ymaxh) +

      annotation_custom(grob = circleGrob(r = unit(distance4, "npc"),
                                          gp = gpar(col = "grey",
                                                    lty = 3,
                                                    fill = "grey")),
                        xmin = v - distance4,
                        xmax = v + (distance4/2),
                        ymin = yminh,
                        ymax = ymaxh)
  }

  p = p +

    scale_y_continuous(y.lab,
                       #limits = c(y.min, y.max),
                       breaks = seq(y.min, y.max, breaks),
                       expand = c(0.2, 0.2)) +
    scale_x_continuous(x.lab,
                       breaks = seq(0, 6, 1),
                       expand = c(0.05, 0.05)) +
    scale_color_manual(name = "x", values = c("grey", "black")) +
    scale_linetype_manual(name = "x", values = c("solid", "solid")) +
    coord_cartesian(ylim = c(coord1, coord2))+
    lemon::annotate_y_axis("Dead ", y = (yminl + ymaxl)/2, side = "left", print_value =
 ↪  FALSE, fontface = "bold", color = "black") +
    lemon::annotate_y_axis("Extubated ", y = (yminh + ymaxh)/2 , side = "left",
 ↪  print_value = FALSE, fontface = "bold", color = "black") +
    theme_bw(base_size = 21) +
   # theme_bw() +
    theme(legend.title = element_blank(),
          legend.position = "none",
          axis.title.x = element_text(size = 21),
          axis.title.y = element_text(size = 21, margin = margin(t=0, r=30, b=0, l=0)),
          axis.text = element_text(size = 18),
          legend.text = element_text(size = 15))

if(is.null(bluline) == "FALSE"){
  for(b in bluline){
    p = p +
      annotate("segment", x = b-0.2, xend = b-0.05, y = y.max+(y.max-y.min)/6.5, yend =
 ↪  y.max+(y.max-y.min)/5.65, color = "steelblue") +
      annotate("segment", x = b-0.2, xend = b-0.05, y = y.max+(y.max-y.min)/7, yend =
 ↪  y.max+(y.max-y.min)/6, color = "steelblue")+
```

© 2023 Nolley EP et al. *JAMA Network Open.*

```
      annotate("segment", x = b+0.05, xend = b+0.2, y = y.max+(y.max-y.min)/6.5, yend =
↪  y.max+(y.max-y.min)/5.65, color = "steelblue") +
      annotate("segment", x = b+0.05, xend = b+0.2, y = y.max+(y.max-y.min)/7, yend =
↪  y.max+(y.max-y.min)/6, color = "steelblue")
  }
}

  return(p)
}

### Legend
#dff = read.csv("dat.csv")

p =    ggplot(data = dat %>%
                  dplyr::filter(mvday <= 6) %>%
                  dplyr::select(id, mvday, group, pplat),
                aes(group = group)) +

  geom_pointline(aes(x = mvday,
                     y = pplat,
                    color = factor(group)),
                 position = position_dodge(width = 0.5),
                 size = 10,
                 shape = 18,
                 distance = 5) +
  scale_color_manual(values = c("grey", "black")) +
  theme_bw()+
  theme(legend.title = element_blank())
g <- ggplotGrob(p + theme(legend.position = "bottom",
                          legend.text = element_text(size = 18)))$grobs
legend <- g[[which(sapply(g, function(x) x$name) == "guide-box")]]
lheight <- sum(legend$height)
legend$heights[2] = unit(c(2), "cm")

distance = sqrt(5)/5
distance2 = sqrt(10)/5

distance3 = sqrt(15)/5
distance4 = sqrt(20)/5

### crs
a = 10
b = 80
gap = (b-a)/20

#gap = ((b-a)/17.5)

p3 =
  fun.fig3("mvday", "group", "c_rs",
           bquote(Respiratory~compliance~ml*"/(cm "~H[2]*0*")"),
           a, b, 10, "Days on ventilator",
           a-gap*4,
           a-gap*2.6,
```

© 2023 Nolley EP et al. *JAMA Network Open.*

```
            b+gap*2.6,
            b+gap*4,
            coord1 = a-gap*2 ,
            coord2 = b+gap*2 ,
            labl = 0.217,
            labh = 0.917,
            matched = 0,
            x1 = 0.130,
            x2 = 0.080,
            pprint = 1) +
  annotate("text", x = -Inf, y = Inf, hjust = -1, vjust = 2, size = 9, label = "E")
```

```
##
## Call:
## glm(formula = yvar ~ group2, family = gaussian(link = "log"),
##     data = dff[dff$mvday == 0, ])
##
## Deviance Residuals:
##     Min       1Q   Median       3Q      Max
## -34.149  -11.009   -4.579    4.901  243.991
##
## Coefficients:
##                Estimate Std. Error t value Pr(>|t|)
## (Intercept)     3.45454    0.03001 115.108  < 2e-16 ***
## group2COVID19  0.12923    0.03834   3.371 0.000775 ***
## ---
## Signif. codes:  0 '***' 0.001 '**' 0.01 '*' 0.05 '.' 0.1 ' ' 1
##
## (Dispersion parameter for gaussian family taken to be 441.9279)
##
##     Null deviance: 485468  on 1088  degrees of freedom
## Residual deviance: 480332  on 1087  degrees of freedom
##   (810 observations deleted due to missingness)
## AIC: 9727.6
##
## Number of Fisher Scoring iterations: 5
##
##   (Intercept) group2COVID19
##     31.643837      1.137952
##                    2.5 %    97.5 %
## (Intercept)    29.782435 33.505117
## group2COVID19   1.056161  1.227647
```

```
plot(p3 + theme(legend.position = "bottom"))
```

© 2023 Nolley EP et al. *JAMA Network Open.*

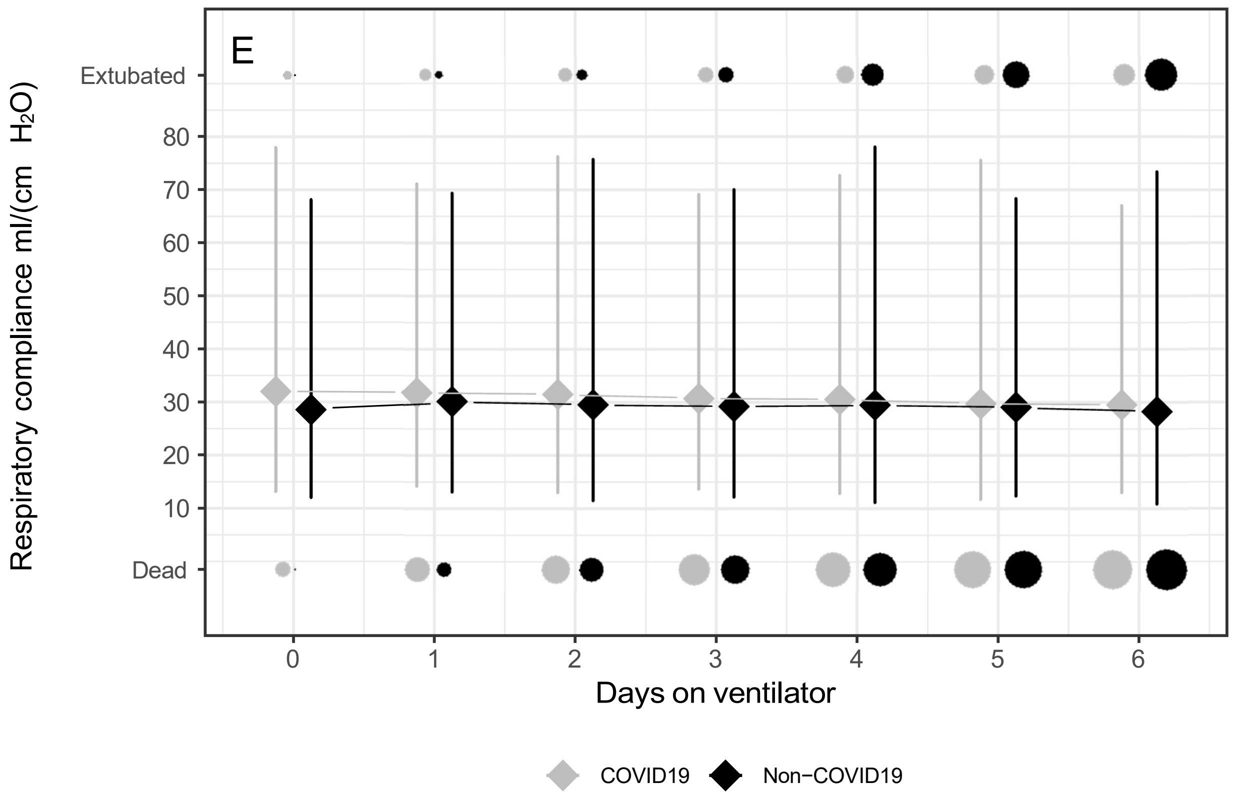


```
#Vent ratio
a = 0
b = 7
gap = (b-a)/20
p5 =
  fun.fig3("mvday", "group", "vent_ratio",
           "Ventilatory ratio",
           a, b, 2, "Days on ventilator",
           a-gap*4,
           a-gap*2.6,
           b+gap*2.6,
           b+gap*4,
           coord1 = a-gap*2 ,
           coord2 = b+gap*2 ,
           labl = 0.222, labh = 0.915, matched = 0, x1 = 0.12, x2 = 0.07,
           pprint = 1) +
  annotate("text", x = -Inf, y = Inf, hjust = -1, vjust = 2, size = 9, label = "F")
```

```
##
## Call:
## glm(formula = yvar ~ group2, family = gaussian(link = "log"),
##     data = dff[dff$mvday == 0, ])
##
## Deviance Residuals:
##     Min      1Q  Median      3Q     Max
## -1.446  -0.480  -0.189   0.229  34.460
```

© 2023 Nolley EP et al. *JAMA Network Open.*

```
##
## Coefficients:
##                  Estimate Std. Error t value Pr(>|t|)
## (Intercept)       0.38038    0.02945   12.91   <2e-16 ***
## group2COVID19 -0.10815   0.04872   -2.22   0.0266 *
## ---
## Signif. codes:  0 '***' 0.001 '**' 0.01 '*' 0.05 '.' 0.1 ' ' 1
##
## (Dispersion parameter for gaussian family taken to be 1.407161)
##
##      Null deviance: 1833.6  on 1299  degrees of freedom ##
Residual deviance: 1826.5  on 1298  degrees of freedom ##
(599 observations deleted due to missingness)
## AIC: 4137.3
##
## Number of Fisher Scoring iterations: 7
##
##   (Intercept) group2COVID19
##      1.462846       0.897492
##                    2.5 %  97.5 %
## (Intercept)      1.3783955 1.54729
## group2COVID19 0.8145924 0.98639
```

```
plot(p5 + theme(legend.position = "bottom"))
```

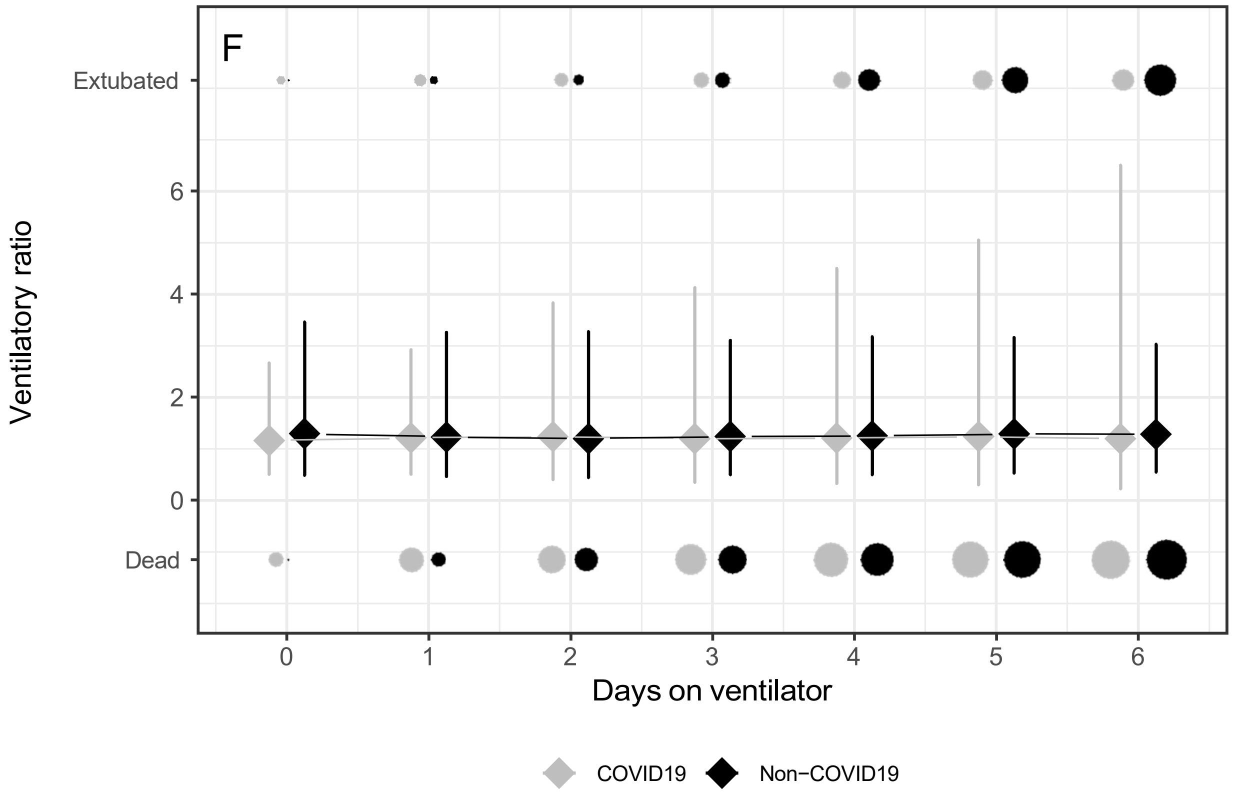

© 2023 Nolley EP et al. *JAMA Network Open.*

```r
### figure-4A

# VTPBW
a = 3
b = 12
gap = (b-a)/20
px =
  fun.fig3("mvday", "group", "vtpbw",
           "Tidal volume per predicted body weight (ml/kg)",
           a, b, 3, "Days on ventilator",
           b+gap*4,
           b+gap*2.6,
           a-gap*2.6,
           a-gap*4,
           coord1 = a-gap*2 ,
           coord2 = b+gap*2 ,
           labl = 0.225, labh = 0.91, matched = 0, x1 = 0.13, x2 = 0.08,
           pprint = 1) +
  annotate("text", x = -Inf, y = Inf, hjust = -1, vjust = 2, size = 9, label = "A")
```

```
##
##  Welch Two Sample t-test
##
## data:  yvar by group
## t = -4.1916, df = 1601.2, p-value = 2.922e-05
## alternative hypothesis: true difference in means is not equal to 0
## 95 percent confidence interval:
##  -0.3406830 -0.1234784
## sample estimates:
##     mean in group COVID19 mean in group Non-COVID19
##                  6.430798                  6.662879
```

```r
plot(px + theme(legend.position = "bottom"))
```

© 2023 Nolley EP et al. *JAMA Network Open.*

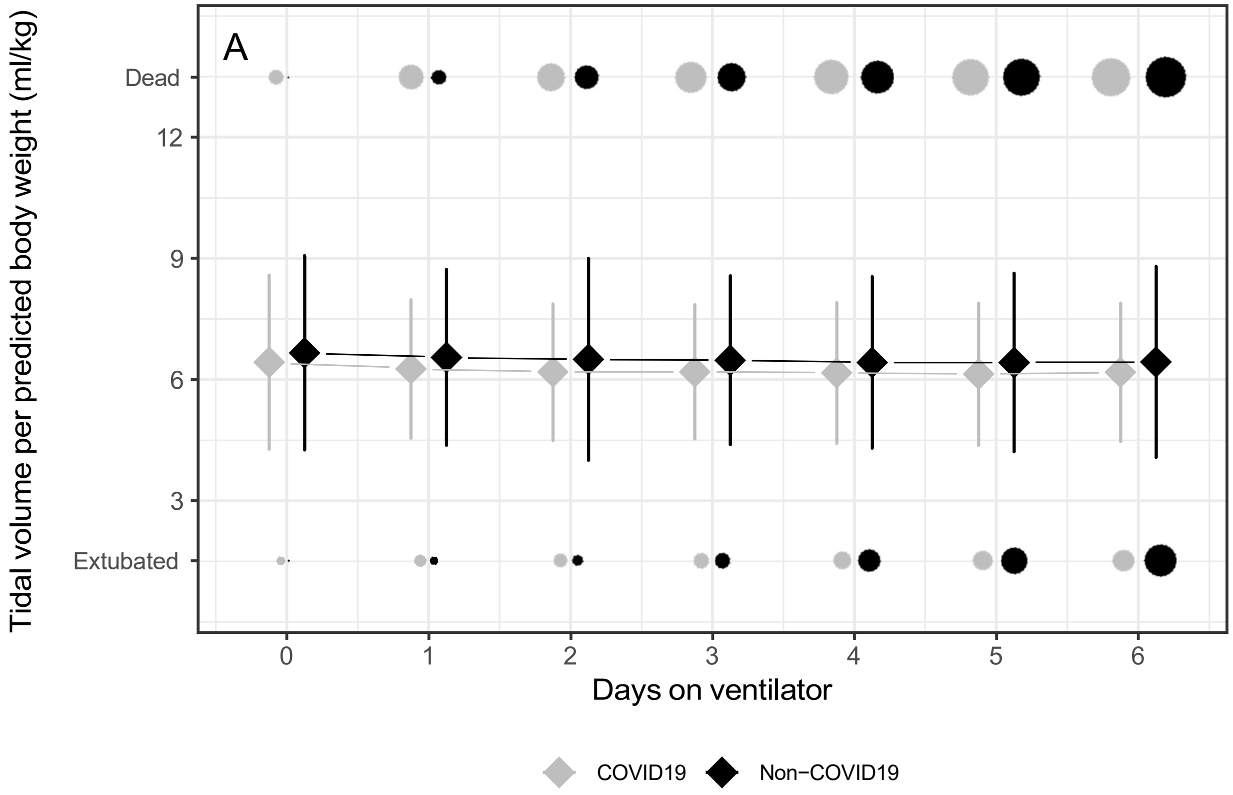


```
### figure-4B

# PEEP
a = 0
b = 21
gap = (b-a)/20
p9 =
  fun.fig3("mvday", "group", "peep",
           bquote(Positive~End~Expiratory~Pressure~"(cm "~H[2]*0*")"),
           a, b, 5, "Days on ventilator",
           b+gap*4,
           b+gap*2.6,
           a-gap*2.6,
           a-gap*4,
           coord1 = a-gap*2 ,
           coord2  = b+gap*2 ,
           labl  = 0.917, labh  = 0.217, matched  = 0, x1  = 0.125, x2  = 0.075,
           pprint  = 1) +
  annotate("text", x = -Inf, y = Inf, hjust = -1, vjust = 2, size = 9, label = "B")
```

```
##
##   Welch Two Sample t-test
##
## data:   yvar by group
## t = 21.429, df = 1283.8, p-value < 2.2e-16
## alternative hypothesis: true difference in means is not equal to 0
```

© 2023 Nolley EP et al. *JAMA Network Open.*

```
## 95 percent confidence interval:
##   3.135794  3.767813
## sample estimates:
##       mean in group COVID19 mean in group Non-COVID19
##                  10.055177                  6.603374
```

```
plot(p9 + theme(legend.position = "bottom"))
```

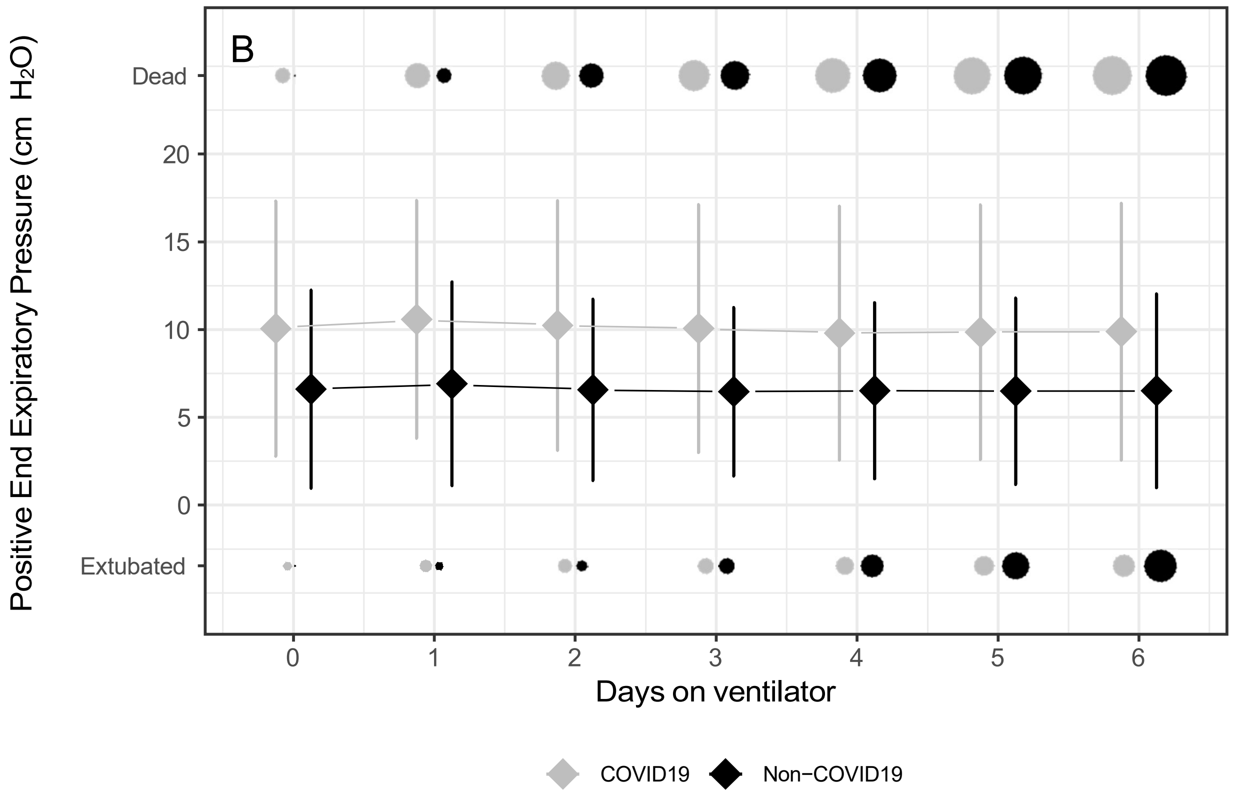


```
### figure-4C

#Pplat
a = 5
b = 40
gap = (b-a)/20
p.pplat =
  fun.fig3("mvday", "group", "pplat",
           bquote(Plateau~pressure~"(cm "~H[2]*0*")"),
           a, b, 5, "Days on ventilator",
           b+gap*4,
           b+gap*2.6,
           a-gap*2.6,
           a-gap*4,
           coord1 = a-gap*2 ,
           coord2 = b+gap*2 ,
           labl = 0.225, labh = 0.91, matched = 0, x1 = 0.13, x2 = 0.08,
```

© 2023 Nolley EP et al. *JAMA Network Open.*

```
        pprint = 1) +
  annotate("text", x = -Inf, y = Inf, hjust = -1, vjust = 2, size = 9, label = "C")
```

```
##
##  Welch Two Sample t-test
##
## data:  yvar by group
## t = 6.6598, df = 1326, p-value = 4.007e-11
## alternative hypothesis: true difference in means is not equal to 0
## 95 percent confidence interval:
##  1.407149 2.582316
## sample estimates:
##     mean in group COVID19 mean in group Non-COVID19
##                  23.70806                  21.71333
```

```
plot(p.pplat + theme(legend.position = "bottom"))
```

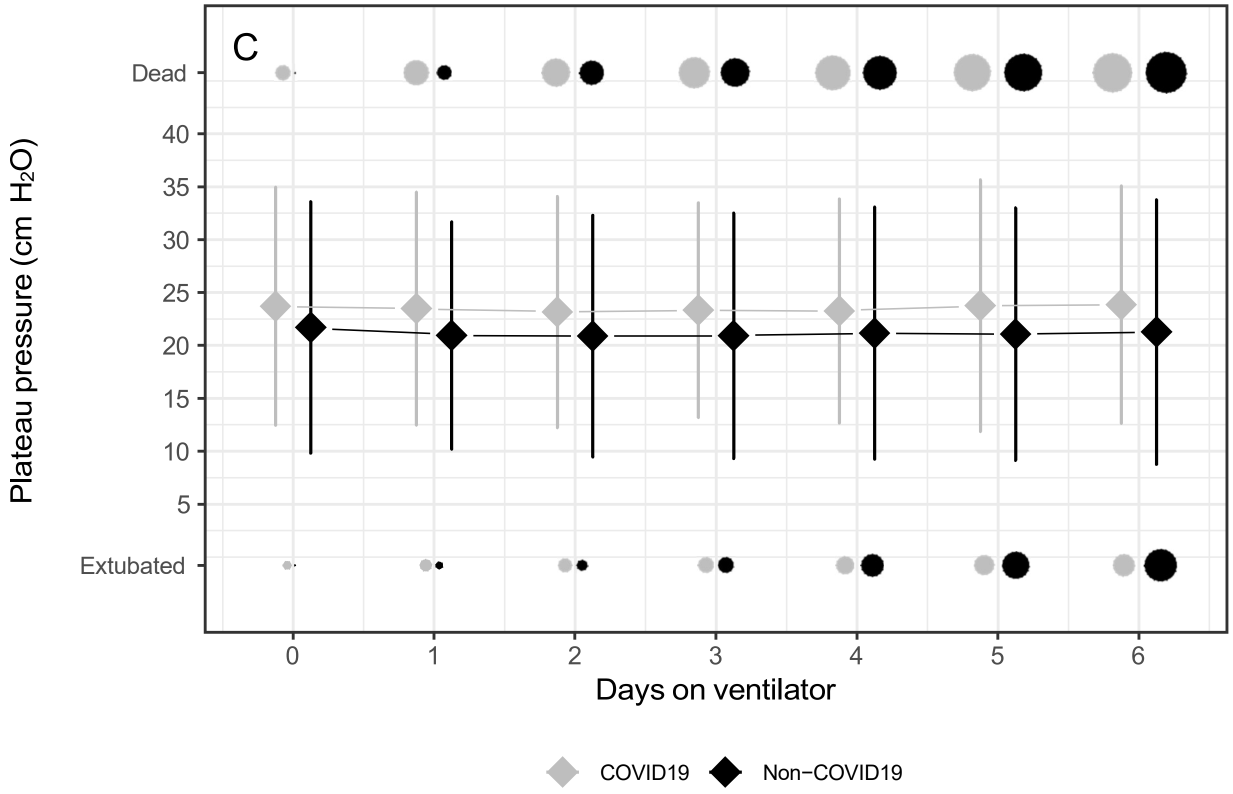


```
### figure-4D

# Driving pressure
a = 5
b = 32
gap = (b-a)/20
p11 =
  fun.fig3("mvday", "group", "pdriv",
```

© 2023 Nolley EP et al. *JAMA Network Open.*

```
          (Driving~pressure~"(cm "~H[2]*0*")"),
          a, b, 5, "Days on ventilator",
          b+gap*4,
          b+gap*2.6,
          a-gap*2.6,
          a-gap*4,
          coord1 = a-gap*2 ,
          coord2 = b+gap*2 ,
          labl = 0.217, labh = 0.917, matched = 0, x1 = 0.13, x2 = 0.08,
          pprint = 1) +
  annotate("text", x = -Inf, y = Inf, hjust = -1, vjust = 2, size = 9, label = "D")
```

```
##
##   Welch Two Sample t-test
##
## data:   yvar by group
## t = -5.4393, df = 1351.4, p-value = 6.342e-08
## alternative hypothesis: true difference in means is not equal to 0
## 95 percent confidence interval:
##  -1.9649903 -0.9233074
## sample estimates:
##     mean in group COVID19 mean in group Non-COVID19
##                  13.66584                  15.10999
```

```
plot(p11 + theme(legend.position = "bottom"))
```

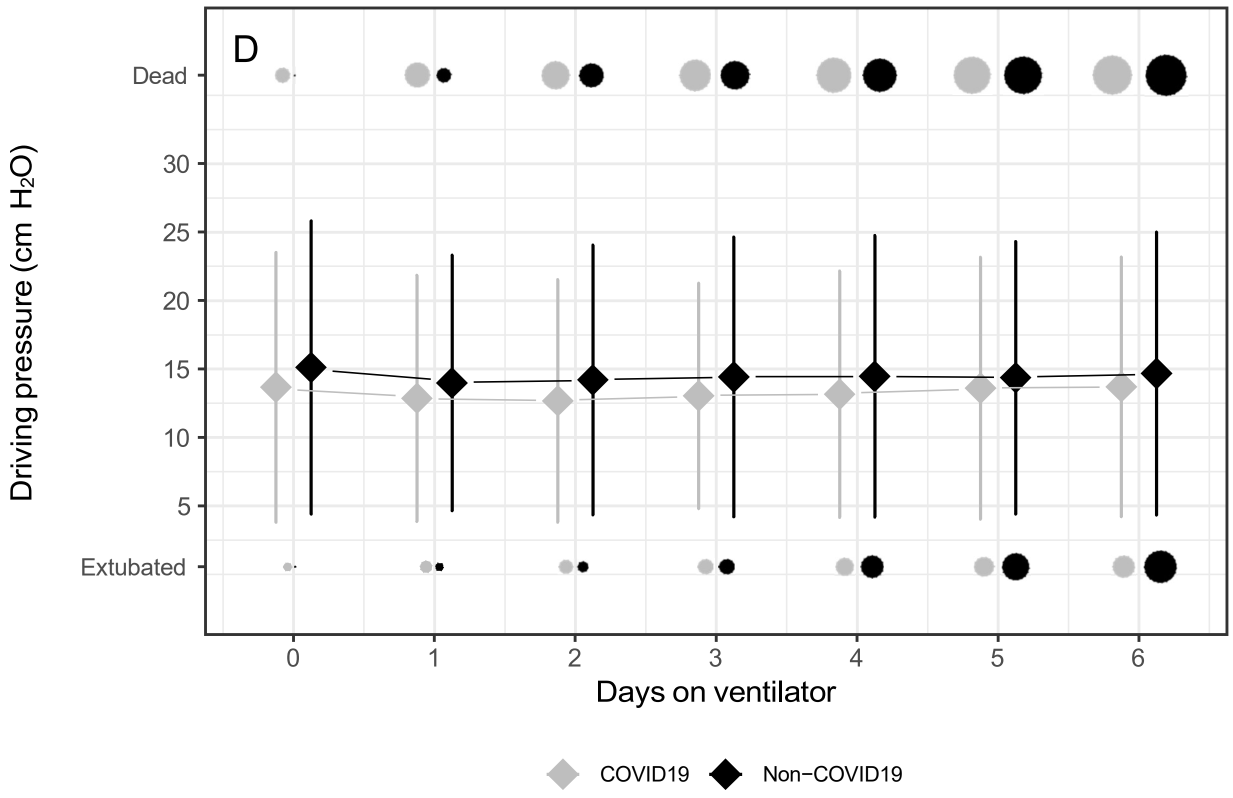

© 2023 Nolley EP et al. *JAMA Network Open.*

Figure-4: Respiratory Physiology over the first week of mechanical ventilation

```
p = grid.arrange(arrangeGrob(px, p9, p.pplat, p11, ncol = 2), legend, ncol = 1,
                 heights = unit.c(unit(0.95, "npc"), unit(0.05, "npc")))
```

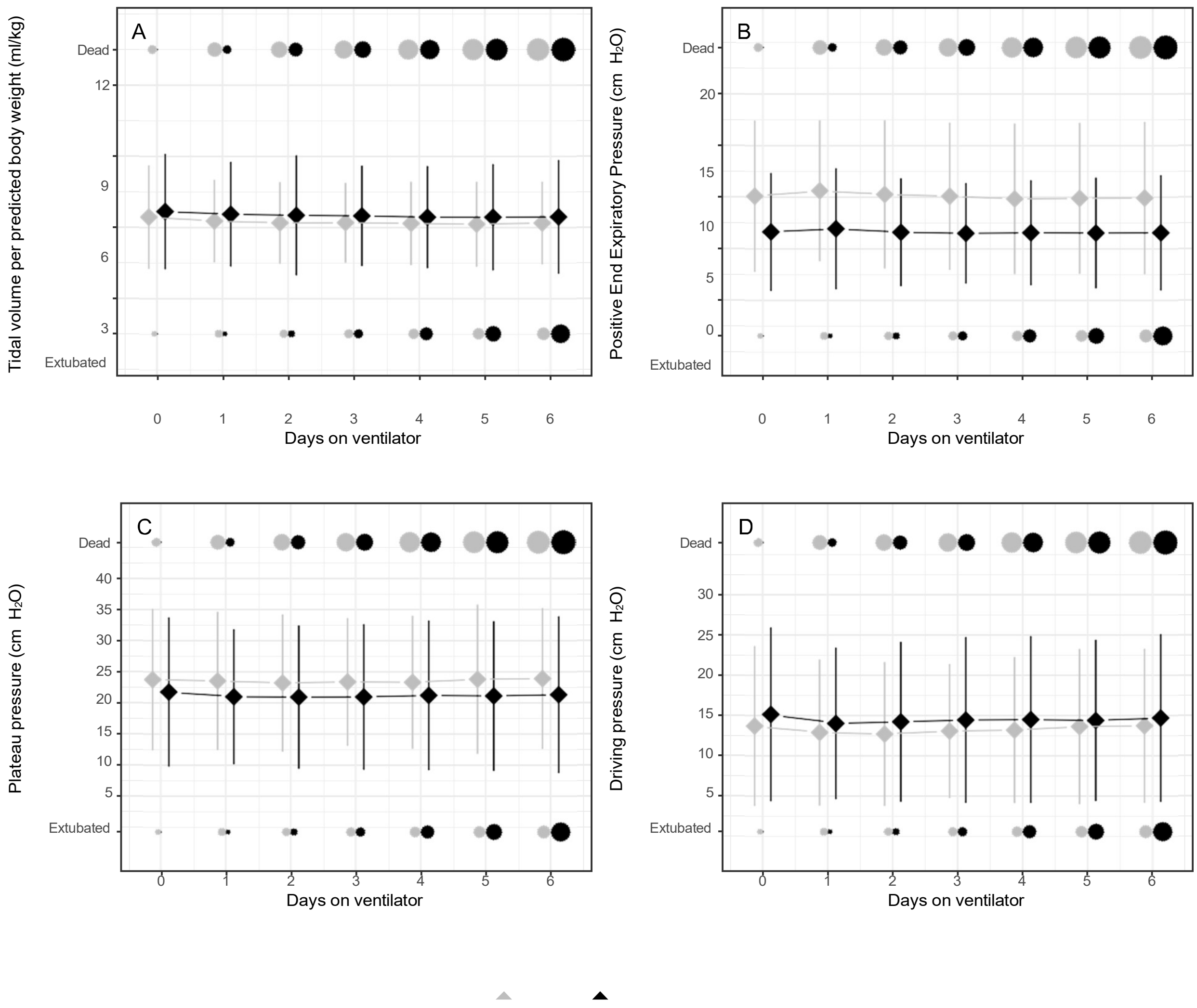

© 2023 Nolley EP et al. *JAMA Network Open.*

```
ggsave(p,
       filename = "Figure-4.jpg", dpi = 300, type = "cairo",
       width = 26, height = 20, units = "in")


ggsave(p3, filename = "Figure-5.png", dpi = 300, type = "cairo",
       width = 13, height = 10, units = "in")

ggsave(p5, filename = "Figure-6.png", dpi = 300, type = "cairo",
       width = 13, height = 10, units = "in")


ggsave(p,
       filename = "Figure-4.tiff", dpi = 300, type = "cairo",
       width = 26, height = 20, units = "in")
```

© 2023 Nolley EP et al. *JAMA Network Open.*

```
ggsave(p3, filename = "Figure-5.tiff", dpi = 300, type = "cairo",
       width = 13, height = 10, units = "in")

ggsave(p5, filename = "Figure-6.tiff", dpi = 300, type = "cairo",
       width = 13, height = 10, units = "in")

fig3_new = grid.arrange(arrangeGrob(px, p9, p.pplat, p11, p3, p5, ncol = 2), legend, ncol
↪  = 1,
                  heights = unit.c(unit(0.95, "npc"), unit(0.05, "npc")))
```

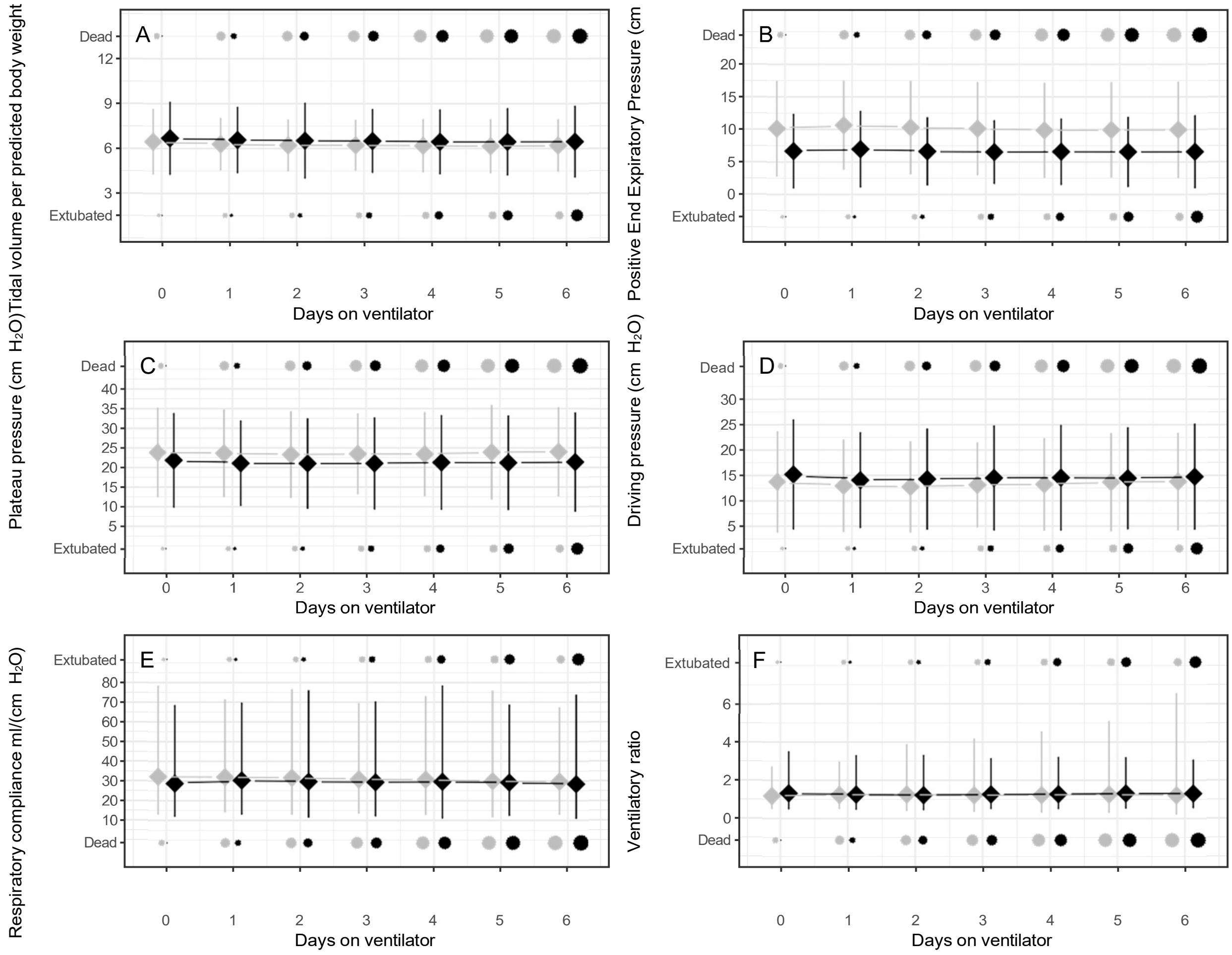

© 2023 Nolley EP et al. *JAMA Network Open.*

```
ggsave(fig3_new, filename = "Figure-3_new.pdf", device = "pdf",
       width = 24, height = 24, units = "in")


fun.notchbox = function(var = "vt",
                        y.var = "Tida volume",
                        ymin = min(dff$vt, na.rm = TRUE),
                        ymax = 800,
                        brk = 200){

dfx = read.csv("dat.csv")
dfx$vt = ifelse(dfx$vt == 0, NA, dfx$vt)
dfx$pdriv = ifelse(dfx$pdriv <= 0, NA, dfx$pdriv)

dfx$vtpbw = ifelse(dfx$vtpbw <= 0 , NA, dfx$vtpbw)

dfx$year = substr(dfx$id, 38, 41)

p = ggplot(data = dfx[dfx$group == "Non-COVID19",]) +
  geom_boxplot(aes_string(x = "factor(year)",
                          y = var,
                          group = "factor(year)")) +
  scale_x_discrete("Calendar year") +
  scale_y_continuous(y.var,
                     limits = c(ymin, ymax),
                     breaks = seq(ymin, ymax, brk)) +
  theme_bw() +
  theme(axis.title = element_text(size = 18),
        axis.text = element_text(size = 14))
return(p)
}

pn1 = fun.notchbox(var = "vtpbw",
                   y.var = "Tidal volume per predicted body weight (ml/kg)",
                   ymin = 3,
                   ymax = 12,
                   brk = 3) +
  theme(axis.title.x = element_blank(),
        axis.text.x = element_blank(),
        axis.ticks.x = element_blank())

pn2 = fun.notchbox(var = "pplat",
                   y.var = bquote(Plateau~pressure~"(cm "~H[2]*0*")"),
                   ymin = 5,
                   ymax = 40,
                   brk = 5) +
  theme(axis.title.x = element_blank(),
        axis.text.x = element_blank(),
        axis.ticks.x = element_blank())

pn3 = fun.notchbox(var = "peep",
                   y.var = bquote(Positive~End~Expiratory~Pressure~"(cm "~H[2]*0*")"),
                   ymin = 0,
                   ymax = 21,
```

© 2023 Nolley EP et al. *JAMA Network Open*.

```
                     brk = 3)

gn1 = ggplotGrob(pn1)
gn2 = ggplotGrob(pn2)
gn3 = ggplotGrob(pn3)

#pn = grid.arrange(arrangeGrob(pn1, pn3, pn2, ncol = 1))

library(gtable)
g <- rbind(gn1, gn2, gn3, size = "last")
g$height <- unit.pmax(gn1$height, gn2$height, gn3$height)
grid.newpage()
grid.draw(g)
```

© 2023 Nolley EP et al. *JAMA Network Open.*

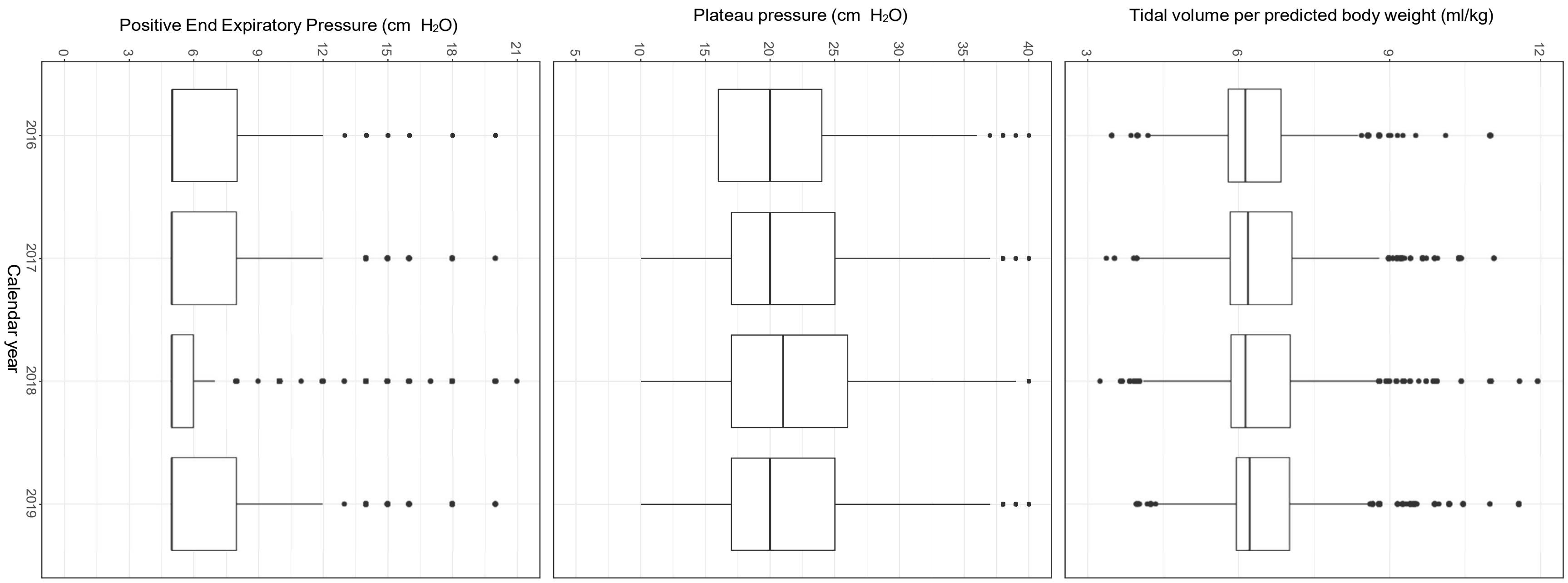

© 2023 Nolley EP et al. *JAMA Network Open.*

```
ggsave(g,
       filename = "Figure-E1.jpg", dpi = 300, type = "cairo",
       width = 12, height = 22, units = "in")
```

Secular trend in tidal volume by calendar year between 2016 and 2019

```
dss = read.csv("dat.csv")
dss <- dss %>% dplyr::filter(!(id %in% vap$id))

dss$year = substr(dss$id, 38, 41)

summary(lm(vtpbw ~ as.numeric(year), data = dss[dss$group == "Non-COVID19",]))
```

```
##
## Call:
## lm(formula = vtpbw ~ as.numeric(year), data = dss[dss$group ==
##     "Non-COVID19", ])
##
## Residuals:
##      Min       1Q  Median       3Q      Max
## -25.2653  -0.4118  -0.1337   0.7246  15.2318
##
## Coefficients:
##                   Estimate Std. Error t value Pr(>|t|)
## (Intercept)      -56.22887   50.31790  -1.117    0.264
## as.numeric(year)   0.03098    0.02494   1.242    0.214
##
## Residual standard error: 2.362 on 8508 degrees of freedom
##   (10473 observations deleted due to missingness)
## Multiple R-squared:  0.0001814,  Adjusted R-squared: 6.387e-05
## F-statistic: 1.543 on 1 and 8508 DF,  p-value: 0.2141
```

```
coef(lm(vtpbw ~ as.numeric(year), data = dss[dss$group == "Non-COVID19",]))
```

```
##      (Intercept) as.numeric(year)
##     -56.22886622       0.03097983
```

```
confint(lm(vtpbw ~ as.numeric(year), data = dss[dss$group == "Non-COVID19",]))
```

```
##                         2.5 %      97.5 %
## (Intercept)      -154.8641690 42.40643660
## as.numeric(year)   -0.0179011  0.07986077
```

Secular trend in PEEP by calendar year between 2016 and 2019

© 2023 Nolley EP et al. *JAMA Network Open.*

```r
summary(lm(peep ~ as.numeric(year), data = dss[dss$group == "Non-COVID19",]))
```

```
##
## Call:
## lm(formula = peep ~ as.numeric(year), data = dss[dss$group ==
##     "Non-COVID19", ])
##
## Residuals:
##    Min     1Q Median     3Q    Max ##
-1.405  -1.393  -1.382   1.595  15.618 ##
## Coefficients:
##                  Estimate Std. Error t value Pr(>|t|)
## (Intercept)      -17.07451   51.05417  -0.334    0.738
## as.numeric(year)   0.01163    0.02530   0.460    0.646
##
## Residual standard error: 2.565 on 9695 degrees of freedom
##   (9286 observations deleted due to missingness)
## Multiple R-squared:  2.179e-05,   Adjusted R-squared:  -8.135e-05
## F-statistic: 0.2113 on 1 and 9695 DF,   p-value: 0.6458
```

```r
coef(lm(peep ~ as.numeric(year), data = dss[dss$group == "Non-COVID19",]))
```

```
##      (Intercept) as.numeric(year)
##     -17.07451483       0.01162936
```

```r
confint(lm(peep ~ as.numeric(year), data = dss[dss$group == "Non-COVID19",]))
```

```
##                          2.5 %       97.5 %
## (Intercept)      -117.15134448 83.00231481
## as.numeric(year)   -0.03796632  0.06122503
```

Secular trend in driving pressure by calendar year between 2016 and 2019

```r
summary(lm(pdriv ~ as.numeric(year), data = dss[dss$group == "Non-COVID19",]))
```

```
##
## Call:
## lm(formula = pdriv ~ as.numeric(year), data = dss[dss$group ==
##     "Non-COVID19", ])
##
## Residuals:
##      Min       1Q   Median       3Q      Max
## -18.0318  -4.0318  -0.8905   2.9682  29.1095
##
## Coefficients:
##                  Estimate Std. Error t value Pr(>|t|)
```

© 2023 Nolley EP et al. *JAMA Network Open.*

```
## (Intercept)         -270.0756    121.4730  -2.223   0.0262 *
## as.numeric(year)       0.1413      0.0602   2.347   0.0190 *
## ---
## Signif. codes:  0 '***' 0.001 '**' 0.01 '*' 0.05 '.' 0.1 ' ' 1
##
## Residual standard error: 5.559 on 8019 degrees of freedom
##   (10962 observations deleted due to missingness)
## Multiple R-squared: 0.0006864,  Adjusted R-squared: 0.0005618
## F-statistic: 5.508 on 1 and 8019 DF,  p-value: 0.01895
```

```
coef(lm(pdriv ~ as.numeric(year), data = dss[dss$group == "Non-COVID19",]))
```

```
##      (Intercept) as.numeric(year)
##     -270.0755869        0.1412821
```

```
confint(lm(pdriv ~ as.numeric(year), data = dss[dss$group == "Non-COVID19",]))
```

```
##                          2.5 %      97.5 %
## (Intercept)      -508.19419062 -31.9569832
## as.numeric(year)    0.02327644   0.2592878
```

Secular trend in plateau pressure by calendar year between 2016 and 2019

```
summary(lm(pplat ~ as.numeric(year), data = dss[dss$group == "Non-COVID19",]))
```

```
##
## Call:
## lm(formula = pplat ~ as.numeric(year), data = dss[dss$group ==
##     "Non-COVID19", ])
##
## Residuals:
##      Min       1Q  Median       3Q      Max
## -11.6836  -4.5563  -0.9926   3.5710  27.5710
##
## Coefficients:
##                    Estimate Std. Error t value Pr(>|t|)
## (Intercept)      -235.33041  134.27596  -1.753   0.0797 .
## as.numeric(year)    0.12730    0.06654   1.913   0.0558 .
## ---
## Signif. codes:  0 '***' 0.001 '**' 0.01 '*' 0.05 '.' 0.1 ' ' 1
##
## Residual standard error: 6.154 on 8060 degrees of freedom
##   (10921 observations deleted due to missingness)
## Multiple R-squared: 0.0004538,  Adjusted R-squared: 0.0003298
## F-statistic:  3.66 on 1 and 8060 DF, p-value: 0.05578
```

```
coef(lm(pplat ~ as.numeric(year), data = dss[dss$group == "Non-COVID19",]))
```

© 2023 Nolley EP et al. *JAMA Network Open.*

```
##          (Intercept)  as.numeric(year)
##        -235.3304134             0.1272977
```

```
confint(lm(pplat ~ as.numeric(year), data = dss[dss$group == "Non-COVID19",]))
```

```
##                                      2.5  %        97.5  %
## (Intercept)           -4.985460e+02  27.8851585
## as.numeric(year) -3.145331e-03    0.2577407
```

Comparison of compliance in propensity score-matched data

```
# xvar = "mvday"
# groupvar = "group"
# yvar = "c_rs"

fun.p <- function(yvar , xvar, groupvar){

    dff <- dat

    dff$vt = ifelse(dff$vt == 0, NA, dff$vt)
    dff$pdriv = ifelse(dff$pdriv <= 0, NA, dff$pdriv)
    dff$vtpbw = ifelse(dff$vtpbw <=0 , NA, dff$vtpbw)

    dff$xvar = dff[[xvar]]
    dff$yvar = dff[[yvar]]
    dff$groupvar = dff[[groupvar]]

    dff = dff %>%
      dplyr::filter(xvar <= 6) %>%
      dplyr::group_by(id) %>%
      dplyr::summarise(yvar = mean(yvar, na.rm = TRUE)) %>%
      dplyr::right_join(df.matched[, c("id", "weights", "subclass", "group")], by = "id")
 ↪  %>%
      dplyr::select(id, yvar, group, weights, subclass)

dff$group <- factor(dff$group, levels = c("Non-COVID19", "COVID19"))


 fit <- glm(yvar ~ factor(group), data = dff,
                    weights = weights)
 print(summary(fit))

 substr(format(tidy(coeftest(fit, vcov. = vcovCL,
                    cluster = ~subclass),
           conf.int=TRUE)$p.value, scientific = F),1,5)[2] -> p

 print(tidy(coeftest(fit, vcov. = vcovCL,
                    cluster = ~subclass),
           conf.int=TRUE))
 p <- round(as.numeric(p), 2)
 return(p)
```

© 2023 Nolley EP et al. *JAMA Network Open.*

```
}

p.c_rs <- fun.p(xvar = "mvday", groupvar = "group", yvar = "c_rs")
```

```
##
## Call:
## glm(formula = yvar ~ factor(group), data = dff, weights = weights)
##
## Deviance Residuals:
##      Min       1Q    Median       3Q      Max
## -39.623   -8.304    -2.443    4.198   178.931
##
## Coefficients:
##                          Estimate Std. Error t value Pr(>|t|)
## (Intercept)               34.2479     0.6069  56.430   <2e-16 ***
## factor(group)COVID19 1.8213        0.8502    2.142    0.0324 *
## ---
## Signif. codes:  0 '***' 0.001 '**' 0.01 '*' 0.05 '.' 0.1 ' ' 1
##
## (Dispersion parameter for gaussian family taken to be 234.0098)
##
##     Null deviance: 278843  on 1188  degrees of freedom
## Residual deviance: 277770  on 1187  degrees of freedom
##   (597 observations deleted due to missingness)
## AIC: 10293
##
## Number of Fisher Scoring iterations: 2
##
## # A tibble: 2 x 7
##   term                    estimate std.error statistic    p.value conf.low  conf.high
##   <chr>                      <dbl>     <dbl>     <dbl>      <dbl>    <dbl>      <dbl>
## 1 (Intercept)                 34.2      1.30      26.3  4.21e-152     31.7       36.8
## 2 factor(group)COVID19        1.82      1.71      1.06  2.87e-    1    -1.53       5.17
```

Comparison of ventilatory ratio in propensity score-matched data

```
p.ventratio <- fun.p(xvar = "mvday", groupvar = "group", yvar = "vent_ratio")
```

```
##
## Call:
## glm(formula = yvar ~ factor(group), data = dff, weights = weights)
##
## Deviance  Residuals:
## Min              1Q Median           3Q       Max
## -4.5930    -0.3210    -0.1108     0.1516    15.1159
##
## Coefficients:
##                          Estimate Std. Error t value Pr(>|t|)
## (Intercept)               1.44568    0.02882  50.160   <2e-16 ***
## factor(group)COVID19 -0.05435      0.04434  -1.226        0.22
```

© 2023 Nolley EP et al. *JAMA Network Open.*

```
## ---
## Signif. codes:  0 '***' 0.001 '**' 0.01 '*' 0.05 '.' 0.1 ' ' 1
##
## (Dispersion parameter for gaussian family taken to be 0.6732519)
##
##     Null deviance: 996.08  on 1479  degrees of freedom ##
Residual deviance: 995.07  on 1478  degrees of freedom ##
(306 observations deleted due to missingness)
## AIC: 4440.3
##
## Number of Fisher Scoring iterations: 2
##
## # A tibble: 2 x 7
##   term                  estimate std.error statistic   p.value conf.low conf.high
##   <chr>                    <dbl>     <dbl>     <dbl>     <dbl>    <dbl>     <dbl>
## 1 (Intercept)               1.45    0.0745    19.4   8.32e-84    1.30      1.59
## 2 factor(group)COVID19 -0.0544    0.0838    -0.649  5.16e-  1   -0.219     0.110
```

Figure 3. Cumulative subdistribution curves for hospital mortality and discharge home alive at 90 days stratified by PaO /FiO ratio.

```
####################
min = -Inf
max = 150
pflab = "0-150"
maxday = 90
nodead = 1

fun.surv = function(dat, min, max, pflab, maxday, nodead, plotno){
  ### Subsetting the data based on day 0 pafi score
  df.surv = dat %>%
    dplyr::select(id, mvday, group, mvdate, mechvent, pafi) %>%
    dplyr::filter(mvday == 0) %>%
    dplyr::filter(pafi > min & pafi <= max)

  ### Merging the maximum day at hospital
  df.surv = left_join(df.surv, df.daily[,c("id", "maxday")], by = "id")

  ### If maximum day exceed the cut-off day, then replace the maximum day with cut-off
  ↪ day
  df.surv$maxday = replace(df.surv$maxday, df.surv$maxday >= maxday, maxday-1)

  ### Merging death/discharge data
  df.death_dis = df %>% dplyr::select(id, date_death, date_discharge)
  df.surv = left_join(df.surv, df.death_dis, by = "id") %>%
    dplyr::arrange(id, mvday)

  df.surv$died = replace(df.surv$died, as.Date(df.surv$date_death) -
↪   as.Date(df.surv$mvdate) <= maxday &
                                      as.Date(df.surv$date_death) ==
↪   as.Date(df.surv$date_discharge), 1)
```

© 2023 Nolley EP et al. *JAMA Network Open.*

```
df.surv$dis= replace(df.surv$dis, as.Date(df.surv$date_discharge) -
↪ as.Date(df.surv$mvdate) <= maxday &
                                  (as.Date(df.surv$date_death) != as.Date(df.surv$date_discharge)
↪ | is.na(df.surv$date_death)), 1)

df.surv$inh = replace(df.surv$inh, (is.na(df.surv$date_death) &
↪ is.na(df.surv$date_discharge) |
                                       as.Date(df.surv$date_discharge) -
↪ as.Date(df.surv$mvdate) > maxday), 1)

##############
#########################
##################################################
df.surv = df.surv %>%
  dplyr::filter(!(is.na(date_death) & is.na(date_discharge)))
##################################################
###########################
##############

df.surv$outcome = replace(df.surv$outcome, df.surv$died == 1, "Died")
df.surv$outcome = replace(df.surv$outcome, df.surv$dis == 1, "Discharged")
df.surv$outcome = replace(df.surv$outcome, df.surv$inh == 1, "Still in hospital")

tbl = df.surv %>%
  dplyr::group_by(group, maxday) %>%
  dplyr::summarise(n = n(),
            died = sum(died, na.rm = TRUE),
            dis = sum(dis, na.rm = TRUE),
            inh = sum(inh, na.rm = TRUE)) %>%
  dplyr::group_by(group)     %>%
  dplyr::mutate(cumdeath = cumsum(died)) %>%
  dplyr::mutate(cumdischarged = cumsum(dis)) %>%
  dplyr::mutate(died = cumsum(died)/sum(n)) %>% # For each day, cumsum/total Pt in
↪ this group
  dplyr::mutate(dis = cumsum(dis)/sum(n)) %>%
  dplyr::mutate(inh = cumsum(inh)/sum(n))

sumn_case = sum(tbl$n[tbl$group == "COVID19"], na.rm = TRUE)
sumn_ctrl = sum(tbl$n[tbl$group == "Non-COVID19"], na.rm = TRUE)

tbl$personatrisk = NA
tbl$personatrisk[tbl$group == "COVID19"] = sumn_case - (tbl$cumdeath[tbl$group ==
↪ "COVID19"] + tbl$cumdischarged[tbl$group == "COVID19"])
tbl$personatrisk[tbl$group == "Non-COVID19"] = sumn_ctrl - (tbl$cumdeath[tbl$group ==
↪ "Non-COVID19"] + tbl$cumdischarged[tbl$group == "Non-COVID19"])

tbl <- data.frame(tbl[,c("maxday","group","dis","died", "personatrisk", "cumdeath")])
tbl$died <- 1-tbl$died
tbl <- melt(tbl, id.vars = c("maxday","group", "personatrisk", "cumdeath"))


 df.dayn = data.frame(maxday = rep(0:(maxday-1),4),
                      group = c(rep("COVID19", 90), rep("Non-COVID19", 90),
                       ↪ rep("COVID19", 90), rep("Non-COVID19", 90)),
```

© 2023 Nolley EP et al. *JAMA Network Open.*

```
                       variable = c(rep("dis", 180), rep("died", 180)))


 tbl = left_join(df.dayn, tbl, by = c("maxday", "group", "variable"))
 tbl$value = ifelse(is.na(tbl$value) & tbl$maxday == 0 & tbl$variable == "dis", 0,
↪   tbl$value)
 tbl$value = ifelse(is.na(tbl$value) & tbl$maxday == 0 & tbl$variable == "died", 1,
↪   tbl$value)

 tbl= fill(tbl, personatrisk, cumdeath, value, group)

 if(nodead != 1){
   x.lab = NULL
   for(i in 0:89){
     xx = paste(i ,
                tbl$personatrisk[tbl$maxday == i & tbl$group == "COVID19"],
                tbl$personatrisk[tbl$maxday == i & tbl$group == "Non-COVID19"],
                tbl$cumdeath[tbl$maxday == i & tbl$group == "COVID19"],
                tbl$cumdeath[tbl$maxday == i & tbl$group == "Non-COVID19"], sep = "\n")
     x.lab = rbind(x.lab, c(i, xx))
   }
 }


 if(nodead == 1 ){
   x.lab = NULL
   for(i in 0:89){
     xx = paste(i ,
                tbl$personatrisk[tbl$maxday == i & tbl$group == "COVID19"],
                tbl$personatrisk[tbl$maxday == i & tbl$group == "Non-COVID19"], sep =
↪   "\n")
     x.lab = rbind(x.lab, c(i, xx))
   }
 }

 x.lab[,2][!(as.numeric(x.lab[,1]) %in% c(seq(0, 89, 5), 89))] <- ""

 p <-
     ggplot(data=tbl, aes(x=maxday, y=value*100, color=variable, linetype = group))+
     geom_step(size=1.5)+
 labs(x="Days", y="Proportion of participants (%)", color="")+
     scale_y_continuous(breaks = c(0, 25, 50, 75, 100),
                        expand=c(.01,.01),
                        sec.axis = sec_axis(~ .,breaks = c(0, 25, 50, 75, 100),
                                   labels=rev(c("0","25","50","75","100"))))   +

     scale_x_continuous(breaks=-0:(maxday-1), limits = c(0,(maxday-1)),
                        labels = x.lab[,2],
                        expand=c(.01,.01))+

     scale_color_manual(values=c("red","blue"),
                        labels=c("Died", "Discharged alive"))+
     annotate("text", x = 50, y = 5, label = pflab, size = 8) +
```

© 2023 Nolley EP et al. *JAMA Network Open.*

```
      annotate("text", x = 85, y = min(as.numeric(tbl$value[tbl$group == "Non-COVID19" &
↪  tbl$variable == "died"]))*100+ 5,
                  label = paste0(sprintf("%3.0f",(1-min(as.numeric(tbl$value[tbl$group ==
                   ↪  "Non-COVID19" & tbl$variable == "died"])))*100),"%"),
                  color = "red", size = 6) +

      annotate("text", x = 75, y = max(as.numeric(tbl$value[tbl$group == "Non-COVID19" &
↪  tbl$variable == "dis"]))*100+ 5,
                  label = paste0(sprintf("%3.0f",(max(as.numeric(tbl$value[tbl$group ==
                   ↪  "Non-COVID19" & tbl$variable == "dis"])))*100),"%"),
                  color = "blue", size = 6) +

      annotate("text", x = 85, y = min(as.numeric(tbl$value[tbl$group == "COVID19" &
↪  tbl$variable == "died"]))*100 - 5,
                  label = paste0(sprintf("%3.0f",(1-min(as.numeric(tbl$value[tbl$group ==
                   ↪  "COVID19" & tbl$variable == "died"])))*100),"%"),
                  color = "red", size = 6) +

      annotate("text", x = 75, y = max(as.numeric(tbl$value[tbl$group == "COVID19" &
↪  tbl$variable == "dis"]))*100 - 5,
                  label = paste0(sprintf("%3.0f",(max(as.numeric(tbl$value[tbl$group ==
                   ↪  "COVID19" & tbl$variable == "dis"])))*100),"%"),
                  color = "blue", size = 6) +
      theme_bw()+
      theme(text = element_text(size=18),
            plot.margin = unit(c(0, 0, 0, 1), "cm"),
            axis.title = element_text(size=20),
            panel.grid.major.x = element_blank(),
            panel.grid.minor = element_blank(),
            legend.position = "bottom",
            legend.title = element_blank(),
            panel.spacing = unit(1, "lines"),
            legend.background = element_blank(),
            strip.background = element_rect(fill="white"),
            strip.text = element_text(face="bold"),
            axis.text.y.left = element_text(color = "blue"),
            axis.text.y.right = element_text(color = "red"))

  return(p)
}


p1 = fun.surv(dat, 0, 150, "0-150", 90, nodead = 1) + theme(legend.position = "none")
p2 = fun.surv(dat, 151, Inf, ">150 +", maxday = 90, nodead = 1) + theme(legend.position =
↪  "none")

pp = fun.surv(dat, 0, 99, "", maxday = 90, nodead = 1)

g <- ggplotGrob(pp + theme(legend.position="bottom", rect = element_rect(fill =
↪  "transparent"),
                              legend.text = element_text(size=20)))$grobs
legend <- g[[which(sapply(g, function(x) x$name) == "guide-box")]]
lheight <- sum(legend$height)
```

© 2023 Nolley EP et al. *JAMA Network Open.*

```
legend$heights[2] = unit(c(0.25), "cm")


t0 <- ttheme_minimal(base_size = 13,
                     #base_colour = "", base_family = "",
                     rowhead=list(
                       fg_params=list(x=0, hjust = 0, fontface="bold"),
                       bg_params = list(
                         fill=c(NA,NA,NA,NA,NA),
                         col=c(NA,NA,NA,NA,NA),
                         alpha = rep(1,5))
                     )
)

t1 = ""
t2 = "      At risk (COVID19)"
t3 = "At risk (Non-COVID19)"
t4 = "        Dead (COVID19)"
t5 = "Dead (Non-COVID19)"

c1 = tableGrob(t1,theme=t0)
c2 = tableGrob(t2,theme=t0)
c3 = tableGrob(t3,theme=t0)
c4 = tableGrob(t4,theme=t0)
c5 = tableGrob(t5,theme=t0)

p1 = ggdraw() +
  draw_plot(p1, x = 0.1, y = 0.06, width = 0.9, height = 0.9) + # height 0.8 and y = 0.1
↪  if add dead
  draw_plot(c2, x = 0, y = 0.118, width = 1, height = 0.05, hjust = 0.395)+
  draw_plot(c3, x = 0, y = 0.088, width = 1, height = 0.05, hjust = 0.405)

p2 = ggdraw() +
  draw_plot(p2, x = 0.1, y = 0.06, width = 0.9, height = 0.9) +
  draw_plot(c2, x = 0, y = 0.118, width = 1, height = 0.05, hjust = 0.395)+
  draw_plot(c3, x = 0, y = 0.088, width = 1, height = 0.05, hjust = 0.405)


g1 = ggplotGrob(p1)
g2 = ggplotGrob(p2)
#g3 = ggplotGrob(legend)

ggdraw()+
  draw_grob(g1, x = 0, y = 0, height = 1, width = 0.5) +
  draw_grob(g2, x = 0.5, y = 0, height = 1, width = 0.5) +
  draw_grob(legend, x = 0, y = 0, height = 0.05, width = 1) -> p
p
```

© 2023 Nolley EP et al. *JAMA Network Open.*

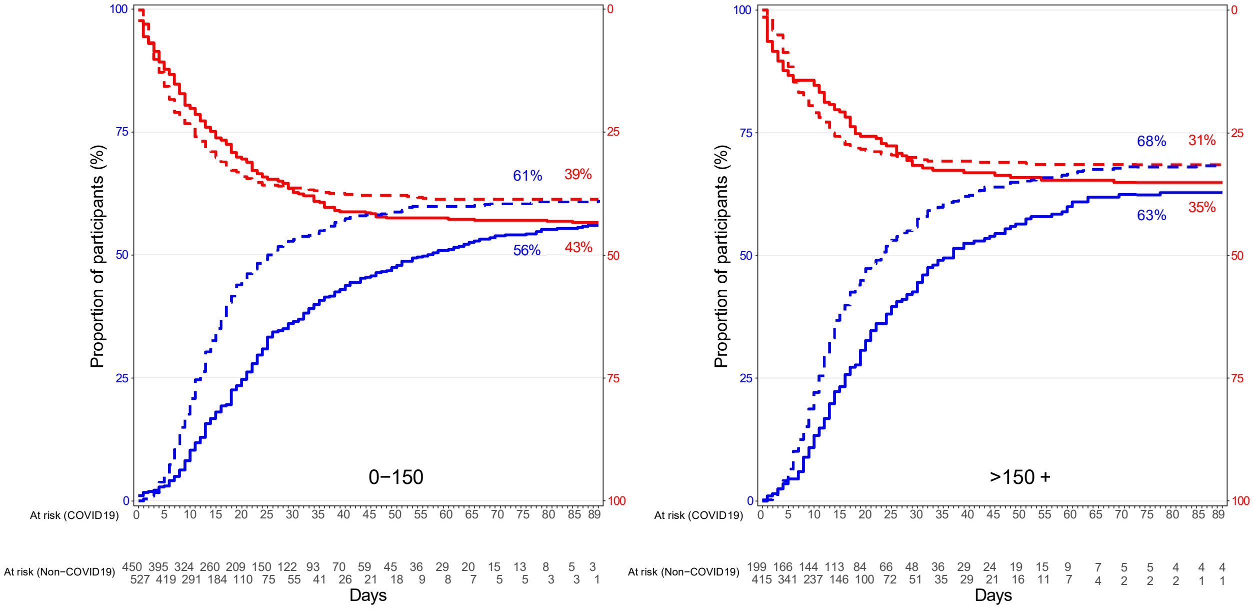

© 2023 Nolley EP et al. *JAMA Network Open.*

- COVID19 - Non−COVID19 - Died - Discharged alive

```
ggsave(p, filename = "Figure-2.jpg", dpi = 300, type = "cairo",
       width = 22, height = 8, units = "in")

ggsave(p, filename = "Figure-2.tiff", dpi = 300, type = "cairo",
       width = 22, height = 8, units = "in")

ggsave(p, filename = "Figure-2.pdf", device = "pdf",
       width = 22, height = 8, units = "in")


df.surv = dat %>%
  left_join(df[,c("id", "bmi" ,"race2", "sofa_calc", "charlson_mv", "ethnicity2")], by =
↪  "id") %>%
  dplyr::select(id, mvday, mvdate, mechvent) %>%
  dplyr::filter(mvday == 0)

### Merging death/discharge data
df.death_dis = df %>% dplyr::select(id, date_death, date_discharge)
df.surv = left_join(df.surv, df.death_dis, by = "id") %>%
  dplyr::arrange(id,  mvday)

### Last ventilator day
lastmvdate = dd %>%
  dplyr::filter(!(is.na(mvdate))) %>%
  dplyr::filter(mechvent == 1) %>%
  arrange(desc(mvdate)) %>%
  arrange(id) %>%
  dplyr::select(id, mvdate) %>%
  distinct(id, .keep_all = TRUE) %>%
  dplyr::rename(lastmvdate = mvdate)

df.surv = left_join(df.surv, lastmvdate, by = "id")

#### Transferred pt
# df.surv = df.surv %>%
#   filter(is.na(date_death))   %>%
#   filter(as.Date(date_discharge) == as.Date(lastmvdate))

df.surv$died <- df.surv$dis <- df.surv$invent <- NA

df.surv$deathdate = as.Date(df.surv$date_death) - as.Date(df.surv$mvdate)
df.surv$disdate = as.Date(df.surv$date_discharge) - as.Date(df.surv$mvdate)
#df.surv$disdate = ifelse(df.surv$deathdate == df.surv$disdate, NA, df.surv$disdate)
#df.surv$deathdate = ifelse(!is.na(df.surv$disdate) & (df.surv$disdate <
↪  df.surv$deathdate), NA, df.surv$deathdate)


### Death
# df.surv$died = replace(df.surv$died,
#                              as.Date(df.surv$date_death) - as.Date(df.surv$mvdate) <= 90 &
#                                as.Date(df.surv$date_death) ==
↪  as.Date(df.surv$date_discharge),   1)
```

© 2023 Nolley EP et al. *JAMA Network Open.*

```
df.surv$died = ifelse(df.surv$deathdate <= 90, 1, df.surv$died)
df.surv$died = ifelse(df.surv$disdate <= 90 & (df.surv$disdate != df.surv$deathdate), 0,
  df.surv$died)
df.surv$died = ifelse(df.surv$disdate <= 90 & is.na(df.surv$deathdate), 0, df.surv$died)
df.surv$died = ifelse(df.surv$disdate > 90, 0, df.surv$died)
df.surv$died = ifelse(is.na(df.surv$date_death) & is.na(df.surv$date_discharge), 0,
  df.surv$died)
table(df.surv$died, useNA = "always")
```

```
##
##    0    1 <NA>
## 1166  680    0
```

```
### Time to death
df.surv$time2death = ifelse(df.surv$died == 0, 90,
                            ifelse(df.surv$died == 1, as.Date(df.surv$date_death) -
                              as.Date(df.surv$mvdate), NA))


### Discharge
# df.surv$dis= replace(df.surv$dis,
#                      as.Date(df.surv$date_discharge) - as.Date(df.surv$mvdate) <= 90 &
#                        (as.Date(df.surv$date_death) != as.Date(df.surv$date_discharge)
  | is.na(df.surv$date_death)), 1)

df.surv$dis = ifelse(df.surv$disdate <= 90 & (df.surv$disdate != df.surv$deathdate), 1,
  df.surv$dis)
df.surv$dis = ifelse(df.surv$deathdate <= 90, 0, df.surv$dis)
df.surv$dis = ifelse(df.surv$died == 0 & df.surv$disdate <= 90, 1, df.surv$dis)
df.surv$dis = ifelse(df.surv$disdate > 90, 0, df.surv$dis)
df.surv$dis = ifelse(is.na(df.surv$date_death) & is.na(df.surv$date_discharge), 0,
  df.surv$dis)
```

© 2023 Nolley EP et al. *JAMA Network Open.*

```r
table(df.surv$dis, useNA = "always")
```

```
##
##     0    1 <NA>
##   694 1152    0
```

```r
### Time to discharge
df.surv$time2dis = ifelse(df.surv$dis == 0, 90,
                          ifelse(df.surv$dis == 1, as.Date(df.surv$date_discharge) -
                           as.Date(df.surv$mvdate), NA))


### In ventilator
# df.surv$invent = ifelse(df.surv$dis == 1 & (as.Date(df.surv$lastmvdate) <
  as.Date(df.surv$date_discharge)   |
#                                        as.Date(df.surv$lastmvdate) ==
  as.Date(df.surv$date_discharge)), 1,
#                         ifelse(df.surv$died == 1, 0,
#                                ifelse(df.surv$died == 0 & df.surv$dis == 0 , 1, NA)))

df.surv$invent = ifelse(df.surv$disdate <= 90 & (df.surv$disdate != df.surv$deathdate),
  1, df.surv$invent)
df.surv$invent = ifelse(df.surv$deathdate <= 90, 0, df.surv$invent)
df.surv$invent = ifelse(df.surv$died == 0 & df.surv$disdate <= 90, 1, df.surv$invent)
df.surv$invent = ifelse(df.surv$disdate > 90, 1, df.surv$invent)
df.surv$invent = ifelse(is.na(df.surv$date_death) & is.na(df.surv$date_discharge), 1,
  df.surv$invent)
df.surv$ihvent = ifelse(as.Date(df.surv$lastmvdate) == as.Date(df.surv$date_discharge),
  0, df.surv$invent)

table(df.surv$invent, useNA = "always")
```

```
##
##     0    1 <NA>
##   680 1166    0
```

```r
#df.surv = df.surv %>% dplyr::select(id,mvdate,lastmvdate, date_death, date_discharge,
  invent)


### Time on mechanical ventilation
df.surv$timeinvent = ifelse(df.surv$invent == 0, 90,
                            ifelse(df.surv$invent == 1, as.Date(df.surv$lastmvdate) -
                             as.Date(df.surv$mvdate), NA))

df.surv = df.surv %>%
  dplyr::select(id, invent, dis, died, time2death, time2dis, timeinvent)

dff = left_join(dff, df.surv, by = "id")

df.matched = left_join(df.matched, df.surv, by = "id")
```

© 2023 Nolley EP et al. *JAMA Network Open.*

```
### Converting IQR adjusted
dff$bmi_iqr = dff$bmi/IQR(dff$bmi, na.rm = TRUE)
dff$age_admit_iqr = dff$age_admit/IQR(dff$age_admit, na.rm = TRUE)
dff$pafi_mv0_iqr = dff$pafi_mv0/IQR(dff$pafi_mv0, na.rm = TRUE)

df.matched$bmi_iqr = df.matched$bmi/IQR(df.matched$bmi, na.rm = TRUE)
df.matched$age_admit_iqr = df.matched$age_admit/IQR(df.matched$age_admit, na.rm = TRUE)
df.matched$pafi_mv0_iqr = df.matched$pafi_mv0/IQR(df.matched$pafi_mv0, na.rm = TRUE)

### Function for Table 2-4

fun.x = function(datain1, datain2, ytime, yvar){
  y = paste0("Surv(", ytime,",", yvar, ")")
  tab = data.frame(matrix(ncol = 4, nrow = 9))

  # unadjusted
  tabx = NULL
  for(i in c("factor(group2)" ,"age_admit_iqr", "factor(ma0fe1)", "bmi_iqr",
   ↪ "factor(race2)", "factor(ethnicity2)",
              "sofa_calc", "charlson_mv", "pafi_mv0_iqr")){

    formula = as.formula(paste0(y, "~", i))
    xx = summary(coxph(formula, data = datain1))$conf.int
    message("Single variable")
    print(summary(coxph(formula, data = datain1)))
    # print(summary(coxph(formula, data = datain1)))

    aa = paste0(sprintf("%3.2f", xx[1]), " (", sprintf("%3.2f",xx[3]), "-",
 ↪  sprintf("%3.2f",xx[4]), ")")
    tabx = rbind(tabx, c(i, aa))
  }
  tab[, c(1,2)] = tabx

  # adjusted
  x = "factor(group2) + age_admit_iqr + factor(ma0fe1) + bmi_iqr + factor(race2) +
↪factor(ethnicity2) + sofa_calc + charlson_mv + pafi_mv0_iqr"
  formula2 = as.formula(paste0(y, "~", x))
  yy = summary(coxph(formula2, data = datain1))$conf.int

  message("Single variable")
  print(summary(coxph(formula2, data = datain1)))

  tab[, 3] = paste0(sprintf("%3.2f", yy[, 1]), " (", sprintf("%3.2f", yy[, 3]), "-",
 ↪  sprintf("%3.2f", yy[, 4]), ")")

  # Propensity matched
  formula3 = as.formula(paste0(y , "~ group2 +", var.adjusted))
  zz = summary(coxph(formula3, data = datain2, robust = TRUE, weights = weights, cluster
 ↪  = subclass))$conf.int[1,]
  tab[1,4] = paste0(sprintf("%3.2f", zz[1]), " (", sprintf("%3.2f",zz[3]), "-",
 ↪  sprintf("%3.2f",zz[4]), ")")
```

© 2023 Nolley EP et al. *JAMA Network Open.*

```
  message("Sampling weight adjusted propensity match with doubly robust estimation")
  print(summary(coxph(formula3, data = datain2, robust = TRUE, weights = weights, cluster
   ↪ = subclass)))

  tab[c(2:9), 4] = ""

  tab = data.frame(tab)
  colnames(tab) = c("", "Single variable", "Multiple variable", "After full matching")
   tab[[1]] = c("COVID-19", "Age in years (IQR range)", "Male sex", "Body mass index in
↪ Kg/m² (IQR range)", "Non-white range",
                    "Hispanic ethnicity", "SOFA score", "Charlson comorbidity index",
                     ↪ "PaO /FiO  (IQR range)")
  return(tab)
}


dff$ma0fe1 = ifelse(dff$ma0fe1 == 0, 2, dff$ma0fe1)
df.matched$ma0fe1 = ifelse(df.matched$ma0fe1 == 0, 2, df.matched$ma0fe1)
```

© 2023 Nolley EP et al. *JAMA Network Open.*

Single variable, multivariable and propensity-matched models for death at 90 days

```r
fun.x(dff, df.matched, ytime = "time2death", yvar = "died")
```

```
## Single variable

## Call:
## coxph(formula = formula, data = datain1)
##
##   n= 1846, number of events= 680
##
##                     coef exp(coef) se(coef)     z Pr(>|z|)
## factor(group2)1 0.18861   1.20757  0.07739 2.437   0.0148 *
## ---
## Signif. codes:  0 '***' 0.001 '**' 0.01 '*' 0.05 '.' 0.1 ' ' 1
##
##                 exp(coef) exp(-coef) lower .95 upper .95
## factor(group2)1     1.208     0.8281     1.038     1.405
##
## Concordance= 0.519  (se = 0.01 )
## Likelihood ratio test= 5.88  on 1 df,   p=0.02
## Wald test            = 5.94  on 1 df,   p=0.01
## Score (logrank) test = 5.96  on 1 df,   p=0.01

## Single variable

## Call:
## coxph(formula = formula, data = datain1)
##
##   n= 1846, number of events= 680
##
##                   coef exp(coef) se(coef)     z Pr(>|z|)
## age_admit_iqr 0.49318   1.63751  0.05385 9.158   <2e-16 ***
## ---
## Signif. codes:  0 '***' 0.001 '**' 0.01 '*' 0.05 '.' 0.1 ' ' 1
##
##               exp(coef) exp(-coef) lower .95 upper .95
## age_admit_iqr     1.638     0.6107     1.473      1.82
##
## Concordance= 0.6  (se = 0.011 )
## Likelihood ratio test= 89.62  on 1 df,   p=<2e-16
## Wald test            = 83.86  on 1 df,   p=<2e-16
## Score (logrank) test = 84.55  on 1 df,   p=<2e-16

## Single variable

## Call:
## coxph(formula = formula, data = datain1)
##
##   n= 1846, number of events= 680
```

© 2023 Nolley EP et al. *JAMA Network Open.*

```
##
##                        coef exp(coef) se(coef)     z Pr(>|z|)
## factor(ma0fe1)2 0.16114 1.17485 0.07801 2.066 0.0389 *
## ---
## Signif. codes: 0 '***' 0.001 '**' 0.01 '*' 0.05 '.' 0.1 ' ' 1
##
##                   exp(coef) exp(-coef) lower .95 upper .95
## factor(ma0fe1)2      1.175     0.8512     1.008     1.369
##
## Concordance= 0.518  (se = 0.01 )
## Likelihood ratio test= 4.3   on 1 df,   p=0.04
## Wald test            = 4.27  on 1 df,   p=0.04
## Score (logrank) test = 4.28  on 1 df,   p=0.04

## Single variable

## Call:
## coxph(formula = formula, data = datain1)
##
##   n= 1815, number of events= 656
##    (31 observations deleted due to missingness)
##
##              coef exp(coef) se(coef)      z Pr(>|z|)
## bmi_iqr -0.17401   0.84029  0.04764 -3.652  0.00026 ***
## ---
## Signif. codes: 0 '***' 0.001 '**' 0.01 '*' 0.05 '.' 0.1 ' ' 1
##
##         exp(coef) exp(-coef) lower .95 upper .95
## bmi_iqr 0.8403          1.19  0.7654  0.9225 ##
## Concordance= 0.544  (se = 0.011 )
## Likelihood ratio test= 14.96  on 1 df,  p=1e-04 ##
Wald test            = 13.34  on 1 df,   p=3e-04
## Score (logrank) test = 12.97  on 1 df,   p=3e-04

## Single variable

## Call:
## coxph(formula = formula, data = datain1)
##
##   n= 1827, number of events= 666
##    (19 observations deleted due to missingness)
##
##                     coef exp(coef) se(coef)      z Pr(>|z|)
## factor(race2)1 -0.08908   0.91477  0.07751 -1.149     0.25
##
##                  exp(coef) exp(-coef) lower .95 upper .95
## factor(race2)1     0.9148      1.093    0.7858     1.065
##
## Concordance= 0.515  (se = 0.01 )
## Likelihood ratio test= 1.32  on 1 df,   p=0.3
## Wald test            = 1.32  on 1 df,   p=0.3
## Score (logrank) test = 1.32  on 1 df,   p=0.3
```

© 2023 Nolley EP et al. *JAMA Network Open.*

```
## Single variable

## Call:
## coxph(formula = formula, data = datain1)
##
##   n= 1825, number of events= 665
##    (21 observations deleted due to missingness)
##
##                          coef exp(coef) se(coef)      z Pr(>|z|)
## factor(ethnicity2)1 -0.07506   0.92769  0.13854 -0.542    0.588
##
##                     exp(coef) exp(-coef) lower .95 upper .95
## factor(ethnicity2)1    0.9277      1.078    0.7071     1.217
##
## Concordance= 0.506  (se = 0.005 )
## Likelihood ratio test= 0.3  on 1 df,   p=0.6
## Wald test            = 0.29  on 1 df,   p=0.6
## Score (logrank) test = 0.29  on 1 df,   p=0.6

## Single variable

## Call:
## coxph(formula = formula, data = datain1)
##
##   n= 1846, number of events= 680
##
##                coef exp(coef)  se(coef)     z Pr(>|z|)
## sofa_calc -0.006821  0.993203  0.016228 -0.42    0.674
##
##           exp(coef) exp(-coef) lower .95 upper .95
## sofa_calc    0.9932      1.007    0.9621     1.025
##
## Concordance= 0.505  (se = 0.012 )
## Likelihood ratio test= 0.18  on 1 df,   p=0.7 ##
Wald test            = 0.18  on 1 df,   p=0.7
## Score (logrank) test = 0.18  on 1 df,   p=0.7

## Single variable

## Call:
## coxph(formula = formula, data = datain1)
##
##   n= 1846, number of events= 680
##
##                coef exp(coef) se(coef)     z Pr(>|z|)
## charlson_mv 0.07397   1.07678  0.01152 6.422 1.35e-10 ***
## ---
## Signif. codes:  0 '***' 0.001 '**' 0.01 '*' 0.05 '.' 0.1 ' ' 1
##
##             exp(coef) exp(-coef) lower .95 upper .95
## charlson_mv     1.077     0.9287     1.053     1.101
##
## Concordance= 0.571  (se = 0.011 )
```

© 2023 Nolley EP et al. *JAMA Network Open.*

```
## Likelihood ratio test= 37.86  on 1 df,   p=8e-10 ##
Wald test              = 41.24  on 1 df,   p=1e-10
## Score (logrank) test = 41.4  on 1 df,   p=1e-10

## Single variable

## Call:
## coxph(formula = formula, data = datain1)
##
##   n= 1846, number of events= 680
##
##                  coef exp(coef) se(coef)      z Pr(>|z|)
## pafi_mv0_iqr -0.38244   0.68219  0.05701 -6.708 1.97e-11 ***
## ---
## Signif. codes:  0 '***' 0.001 '**' 0.01 '*' 0.05 '.' 0.1 ' ' 1
##
##              exp(coef) exp(-coef) lower .95 upper .95
## pafi_mv0_iqr    0.6822      1.466    0.6101    0.7628
##
## Concordance= 0.615  (se = 0.012 )
## Likelihood ratio test= 54.78  on 1 df,   p=1e-13
## Wald test            = 45  on 1 df,   p=2e-11
## Score (logrank) test = 43.16  on 1 df,   p=5e-11

## Single variable

## Call:
## coxph(formula = formula2, data = datain1)
##
##   n= 1786, number of events= 635
##    (60 observations deleted due to missingness)
##
##                            coef  exp(coef)   se(coef)      z Pr(>|z|)
## factor(group2)1       0.1420571  1.1526424  0.0918633  1.546    0.122
## age_admit_iqr         0.4676112  1.5961767  0.0605093  7.728 1.09e-14 ***
## factor(ma0fe1)2       0.1243068  1.1323632  0.0836591  1.486    0.137
## bmi_iqr              -0.2107463  0.8099795  0.0539905 -3.903 9.49e-05 ***
## factor(race2)1       -0.0434355  0.9574944  0.0854448 -0.508    0.611
## factor(ethnicity2)1   0.0159672  1.0160954  0.1522113  0.105    0.916
## sofa_calc            -0.0004444  0.9995557  0.0178916 -0.025    0.980
## charlson_mv           0.0694604  1.0719296  0.0124285  5.589 2.29e-08 ***
## pafi_mv0_iqr         -0.4530157  0.6357082  0.0650847 -6.960 3.39e-12 ***
## ---
## Signif. codes:  0 '***' 0.001 '**' 0.01 '*' 0.05 '.' 0.1 ' ' 1
##
##                     exp(coef) exp(-coef) lower .95 upper .95
## factor(group2)1        1.1526     0.8676    0.9627    1.3800
## age_admit_iqr          1.5962     0.6265    1.4177    1.7972
## factor(ma0fe1)2        1.1324     0.8831    0.9611    1.3341
## bmi_iqr                0.8100     1.2346    0.7286    0.9004
## factor(race2)1         0.9575     1.0444    0.8099    1.1321
## factor(ethnicity2)1    1.0161     0.9842    0.7540    1.3693
## sofa_calc              0.9996     1.0004    0.9651    1.0352
```

© 2023 Nolley EP et al. *JAMA Network Open.*

charlson_mv 1.0719 0.9329 1.0461 1.0984
pafi_mv0_iqr 0.6357 1.5730 0.5596 0.7222
##
Concordance= 0.659 (se = 0.011)
Likelihood ratio test= 199.2 on 9 df, p=<2e-16
Wald test = 176.8 on 9 df, p=<2e-16
Score (logrank) test = 177 on 9 df, p=<2e-16

Sampling weight adjusted propensity match with doubly robust estimation

##	Call:						
##	coxph(formula = formula3, data = datain2,			weights = weights,			
##	robust = TRUE, cluster = subclass)						
##							
##	n= 1786, number of events= 635						
##							
##		coef	exp(coef)	se(coef)	robust se	z	Pr(>|z|)
##	group2	0.0409150	1.0417635	0.0789375	0.1312462	0.312	0.75524
##	age_admit	0.0389741	1.0397436	0.0030021	0.0060626	6.429	1.29e-10
##	bmi	-0.0230934	0.9771712	0.0040980	0.0075843	-3.045	0.00233
##	factor(ma0fe1)2	0.1217779	1.1295032	0.0867980	0.1155510	1.054	0.29193
##	charlson_mv	0.0260539	1.0263963	0.0144246	0.0228824	1.139	0.25487
##	pafi_mv0	-0.0046779	0.9953330	0.0005704	0.0011062	-4.229	2.35e-05
##	sofa_calc	0.0064647	1.0064856	0.0179582	0.0314201	0.206	0.83699
##	factor(race2)1	-0.0816944	0.9215536	0.0825271	0.1396981	-0.585	0.55869
##	factor(ethnicity2)1	0.1220450	1.1298049	0.1030722	0.2446951	0.499	0.61795

##				
##	group2			
##	age_admit ***			
##	bmi **			
##	factor(ma0fe1)2			
##	charlson_mv			
##	pafi_mv0 ***			
##	sofa_calc			
##	factor(race2)1			
##	factor(ethnicity2)1			
##	---			
##	Signif. codes: 0 '***' 0.001	'**' 0.01 '*' 0.05 '.'	0.1 ' '	1
##				

##		exp(coef)	exp(-coef)	lower .95	upper .95
##	group2	1.0418	0.9599	0.8055	1.3474
##	age_admit	1.0397	0.9618	1.0275	1.0522
##	bmi	0.9772	1.0234	0.9628	0.9918
##	factor(ma0fe1)2	1.1295	0.8853	0.9006	1.4166
##	charlson_mv	1.0264	0.9743	0.9814	1.0735
##	pafi_mv0	0.9953	1.0047	0.9932	0.9975
##	sofa_calc	1.0065	0.9936	0.9464	1.0704
##	factor(race2)1	0.9216	1.0851	0.7008	1.2118
##	factor(ethnicity2)1	1.1298	0.8851	0.6994	1.8251
##					

Concordance= 0.696 (se = 0.017)
Likelihood ratio test= 355.1 on 9 df, p=<2e-16
Wald test = 97.68 on 9 df, p=<2e-16
Score (logrank) test = 306.1 on 9 df, p=<2e-16, Robust = 65.71 p=1e-10

© 2023 Nolley EP et al. *JAMA Network Open.*

```
##
##    (Note: the likelihood ratio and score tests assume independence of
##       observations within a cluster, the Wald and robust score tests do not).
```

```
##                                              Single variable Multiple variable
## 1                                   COVID-19 1.21 (1.04-1.41)    1.15 (0.96-1.38)
## 2                  Age in years (IQR range) 1.64 (1.47-1.82)    1.60 (1.42-1.80)
## 3                                   Male sex 1.17 (1.01-1.37)    1.13 (0.96-1.33)
## 4 Body mass index in Kg/m² (IQR range) 0.84 (0.77-0.92)    0.81 (0.73-0.90)
## 5                          Non-white range 0.91 (0.79-1.06)    0.96 (0.81-1.13)
## 6                       Hispanic ethnicity 0.93 (0.71-1.22)    1.02 (0.75-1.37)
## 7                               SOFA score 0.99 (0.96-1.03)    1.00 (0.97-1.04)
## 8             Charlson comorbidity index 1.08 (1.05-1.10)    1.07 (1.05-1.10)
## 9                   PaO /FiO  (IQR range) 0.68 (0.61-0.76)    0.64 (0.56-0.72)
##   After full matching
## 1     1.04 (0.81-1.35)
## 2
## 3
## 4
## 5
## 6
## 7
## 8
## 9
```

```
death.p <- sprintf("%3.2f",tidy(coxph(Surv(time2death,died) ~group2,
                          data = df.matched ,
                          robust = TRUE,
                          weights = weights,
                          cluster = subclass))$p.value)

message("p-value for propensity-matched models for death at 90 days:")
```

```
## p-value for propensity-matched models for death at 90 days:
```

```
print(death.p)
```

```
## [1] "0.85"
```

© 2023 Nolley EP et al. *JAMA Network Open.*

Single variable, multivariable and propensity-matched models for time-on-mechanical-ventilation through 90 days

```r
fun.x(dff, df.matched, ytime = "time2dis", yvar = "dis")
```

```
## Single variable

## Call:
## coxph(formula = formula, data = datain1)
##
##   n= 1846, number of events= 1152
##
##                      coef exp(coef) se(coef)      z Pr(>|z|)
## factor(group2)1 -0.33434 0.71581  0.06145 -5.441  5.3e-08 ***
## ---
## Signif. codes:  0 '***' 0.001 '**' 0.01 '*' 0.05 '.' 0.1 ' ' 1
##
##                 exp(coef) exp(-coef) lower .95 upper .95
## factor(group2)1 0.7158         1.397   0.6346   0.8074  ##
## Concordance= 0.553  (se = 0.007 )
## Likelihood ratio test= 30.41   on 1 df,    p=4e-08
## Wald test            = 29.6   on 1 df,    p=5e-08
## Score (logrank) test = 29.88   on 1 df,    p=5e-08

## Single variable

## Call:
## coxph(formula = formula, data = datain1)
##
##   n= 1846, number of events= 1152
##
##                    coef exp(coef) se(coef)      z Pr(>|z|)
## age_admit_iqr -0.34021 0.71162  0.03692 -9.215   <2e-16 ***
## ---
## Signif. codes:  0 '***' 0.001 '**' 0.01 '*' 0.05 '.' 0.1 ' ' 1
##
##               exp(coef) exp(-coef) lower .95 upper .95
## age_admit_iqr    0.7116      1.405    0.6619     0.765
##
## Concordance= 0.574   (se = 0.009 )
## Likelihood ratio test= 82.05  on 1 df,   p=<2e-16 ##
Wald test                = 84.91  on 1 df,    p=<2e-16
## Score (logrank) test = 85.34  on 1 df,    p=<2e-16

## Single variable

## Call:
## coxph(formula = formula, data = datain1)
##
##   n= 1846, number of events= 1152
```

© 2023 Nolley EP et al. *JAMA Network Open.*

```
##
##                        coef  exp(coef)  se(coef)       z  Pr(>|z|)
## factor(ma0fe1)2  -0.15955 0.85253  0.05909  -2.7  0.00694 **
## ---
## Signif. codes:  0 '***' 0.001 '**' 0.01 '*' 0.05 '.' 0.1 ' ' 1
##
##                   exp(coef) exp(-coef) lower .95 upper .95
## factor(ma0fe1)2  0.8525          1.173    0.7593    0.9572  ##
## Concordance= 0.521   (se = 0.008 )
## Likelihood ratio test= 7.25  on 1 df,    p=0.007 ##
Wald test                   = 7.29  on 1 df,    p=0.007
## Score (logrank) test = 7.3   on 1 df,    p=0.007
```

```
## Single variable
```

```
## Call:
## coxph(formula = formula, data = datain1)
##
##   n= 1815, number of events= 1145
##    (31 observations deleted due to missingness)
##
##              coef  exp(coef)  se(coef)     z  Pr(>|z|)
## bmi_iqr 0.07373    1.07651   0.02634 2.8    0.00512 **
## ---
## Signif. codes:  0 '***' 0.001 '**' 0.01 '*' 0.05 '.' 0.1 ' ' 1
##
##          exp(coef) exp(-coef) lower .95 upper .95
## bmi_iqr      1.077      0.9289      1.022      1.134
##
## Concordance= 0.519   (se = 0.009 )
## Likelihood ratio test= 7.13   on 1 df,    p=0.008
## Wald test                = 7.84   on 1 df,    p=0.005
## Score (logrank) test = 7.78   on 1 df,    p=0.005
```

```
## Single variable
```

```
## Call:
## coxph(formula = formula, data = datain1)
##
##   n= 1827, number of events= 1148
##    (19 observations deleted due to missingness)
##
##                        coef  exp(coef)  se(coef)        z  Pr(>|z|)
## factor(race2)1  -0.01446    0.98564  0.05904 -0.245     0.807
##
##                   exp(coef) exp(-coef) lower .95 upper .95
## factor(race2)1       0.9856       1.015      0.8779      1.107
##
## Concordance= 0.505   (se = 0.008 )
## Likelihood ratio test= 0.06   on 1 df,    p=0.8
## Wald test                = 0.06   on 1 df,    p=0.8
## Score (logrank) test = 0.06   on 1 df,    p=0.8
```

© 2023 Nolley EP et al. *JAMA Network Open.*

```
## Single variable

## Call:
## coxph(formula = formula, data = datain1)
##
##   n= 1825, number of events= 1147
##    (21 observations deleted due to missingness)
##
##                          coef exp(coef) se(coef)     z Pr(>|z|)
## factor(ethnicity2)1 -0.1205    0.8864   0.1066 -1.13    0.258
##
##                     exp(coef) exp(-coef) lower .95 upper .95
## factor(ethnicity2)1    0.8864      1.128    0.7192     1.092
##
## Concordance= 0.508  (se = 0.004 )
## Likelihood ratio test= 1.32  on 1 df,   p=0.3 ##
Wald test            = 1.28  on 1 df,   p=0.3
## Score (logrank) test = 1.28  on 1 df,   p=0.3

## Single variable

## Call:
## coxph(formula = formula, data = datain1)
##
##   n= 1846, number of events= 1152
##
##               coef exp(coef) se(coef)     z Pr(>|z|)
## sofa_calc 0.003357  1.003362 0.011618 0.289    0.773
##
##           exp(coef) exp(-coef) lower .95 upper .95
## sofa_calc     1.003     0.9966    0.9808     1.026
##
## Concordance= 0.502  (se = 0.009 )
## Likelihood ratio test= 0.08  on 1 df,   p=0.8
## Wald test            = 0.08  on 1 df,   p=0.8
## Score (logrank) test = 0.08  on 1 df,   p=0.8

## Single variable

## Call:
## coxph(formula = formula, data = datain1)
##
##   n= 1846, number of events= 1152
##
##                  coef exp(coef) se(coef)      z Pr(>|z|)
## charlson_mv -0.06721   0.93500  0.01066 -6.303 2.92e-10 ***
## ---
## Signif. codes:  0 '***' 0.001 '**' 0.01 '*' 0.05 '.' 0.1 ' ' 1
##
##             exp(coef) exp(-coef) lower .95 upper .95
## charlson_mv 0.935          1.07   0.9157    0.9547 ##
## Concordance= 0.558  (se = 0.009 )
```

© 2023 Nolley EP et al. *JAMA Network Open.*

```
## Likelihood ratio test= 42.67  on 1 df,   p=6e-11 ##
Wald test                    = 39.73  on 1 df,    p=3e-10
## Score (logrank) test = 39.89  on 1 df,    p=3e-10

## Single variable

## Call:
## coxph(formula = formula, data = datain1)
##
##   n= 1846, number of events= 1152
##
##                  coef exp(coef) se(coef)     z Pr(>|z|)
## pafi_mv0_iqr 0.17929   1.19637  0.02447 7.326 2.38e-13 ***
## ---
## Signif. codes:  0 '***' 0.001 '**' 0.01 '*' 0.05 '.' 0.1 ' ' 1
##
##              exp(coef) exp(-coef) lower .95 upper .95
## pafi_mv0_iqr     1.196     0.8359      1.14     1.255
##
## Concordance= 0.598  (se = 0.009 )
## Likelihood ratio test= 44.75  on 1 df,    p=2e-11
## Wald test            = 53.66  on 1 df,    p=2e-13
## Score (logrank) test = 53.33  on 1 df,    p=3e-13

## Single variable

## Call:
## coxph(formula = formula2, data = datain1)
##
##   n= 1786, number of events= 1138
##    (60 observations deleted due to missingness)
##
##                           coef exp(coef)  se(coef)      z Pr(>|z|)
## factor(group2)1      -0.326840  0.721199  0.070812 -4.616 3.92e-06 ***
## age_admit_iqr        -0.281811  0.754417  0.038382 -7.342 2.10e-13 ***
## factor(ma0fe1)2      -0.095358  0.909047  0.060653 -1.572  0.11590
## bmi_iqr               0.082001  1.085456  0.026219  3.127  0.00176 **
## factor(race2)1        0.006997  1.007022  0.063002  0.111  0.91157
## factor(ethnicity2)1  -0.099108  0.905645  0.114880 -0.863  0.38830
## sofa_calc             0.011932  1.012003  0.012475  0.956  0.33885
## charlson_mv          -0.071273  0.931208  0.011124 -6.407 1.48e-10 ***
## pafi_mv0_iqr          0.188138  1.207000  0.027770  6.775 1.24e-11 ***
## ---
## Signif. codes:  0 '***' 0.001 '**' 0.01 '*' 0.05 '.' 0.1 ' ' 1
##
##                     exp(coef) exp(-coef) lower .95 upper .95
## factor(group2)1        0.7212     1.3866    0.6277    0.8286
## age_admit_iqr          0.7544     1.3255    0.6997    0.8134
## factor(ma0fe1)2        0.9090     1.1001    0.8072    1.0238
## bmi_iqr                1.0855     0.9213    1.0311    1.1427
## factor(race2)1         1.0070     0.9930    0.8900    1.1394
## factor(ethnicity2)1    0.9056     1.1042    0.7231    1.1343
## sofa_calc              1.0120     0.9881    0.9876    1.0371
```

© 2023 Nolley EP et al. *JAMA Network Open.*

```
## charlson_mv              0.9312     1.0739     0.9111     0.9517
## pafi_mv0_iqr             1.2070     0.8285     1.1431     1.2745
##
## Concordance= 0.634  (se = 0.008 )
## Likelihood ratio test= 204.6  on 9 df,   p=<2e-16 ##
Wald test                 = 212.7  on 9 df,   p=<2e-16
## Score (logrank) test = 215.3  on 9 df,   p=<2e-16

## Sampling weight adjusted propensity match with doubly robust estimation

## Call:
## coxph(formula = formula3, data = datain2,  weights = weights,
##     robust = TRUE, cluster = subclass)
##
##   n= 1786, number of events= 1138
##
##                            coef  exp(coef)   se(coef)  robust se      z  Pr(>|z|)
## group2               -0.2148746  0.8066426  0.0635367  0.1087636 -1.976   0.0482
## age_admit            -0.0252115  0.9751036  0.0019020  0.0041790 -6.033 1.61e-09
## bmi                   0.0052010  1.0052146  0.0017022  0.0023365  2.226   0.0260
## factor(ma0fe1)2      -0.1020897  0.9029486  0.0645737  0.0930041 -1.098   0.2723
## charlson_mv          -0.0180216  0.9821398  0.0122115  0.0215796 -0.835   0.4036
## pafi_mv0              0.0018224  1.0018240  0.0002567  0.0004102  4.442 8.90e-06
## sofa_calc            -0.0010515  0.9989491  0.0127925  0.0248152 -0.042   0.9662
## factor(race2)1        0.0636843  1.0657559  0.0659142  0.1182409  0.539   0.5902
## factor(ethnicity2)1  -0.2326609  0.7924222  0.0860665  0.2823247 -0.824   0.4099
##
## group2               *
## age_admit            ***
## bmi                  *
## factor(ma0fe1)2
## charlson_mv
## pafi_mv0             ***
## sofa_calc
## factor(race2)1
## factor(ethnicity2)1
## ---
## Signif. codes:  0 '***' 0.001 '**' 0.01 '*' 0.05 '.' 0.1 ' ' 1
##
##                      exp(coef) exp(-coef) lower .95 upper .95
## group2                  0.8066     1.2397    0.6518    0.9983
## age_admit               0.9751     1.0255    0.9671    0.9831
## bmi                     1.0052     0.9948    1.0006    1.0098
## factor(ma0fe1)2         0.9029     1.1075    0.7525    1.0835
## charlson_mv             0.9821     1.0182    0.9415    1.0246
## pafi_mv0                1.0018     0.9982    1.0010    1.0026
## sofa_calc               0.9989     1.0011    0.9515    1.0487
## factor(race2)1          1.0658     0.9383    0.8453    1.3437
## factor(ethnicity2)1     0.7924     1.2620    0.4557    1.3781
##
## Concordance= 0.651  (se = 0.023 )
## Likelihood ratio test= 272.4    on 9 df,   p=<2e-16
## Wald test                = 121.6   on 9 df,   p=<2e-16
## Score (logrank) test = 300.6   on 9 df,   p=<2e-16,   Robust = 76.84   p=7e-13
```

© 2023 Nolley EP et al. *JAMA Network Open.*

```
##
##   (Note: the likelihood ratio and score tests assume independence of
##        observations within a cluster, the Wald and robust score tests do not).

##                                               Single variable  Multiple variable
## 1                                   COVID-19 0.72 (0.63-0.81)   0.72 (0.63-0.83)
## 2                     Age in years (IQR range) 0.71 (0.66-0.77)   0.75 (0.70-0.81)
## 3                                   Male sex 0.85 (0.76-0.96)   0.91 (0.81-1.02)
## 4 Body mass index  in Kg/m^2 (IQR range) 1.08 (1.02-1.13)   1.09 (1.03-1.14)
## 5                           Non-white range 0.99 (0.88-1.11)   1.01 (0.89-1.14)
## 6                        Hispanic ethnicity 0.89 (0.72-1.09)   0.91 (0.72-1.13)
## 7                                 SOFA score 1.00 (0.98-1.03)   1.01 (0.99-1.04)
## 8                Charlson comorbidity index 0.93 (0.92-0.95)    0.93 (0.91-0.95)
## 9                     PaO /FiO  (IQR range) 1.20 (1.14-1.26)    1.21 (1.14-1.27)
##  After full matching
## 1     0.81 (0.65-1.00)
## 2
## 3
## 4
## 5
## 6
## 7
## 8
## 9
```

© 2023 Nolley EP et al. *JAMA Network Open.*

Single variable, multivariable, and propensity-matched models for time-to-discharge from hospital alive through 90 days

```
fun.x(dff, df.matched, ytime = "timeinvent", yvar = "invent")
```

```
## Single variable

## Call:
## coxph(formula = formula, data = datain1)
##
##   n= 1846, number of events= 1166
##
##                      coef exp(coef) se(coef)      z Pr(>|z|)
## factor(group2)1 -0.33834 0.71295  0.06098 -5.548 2.89e-08 ***
## ---
## Signif. codes:  0 '***' 0.001 '**' 0.01 '*' 0.05 '.' 0.1 ' ' 1
##
##                 exp(coef) exp(-coef) lower .95 upper .95
## factor(group2)1 0.713          1.403    0.6326    0.8035 ##
## Concordance= 0.557  (se = 0.007 )
## Likelihood ratio test= 31.6    on 1 df,    p=2e-08
## Wald test             = 30.78  on 1 df,    p=3e-08
## Score (logrank) test = 31.07   on 1 df,    p=2e-08

## Single variable

## Call:
## coxph(formula = formula, data = datain1)
##
##   n= 1846, number of events= 1166
##
##                    coef exp(coef) se(coef)      z Pr(>|z|)
## age_admit_iqr -0.3284     0.7201    0.0368 -8.924   <2e-16 ***
## ---
## Signif. codes:  0 '***' 0.001 '**' 0.01 '*' 0.05 '.' 0.1  ' ' 1
##
##               exp(coef) exp(-coef) lower .95 upper .95
## age_admit_iqr    0.7201      1.389      0.67    0.7739
##
## Concordance= 0.566   (se = 0.009 )
## Likelihood ratio test= 77.14   on 1 df,    p=<2e-16
## Wald test              = 79.64   on 1 df,    p=<2e-16
## Score (logrank) test = 80   on 1 df,    p=<2e-16

## Single variable

## Call:
## coxph(formula = formula, data = datain1)
##
##   n= 1846, number of events= 1166
```

© 2023 Nolley EP et al. *JAMA Network Open.*

```
##
##                          coef exp(coef) se(coef)      z Pr(>|z|)
## factor(ma0fe1)2 -0.13243 0.87596 0.05874 -2.254  0.0242 *
## ---
## Signif. codes: 0 '***' 0.001 '**' 0.01 '*' 0.05 '.' 0.1 ' ' 1
##
##                     exp(coef) exp(-coef) lower .95 upper .95
## factor(ma0fe1)2 0.876          1.142  0.7807  0.9829 ##
## Concordance= 0.516  (se = 0.008 )
## Likelihood ratio test= 5.06  on 1 df,   p=0.02 ##
Wald test                   = 5.08  on 1 df,   p=0.02
## Score (logrank) test = 5.09  on 1 df,   p=0.02

## Single variable

## Call:
## coxph(formula = formula, data = datain1)
##
##   n= 1815, number of events= 1159
##    (31 observations deleted due to missingness)
##
##             coef exp(coef) se(coef)     z Pr(>|z|)
## bmi_iqr 0.06173 1.06368 0.02655 2.326    0.02 *
## ---
## Signif. codes: 0 '***' 0.001 '**' 0.01 '*' 0.05 '.' 0.1 ' ' 1
##
##          exp(coef) exp(-coef) lower .95 upper .95
## bmi_iqr     1.064     0.9401      1.01      1.12
##
## Concordance= 0.513  (se = 0.009 )
## Likelihood ratio test= 5.02  on 1 df,   p=0.03 ##
Wald test                   = 5.41  on 1 df,   p=0.02
## Score (logrank) test = 5.38  on 1 df,   p=0.02

## Single variable

## Call:
## coxph(formula = formula, data = datain1)
##
##   n= 1827, number of events= 1161
##    (19 observations deleted due to missingness)
##
##                        coef exp(coef) se(coef)       z Pr(>|z|)
## factor(race2)1 -0.02368   0.97660 0.05872 -0.403    0.687
##
##                   exp(coef) exp(-coef) lower .95 upper .95
## factor(race2)1     0.9766      1.024    0.8704     1.096
##
## Concordance= 0.511  (se = 0.008 )
## Likelihood ratio test= 0.16  on 1 df,   p=0.7
## Wald test                = 0.16  on 1 df,   p=0.7
## Score (logrank) test = 0.16  on 1 df,   p=0.7
```

© 2023 Nolley EP et al. *JAMA Network Open.*

```
## Single variable

## Call:
## coxph(formula = formula, data = datain1)
##
##   n= 1825, number of events= 1160
##    (21 observations deleted due to missingness)
##
##                          coef exp(coef) se(coef)      z Pr(>|z|)
## factor(ethnicity2)1 -0.1417    0.8679   0.1056 -1.342     0.18
##
##                      exp(coef) exp(-coef) lower .95 upper .95
## factor(ethnicity2)1     0.8679      1.152    0.7056     1.067
##
## Concordance= 0.51  (se = 0.004 )
## Likelihood ratio test= 1.87  on 1 df,   p=0.2
## Wald test            = 1.8  on 1 df,   p=0.2
## Score (logrank) test = 1.8  on 1 df,   p=0.2

## Single variable

## Call:
## coxph(formula = formula, data = datain1)
##
##   n= 1846, number of events= 1166
##
##               coef exp(coef) se(coef)     z Pr(>|z|)
## sofa_calc 0.004312  1.004322 0.011593 0.372     0.71
##
##           exp(coef) exp(-coef) lower .95 upper .95
## sofa_calc     1.004     0.9957    0.9818     1.027
##
## Concordance= 0.506  (se = 0.009 )
## Likelihood ratio test= 0.14  on 1 df,   p=0.7
## Wald test            = 0.14  on 1 df,   p=0.7
## Score (logrank) test = 0.14  on 1 df,   p=0.7

## Single variable

## Call:
## coxph(formula = formula, data = datain1)
##
##   n= 1846, number of events= 1166
##
##                 coef exp(coef) se(coef)      z Pr(>|z|)
## charlson_mv -0.05721   0.94440  0.01052 -5.436 5.45e-08 ***
## ---
## Signif. codes: 0 '***' 0.001 '**' 0.01 '*' 0.05 '.' 0.1 ' ' 1
##
##             exp(coef) exp(-coef) lower .95 upper .95
## charlson_mv 0.9444        1.059   0.9251   0.9641 ##
## Concordance= 0.542  (se = 0.009 )
```

© 2023 Nolley EP et al. *JAMA Network Open.*

```
## Likelihood ratio test= 31.45  on 1 df,   p=2e-08 ##
Wald test                  = 29.55  on 1 df,   p=5e-08
## Score (logrank) test = 29.64  on 1 df,   p=5e-08

## Single variable

## Call:
## coxph(formula = formula, data = datain1)
##
##   n= 1846, number of events= 1166
##
##                 coef exp(coef) se(coef)      z Pr(>|z|)
## pafi_mv0_iqr 0.19688   1.21759  0.02379 8.274   <2e-16 ***
## ---
## Signif. codes: 0 '***' 0.001 '**' 0.01 '*' 0.05 '.' 0.1 ' ' 1
##
##              exp(coef) exp(-coef) lower .95 upper .95
## pafi_mv0_iqr     1.218     0.8213     1.162     1.276
##
## Concordance= 0.616  (se = 0.009 )
## Likelihood ratio test= 55.59  on 1 df,   p=9e-14
## Wald test            = 68.46  on 1 df,   p=<2e-16
## Score (logrank) test = 68.05  on 1 df,   p=<2e-16

## Single variable

## Call:
## coxph(formula = formula2, data = datain1)
##
##   n= 1786, number of events= 1151
##    (60 observations deleted due to missingness)
##
##                          coef exp(coef)  se(coef)      z Pr(>|z|)
## factor(group2)1     -0.307560  0.735239  0.070015 -4.393 1.12e-05 ***
## age_admit_iqr       -0.277093  0.757984  0.038340 -7.227 4.93e-13 ***
## factor(ma0fe1)2     -0.070582  0.931852  0.060449 -1.168  0.24296
## bmi_iqr              0.072837  1.075555  0.026599  2.738  0.00618 **
## factor(race2)1      -0.005153  0.994860  0.062616 -0.082  0.93441
## factor(ethnicity2)1 -0.098501  0.906195  0.114172 -0.863  0.38828
## sofa_calc            0.009107  1.009149  0.012412  0.734  0.46310
## charlson_mv         -0.060995  0.940828  0.010935 -5.578 2.43e-08 ***
## pafi_mv0_iqr         0.202008  1.223858  0.026933  7.500 6.36e-14 ***
## ---
## Signif. codes:  0 '***' 0.001 '**' 0.01 '*' 0.05 '.' 0.1 ' ' 1
##
##                     exp(coef) exp(-coef) lower .95 upper .95
## factor(group2)1        0.7352     1.3601    0.6410    0.8434
## age_admit_iqr          0.7580     1.3193    0.7031    0.8171
## factor(ma0fe1)2        0.9319     1.0731    0.8277    1.0491
## bmi_iqr                1.0756     0.9298    1.0209    1.1331
## factor(race2)1         0.9949     1.0052    0.8800    1.1248
## factor(ethnicity2)1    0.9062     1.1035    0.7245    1.1335
## sofa_calc              1.0091     0.9909    0.9849    1.0340
```

© 2023 Nolley EP et al. *JAMA Network Open.*

```
## charlson_mv               0.9408    1.0629    0.9209    0.9612
## pafi_mv0_iqr              1.2239    0.8171    1.1609    1.2902
##
## Concordance= 0.631  (se = 0.009 )
## Likelihood ratio test= 195.4  on 9 df,   p=<2e-16 ##
Wald test                 = 208.1  on 9 df,   p=<2e-16
## Score (logrank) test = 210  on 9 df,   p=<2e-16

## Sampling weight adjusted propensity match with doubly robust estimation

## Call:
## coxph(formula = formula3, data = datain2,  weights = weights,
##     robust = TRUE, cluster = subclass)
##
##   n= 1786, number of events= 1151
##
##                           coef  exp(coef)   se(coef)  robust se      z Pr(>|z|)
## group2              -0.1863801  0.8299580  0.0628898  0.0994122 -1.875   0.0608
## age_admit           -0.0261989  0.9741413  0.0019490  0.0040256 -6.508 7.61e-11
## bmi                  0.0056579  1.0056739  0.0017234  0.0020941  2.702   0.0069
## factor(ma0fe1)2     -0.0626472  0.9392748  0.0647577  0.0893645 -0.701   0.4833
## charlson_mv         -0.0083913  0.9916438  0.0120760  0.0217753 -0.385   0.7000
## pafi_mv0             0.0021140  1.0021162  0.0002450  0.0004333  4.879 1.07e-06
## sofa_calc           -0.0087092  0.9913286  0.0126613  0.0218452 -0.399   0.6901
## factor(race2)1       0.0177615  1.0179202  0.0664190  0.1232631  0.144   0.8854
## factor(ethnicity2)1 -0.2623225  0.7692629  0.0869728  0.2514335 -1.043   0.2968
##
## group2              .
## age_admit           ***
## bmi                 **
## factor(ma0fe1)2
## charlson_mv
## pafi_mv0            ***
## sofa_calc
## factor(race2)1
## factor(ethnicity2)1
## ---
## Signif. codes:  0 '***' 0.001 '**' 0.01 '*' 0.05 '.' 0.1 ' ' 1
##
##                     exp(coef) exp(-coef) lower .95 upper .95
## group2                 0.8300    1.2049    0.6830    1.0085
## age_admit              0.9741    1.0265    0.9665    0.9819
## bmi                    1.0057    0.9944    1.0016    1.0098
## factor(ma0fe1)2        0.9393    1.0647    0.7884    1.1191
## charlson_mv            0.9916    1.0084    0.9502    1.0349
## pafi_mv0               1.0021    0.9979    1.0013    1.0030
## sofa_calc              0.9913    1.0087    0.9498    1.0347
## factor(race2)1         1.0179    0.9824    0.7995    1.2961
## factor(ethnicity2)1    0.7693    1.2999    0.4700    1.2592
##
## Concordance= 0.651  (se = 0.024 )
## Likelihood ratio test= 288.1   on 9 df,   p=<2e-16
## Wald test             = 118.8   on 9 df,   p=<2e-16
## Score (logrank) test = 317.3   on 9 df,   p=<2e-16,   Robust = 71.59   p=7e-12
```

© 2023 Nolley EP et al. *JAMA Network Open.*

```
##
##   (Note: the likelihood ratio and score tests assume independence of
##      observations within a cluster, the Wald and robust score tests do not).

##                                               Single variable Multiple variable
## 1                                        COVID-19 0.71 (0.63-0.80)   0.74  (0.64-0.84)
## 2                      Age in years (IQR range) 0.72 (0.67-0.77)   0.76  (0.70-0.82)
## 3                                        Male sex 0.88 (0.78-0.98)   0.93  (0.83-1.05)
## 4 Body mass index  in Kg/m² (IQR range) 1.06 (1.01-1.12)   1.08 (1.02-1.13)
## 5                                Non-white range 0.98 (0.87-1.10)   0.99 (0.88-1.12)
## 6                             Hispanic ethnicity 0.87 (0.71-1.07)   0.91 (0.72-1.13)
## 7                                      SOFA score 1.00 (0.98-1.03)   1.01 (0.98-1.03)
## 8                  Charlson comorbidity index 0.94 (0.93-0.96)    0.94  (0.92-0.96)
## 9                         PaO/FiO (IQR range) 1.22 (1.16-1.28)    1.22  (1.16-1.29)
##  After full matching
## 1     0.83 (0.68-1.01)
## 2
## 3
## 4
## 5
## 6
## 7
## 8
## 9
```

```
death.p <- sprintf("%3.2f",tidy(coxph(Surv(time2death,died) ~group2,
                        data = df.matched ,
                        robust = TRUE,
                        weights = weights,
                        cluster = subclass))$p.value)

message("p-value for propensity-matched models for death at 90 days ")
```

```
## p-value for propensity-matched models for death at 90 days
```

```
print(death.p)
```

```
## [1] "0.85"
```

```
fun.reg = function(yr){

df.tm = dff %>%
  filter(!is.na(bmi) & !is.na(age_admit) & !is.na(paficat) & !is.na(ma0fe1) &
 ↪ !is.na(charlson_mv)   &
             !is.na(sofa_calc) & !is.na(race2) & !is.na(ethnicity2)) %>%
  dplyr::mutate(year = substr(id, 38, 41)) %>%
  dplyr::filter(year %in% c(yr, "2020", "2021"))

set.seed(443527)
### Sampling weight adjusted propensity match estimation
mo <-     matchit(ppnsity.model, data =df.tm, method = "full")
```

© 2023 Nolley EP et al. *JAMA Network Open.*

```
mean.dis = paste0(sprintf("%3.3f", mean(mo$distance, na.rm = TRUE)))

df.xx <- match.data(mo, distance = "prop.score")


df.xx$ma1fe2 = ifelse(df.xx$ma0fe1==0, 2, df.xx$ma0fe1)

ft1 = glm(dead ~ as.factor(group), data = df.xx, weights = weights,
              family = quasibinomial(link = "logit"))

yy = coeftest(ft1, vcov. = vcovCL, cluster = ~subclass)

a = paste0(sprintf("%3.2f", exp(coef(yy))[2]) , " (",
           sprintf("%3.2f", exp(confint(yy))[2,1]) , " - ",
           sprintf("%3.2f", exp(confint(yy))[2,2]), ")")

formula.invent = as.formula(Surv(timeinvent, invent) ~ group2)
formula.dis = as.formula(Surv(time2dis, dis) ~ group2)


ftt1 = summary(coxph(formula.invent, data = df.xx, robust = TRUE, weights = weights,
 ↪  cluster = subclass))
ftt2 = summary(coxph(formula.dis, data = df.xx, robust = TRUE, weights = weights, cluster
 ↪  = subclass))

b = paste0(sprintf("%3.2f", ftt1$conf.int[1]), " (", sprintf("%3.2f", ftt1$conf.int[3]),
 ↪  " - ", sprintf("%3.2f", ftt1$conf.int[4]), ")")

c = paste0(sprintf("%3.2f", ftt2$conf.int[1]), " (", sprintf("%3.2f", ftt2$conf.int[3]),
 ↪  " - ", sprintf("%3.2f", ftt2$conf.int[4]), ")")

return(list(c(a, b, c), mean.dis))
}

tab.reg = rbind(fun.reg("2016")[[1]], fun.reg("2017")[[1]], fun.reg("2018")[[1]],
 ↪  fun.reg("2019")[[1]])

colnames(tab.reg) = c("OR death", "HR time on MV", "HR discharge")
rownames(tab.reg) = c("2016", "2017", "2018", "2019")
write.csv(tab.reg,   "tab.reg.csv")
```

Mean distance while matched with each year's of control group from 2016 to 2019

```
tab.mean.dis =  rbind(fun.reg("2016")[[2]], fun.reg("2017")[[2]], fun.reg("2018")[[2]],
 ↪  fun.reg("2019")[[2]])
colnames(tab.mean.dis) = c("Mean distance after full matching")
rownames(tab.mean.dis) = c("2016", "2017", "2018", "2019")
tab.mean.dis %>% kable() %>% kable_classic_2(full_width = FALSE)
```

© 2023 Nolley EP et al. *JAMA Network Open.*

	Mean distance after full matching
2016	0.844
2017	0.720
2018	0.689
2019	0.651

	OR death	HR time on MV	HR discharge
2016	0.52 (0.16 - 1.67)	0.75 (0.52 - 1.09)	0.69 (0.46 - 1.04)
2017	1.56 (0.70 - 3.52)	1.17 (0.62 - 2.20)	1.15 (0.60 - 2.17)
2018	1.05 (0.56 - 1.97)	0.89 (0.57 - 1.39)	0.88 (0.56 - 1.38)
2019	0.94 (0.53 - 1.66)	0.85 (0.60 - 1.22)	0.81 (0.55 - 1.21)

Regrssion outcome while matched with each year's of control group from 2016 to 2019

```
tab.reg %>% kable() %>% kable_classic_2(full_width = FALSE)
```

Sensitivity analysis: before vs after the date when the RECOVERY trial was published

```
df.s1 = dff %>%
  filter(!is.na(bmi) & !is.na(age_admit) & !is.na(paficat) & !is.na(ma0fe1) &
 ↪ !is.na(charlson_mv) &
           !is.na(sofa_calc) & !is.na(race2) & !is.na(ethnicity2)) %>%
  dplyr::rename(hospdate = hospdate.x)

df.s1$recovery <- ifelse(df.s1$hospdate < mdy(07172020), "before_dexa", "after_dexa")

middate <- as.Date(min(df.s1$hospdate[df.s1$group == "COVID19"])) +
  days(round(difftime(max(df.s1$hospdate[df.s1$group  ==  "COVID19"]),
                      min(df.s1$hospdate[df.s1$group == "COVID19"]),
                      units = "days")/2))

df.s1$mid <- ifelse(as.Date(df.s1$hospdate) < middate, "before_mid", "after_mid")

set.seed(443527)
ms1 <-   matchit(ppnsity.model, data = df.s1, method = "full")

df.s1 <- match.data(ms1, distance = "prop.score")
df.s1$ma1fe2 = ifelse(df.s1$ma0fe1==0, 2, df.s1$ma0fe1)

fit.s1 = glm(dead ~ as.factor(group) + as.factor(recovery), data = df.s1, weights =
 ↪ weights,
             family = quasibinomial(link = "logit"))

print(summary(fit.s1))
```

```
##
```

© 2023 Nolley EP et al. *JAMA Network Open.*

```
## Call:
## glm(formula = dead ~ as.factor(group) + as.factor(recovery),
##       family = quasibinomial(link = "logit"), data = df.s1, weights = weights)
##
## Deviance  Residuals:
## Min                 1Q Median             3Q         Max
##  -6.8041     -0.9755     -0.4619      0.7777     11.4034
##
## Coefficients:
##                                         Estimate Std. Error t value Pr(>|t|)
## (Intercept)                            -0.322350    0.102943  -3.131   0.00177 **
## as.factor(group)Non-COVID19        0.005472    0.132977     0.041   0.96718
## as.factor(recovery)before_dexa -0.172971 0.156128  -1.108   0.26806
## ---
## Signif. codes:  0 '***' 0.001 '**' 0.01 '*' 0.05 '.' 0.1 ' ' 1
##
## (Dispersion  parameter  for  quasibinomial  family  taken  to  be  1.001688)
##
##      Null deviance: 2386.2   on 1785   degrees of freedom ##
Residual  deviance:  2384.2    on  1783    degrees  of  freedom  ##
AIC:  NA
##
## Number of Fisher Scoring iterations: 4
```

```
print(coeftest(fit.s1,  vcov.  =  vcovCL,  cluster  =  ~subclass))
```

```
##
## z  test  of  coefficients:
##
##                                          Estimate  Std.  Error  z  value  Pr(>|z|)
## (Intercept)                            -0.3223502   0.1102278 -2.9244  0.003451 **
## as.factor(group)Non-COVID19        0.0054723     0.2187277     0.0250   0.980040
## as.factor(recovery)before_dexa -0.1729712 0.1518574 -1.1390 0.254688
## ---
## Signif.  codes:  0  '***'  0.001  '**'  0.01  '*'  0.05  '.'  0.1  ' '  1
```

Sensitivity analysis: First vs second half of pandemic

```
fit.s2 = glm(dead ~ as.factor(group) + as.factor(mid), data = df.s1, weights = weights,
                  family  =  quasibinomial(link  =  "logit"))

print(summary(fit.s2))
```

```
##
## Call:
## glm(formula  =  dead  ~  as.factor(group)  +  as.factor(mid),  family  =  quasibinomial(link  =  "logit"),
##       data  =  df.s1,  weights  =  weights)
##
## Deviance  Residuals:
##       Min          1Q     Median          3Q         Max
```

© 2023 Nolley EP et al. *JAMA Network Open.*

```
## -6.8041   -0.9626   -0.4619    0.7777   11.4034
##
## Coefficients:
##                               Estimate Std. Error t value Pr(>|t|)
## (Intercept)                   -0.26548    0.10935  -2.428   0.0153 *
## as.factor(group)Non-COVID19    0.03899    0.12635   0.309   0.7576
## as.factor(mid)before_mid      -0.26337    0.15498  -1.699   0.0894 .
## ---
## Signif. codes:  0 '***' 0.001 '**' 0.01 '*' 0.05 '.' 0.1 ' ' 1
##
## (Dispersion parameter for quasibinomial family taken to be 1.001688)
##
##     Null deviance: 2386.2  on 1785  degrees of freedom ##
Residual deviance: 2382.5  on 1783  degrees of freedom ##
AIC: NA
##
## Number of Fisher Scoring iterations: 4
```

```
print(coeftest(fit.s2, vcov. = vcovCL, cluster = ~subclass))
```

```
##
## z test of coefficients:
##
##                               Estimate Std. Error z value Pr(>|z|)
## (Intercept)                   -0.265478   0.122536 -2.1665  0.03027 *
## as.factor(group)Non-COVID19    0.038995   0.202732  0.1923  0.84747
## as.factor(mid)before_mid      -0.263366   0.166879 -1.5782  0.11452
## ---
## Signif. codes:  0 '***' 0.001 '**' 0.01 '*' 0.05 '.' 0.1 ' ' 1
```

```
message("Average time to death for patients with PaO /Fi0    150 mm Hg:")
```

```
## Average time to death for patients with PaO /FiO    150 mm Hg:
```

```
tidy(coxph(Surv(time2death, died) ~ factor(group2), data = dff[dff$paficat == 1,]),
 ↪ conf.int = TRUE, exponentiate = TRUE)
```

```
## # A tibble: 1 x 7
## term              estimate std.error statistic p.value conf.low conf.high
## <chr>                <dbl>     <dbl>     <dbl>   <dbl>    <dbl>     <dbl> ##
1 factor(group2)1     0.977     0.114    -0.201   0.841    0.782        1.22
```

```
message("Average time to death for patients with PaO /Fi0  > 150 mm Hg:")
```

```
## Average time to death for patients with PaO /FiO  > 150 mm Hg:
```

```
tidy(coxph(Surv(time2death, died) ~ factor(group2), data = dff[dff$paficat == 0,]),
 ↪ conf.int = TRUE, exponentiate = TRUE)
```

© 2023 Nolley EP et al. *JAMA Network Open.*

```
## # A tibble: 1 x 7
## term                estimate std.error statistic p.value conf.low conf.high
## <chr>                  <dbl>     <dbl>     <dbl>   <dbl>    <dbl>     <dbl> ##
1 factor(group2)1        1.05      0.112     0.418   0.676    0.842      1.30
```

```
message("Average time to discharge for patients with PaO /Fi0    150 mm Hg:")
```

```
## Average time to discharge for patients with PaO /FiO    150 mm Hg:
```

```
tidy(coxph(Surv(time2dis, dis) ~ factor(group2), data = dff[dff$paficat == 1,]), conf.int
↪  = TRUE, exponentiate = TRUE)
```

```
## # A tibble: 1 x 7
## term                estimate std.error statistic p.value conf.low conf.high
## <chr>                  <dbl>     <dbl>     <dbl>   <dbl>    <dbl>     <dbl> ##
1 factor(group2)1       0.790      0.117     -2.03  0.0426    0.628     0.992
```

```
message("Average time to discharge for patients with PaO /Fi0  > 150 mm Hg:")
```

```
## Average time to discharge for patients with PaO /FiO  > 150 mm Hg:
```

```
tidy(coxph(Surv(time2dis, dis) ~ factor(group2), data = dff[dff$paficat == 0,]), conf.int
↪  = TRUE, exponentiate = TRUE)
```

```
## # A tibble: 1 x 7
## term                estimate std.error statistic p.value conf.low conf.high
## <chr>                  <dbl>     <dbl>     <dbl>   <dbl>    <dbl>     <dbl> ##
1 factor(group2)1       0.829     0.0748     -2.50  0.0124    0.716     0.960
```

© 2023 Nolley EP et al. *JAMA Network Open.*

eAppendix 5. Boxplots of Tidal Volume, Positive End-Expiratory Pressure, and Plateau Pressure by Calendar Year Between 2016 and 2019

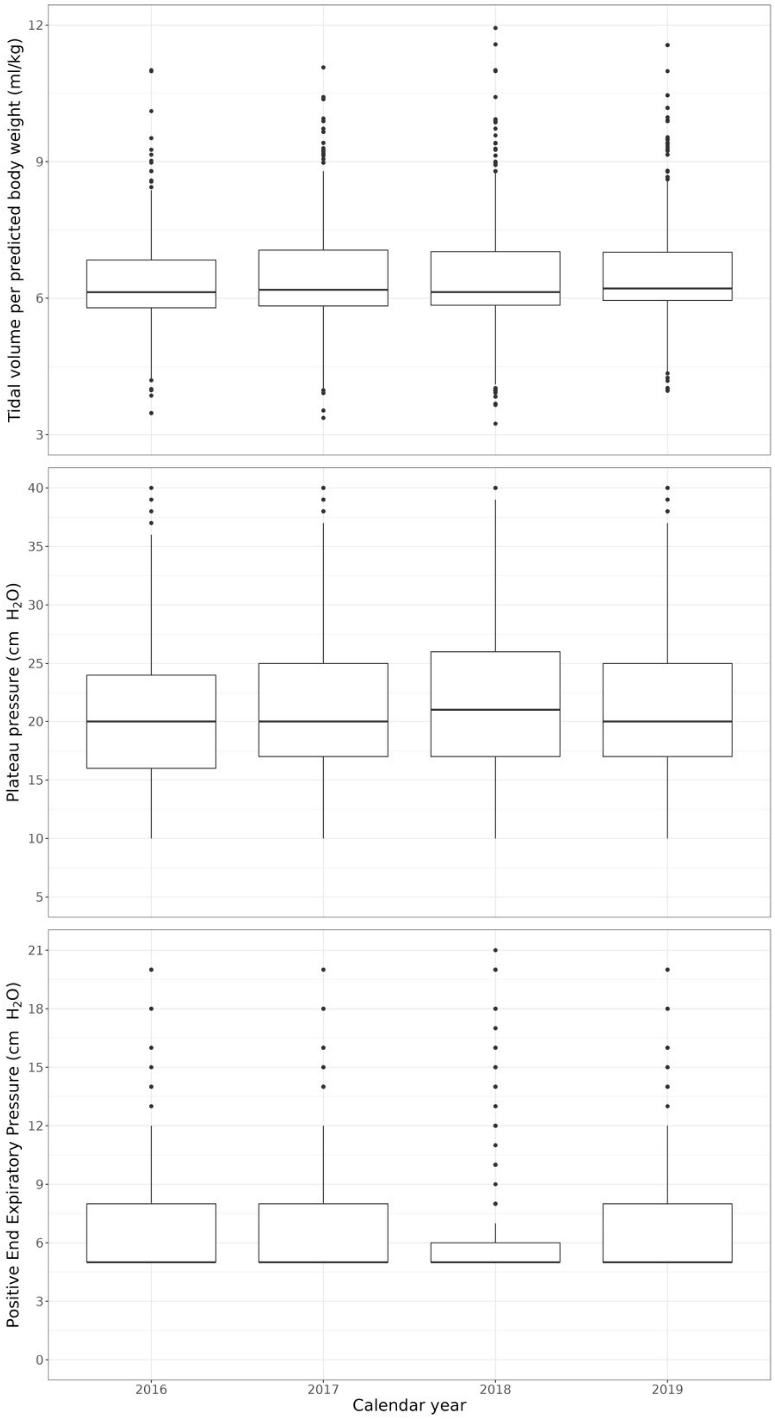

© 2023 Nolley EP et al. *JAMA Network Open.*

eAppendix 6. Love Plot Comparing Absolute Standardized Mean Differences for Key Variables Between Patients With Severe COVID-19 and Non–COVID-19 Pneumonia Before and After Full Optimal Propensity Score Matching

The Love plot is a summary plot of covariate balance before (in black) and after (in grey) propensity score matching. The Love plot demonstrates if covariate balance has improved after propensity score matching. The black dots indicate the standardized mean difference for key factors before matching and grey dots indicate the standardized mean difference for key factors after matching between patients with COVID-19 and non-COVID-19. Factors were considered balanced if the standardized mean difference between groups was between -0.1 and 0.1 (shown as broken vertical lines). The thresholds are indicated by vertical broken lines. Legend: SOFA = Sequential Organ Failure Score, PaO_2/FiO_2 = partial pressure of oxygen divided by FiO_2 = fraction of inspired oxygen.

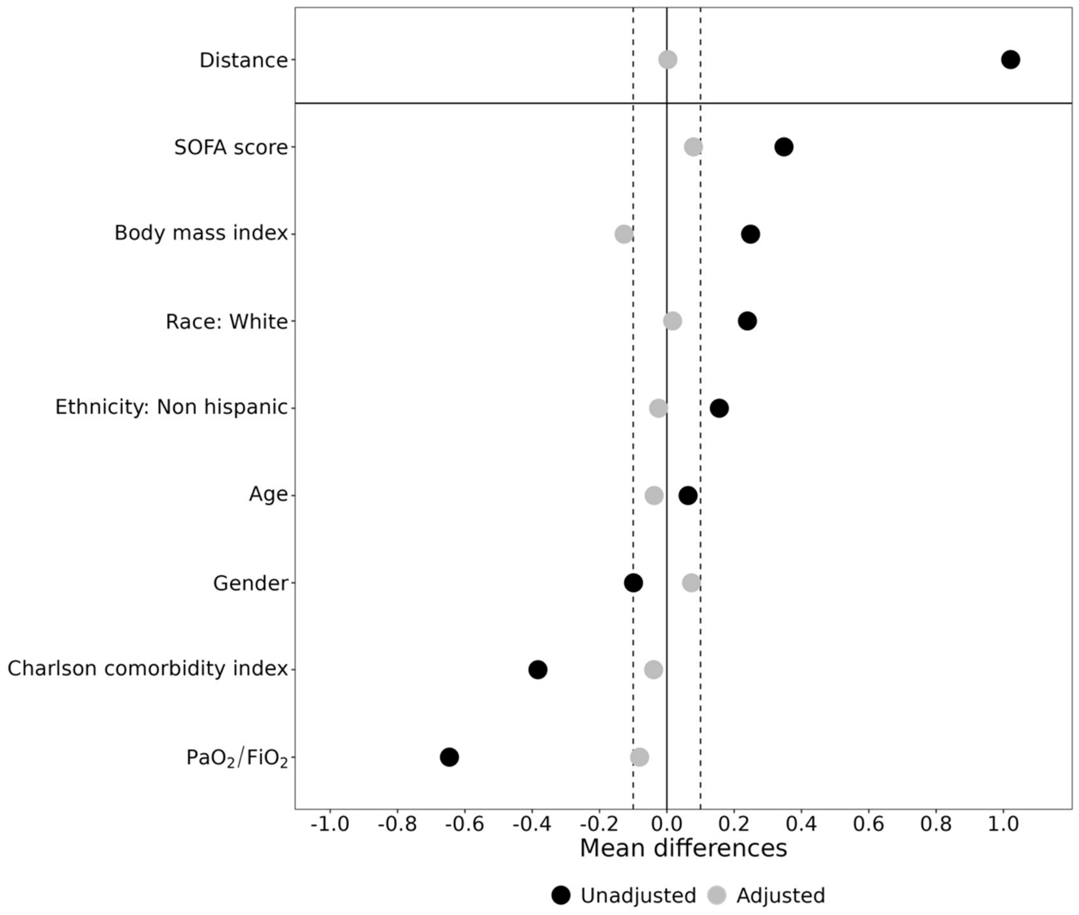

© 2023 Nolley EP et al. *JAMA Network Open.*

eAppendix 7. Odds Ratio of Hospital Mortality and Subdistribution Hazard Ratios of Times on Mechanical Ventilation and Hospital Discharge Among Those Alive When Matched Sets for Propensity Score Adjustment Was Limited to Patients With Non–COVID-19 for Any Single Calendar Year Between 2016 and 2019

	**Hospital mortality**	**Time on Mechanical Ventilation**	**Time to hospital discharge**
2016	0.52 (0.16 - 1.67)	0.75 (0.52 - 1.09)	0.69 (0.46 - 1.04)
2017	1.56 (0.70 - 3.52)	1.17 (0.62 - 2.20)	1.15 (0.60 - 2.17)
2018	1.05 (0.56 - 1.97)	0.89 (0.57 - 1.39)	0.88 (0.56 - 1.38)
2019	0.94 (0.53 - 1.66)	0.85 (0.60 - 1.22)	0.81 (0.55 - 1.21)

© 2023 Nolley EP et al. *JAMA Network Open.*
