# Supplementary material for: Outcomes Among Mechanically Ventilated Patients With Severe Pneumonia and Acute Hypoxemic Respiratory Failure From SARS-CoV-2 and Other Etiologies
Source: JAMA Netw Open. 2023 Jan 10;6(1):e2250401. doi: 10.1001/jamanetworkopen.2022.50401 (PMC9856712; doi:10.1001/jamanetworkopen.2022.50401)
Supplement: Supplement 2. — Data Sharing Statement [file jamanetwopen-e2250401-s002.pdf]

Data Sharing Statement

Nolley. Outcomes Among Mechanically Ventilated Patients With Severe Pneumonia and Acute Hypoxemic Respiratory Failure From SARS-CoV-2 and Other Etiologies. *JAMA Netw Open*. Published January 10, 2023. doi:10.1001/jamanetworkopen.2022.50401

Data
Data available: Yes
Data types: Deidentified participant data, Data dictionary
How to access data: Data will be made available upon reasonable request to the corresponding author. Please email wcheckl1@jhmi.edu for questions.
When available: With publication

Supporting Documents
Document types: Statistical/analytic code
How to access documents: Statistical code is available in the Online Supplement.
When available: With publication

Additional Information
Who can access the data: Researchers whose proposed use of the data has been approved.
Types of analyses: Data will be made available for the purposes of replication.
Mechanisms of data availability: Data will be made available after approval of a proposal and a signed data access agreement.
